# Circular RNA circPGD contributes to gastric cancer progression via the sponging miR-16-5p/ABL2 axis and encodes a novel PGD-219aa protein

**DOI:** 10.1038/s41420-022-01177-0

**Published:** 2022-09-14

**Authors:** Yun Liu, Jia Cao, Linqi Zhu, Wenjun Zhao, Yong Zhou, Chen Shao, Shihe Shao

**Affiliations:** 1 School of Medicine, Jiangsu University, Zhenjiang, 212013 Jiangsu China; 2 Endoscopy Center, Department of Gastroenterology, Shanghai East Hospital, School of Medicine, Tongji University, Shanghai, 200120 China; 3 Department of Digestive, the Affiliated People’s Hospital, Jiangsu University, Zhenjiang, 212002 Jiangsu China; 4 Department of Cardiology, Affiliated Hospital of Jiangsu University, Zhenjiang, 212013 Jiangsu China

**Keywords:** Gastric cancer, Gastric cancer

## Abstract

CircRNAs have critical effects on tumor development and progression. However, circPGD effect on gastric cancer (GC) is still elusive. Nuclear and cytoplasmic RNA fractionation, and RNA-FISH assay examined the localization of circPGD in MGC-803 cells. qRT-PCR was conducted to detect the expression and prognostic significance of circPGD, miR-16-5p, and ABL2 within GC tissues. Meanwhile, qRT-PCR, luciferase reporter assays, rescue, and western blotting assays confirmed the interactions between circPGD, miR-16-5p, and ABL2. Transwell, wound healing, and colony-formation assays, as well as CCK-8 and cell apoptosis assays, analyzed the functions of circPGD, miR-16-5p, ABL2, as well as PGD-219aa within GC cells. Western blotting and cell immunofluorescence experiments detected the differences in the expression of the related proteins. Finally, xenograft and metastatic mouse models were used to investigate circPGD function in vivo. Mass spectrometry was used to detect the existence of PGD-219aa in MGC-803 cells. CircPGD was localized in the cytoplasm and nucleus of MGC-803 cells. Compared with the control, circPGD and ABL2 expression increased within GC tissues and cells, and the miR-16-5p level was decreased. Functionally, circPGD promoted cell proliferation, migration and suppressed apoptosis in vitro. Mechanistically, circPGD sponged miR-16-5p for relieving miR-16-5p suppression on the corresponding target ABL2 via the SMAD2/3 and YAP signaling pathways. In addition, circPGD encodes a novel PGD-219aa protein that can enhance the growth and migration of GC cells, while inhibiting GC cells apoptosis via the SMAD2/3 and YAP signaling pathways. Furthermore, circPGD overexpression enhanced tumor aggressiveness, while circPGD knockdown inhibited tumor growth. Overall, circPGD has a novel oncogenic effect on GC cells, indicating the potential of circPGD as the tumorigenic factor and a promising diagnostic marker for GC.

## Introduction

Gastric cancer (GC) represents the third deadliest malignancy globally, which ranks fifth in terms of occurrence frequency [1–3]. GC is a multifactorial and complex disease, which is mainly caused by the regional environment and dietary factors, infection with *Helicobacter pylori*, as well as various genetic factor [4–7]. As such, examining the molecular mechanisms is urgently need to further explaining GC initiation and development.

Recently, non-coding RNAs (ncRNAs) are increasingly reported to mediate GC occurrence and progression. CircRNAs represent the new type of ncRNAs that have the closed circular loop. CircRNAs have the covalently-closed loop, and there is one back-splice site between 3′- and 5′-end, different compared with the formation of linear RNA [8, 9]. CircNRIP1 decreases cell growth, invasion, migration, and AKT1 expression within GC cells, while miR-149-5p blocks circNRIP1 malignant behaviors [10]. CircRNA_100269 and circRNA_001569 expression increased within GC tissues, where they regulate miRNAs and related proteins expression that modulate cell viability and apoptosis [11, 12]. Phosphogluconate dehydrogenase (*PGD*) is a protein-coding gene, as suggested by the public databases circBase (http://www.circbase.org/) and circBANK (http://www.circbank.cn/), it formed some circRNAs through exonal back-splicing in linear transcript. Although there have been studies on the role of *PGD* in ovarian, lung, and gastrointestinal stromal cancer [13, 14], no relevant studies studied the role of circRNAs from which it is derived in related diseases. Thus, this study spliced circPGD, whose circBase ID was hsa_circ_0009735, in *PGD* gene at chr1: 10477043-10480201 while forming the sense-overlapping circular transcript that was 1003 nucleotides long.

CircRNAs may regulate biological processes by acting as microRNAs (miRNAs) sponges, binding to the RNA-binding proteins (RBPs), encoding proteins or peptides, and regulating gene transcription. MiRNAs can regulate post-transcriptional gene expression, reduce mRNA stability, and inhibit translation [15]. Database analysis showed that circPGD may act as miR-16-5p sponges. Melatonin administration elevates miR-16-5p expression, which can specifically target SMAD3 with negative regulation on its abundance, highlighting that miR-16-5p/SMAD3 interactions have important effects on melatonin-induced growth defects within GC [16]. As reported by Zhang et al., miR-16-5p expression decreased within GC cases and related to TNM stages and differentiation grades, particularly in cases of early cancer [17]. However, the mechanism underlying miR-16-5p within GC remains largely unclear. The ABL member of the non-receptor tyrosine kinases family ABL2 (also known as Arg, Abelson-Related Gene) has an important effect on several solid tumors development [18]. ABL2 expression and function have been studied in hepatocellular carcinoma, colorectal cancer, lung adenocarcinoma, and melanoma [19–23]. Interestingly, Marchetti et al. reported that ABL2 is a potential target to relieve the endothelial damage caused by COVID-19 [24]. Also, the robust expression of ABL2 can suppress apoptosis in GC cells [25].

In this study, a novel circRNA circPGD was identified from the exons of 9–13 of the liner transcript of *PGD*. CircPGD expression increased within GC tissues and cells, which was related to lymph node metastasis (LNM) and tumor size. Importantly, circPGD enhanced cell growth and metastasis but inhibited cell apoptosis through sponging miR-16-5p for preventing the suppression on its target ABL2 in GC cells. In addition, circPGD encoded a novel PGD-219aa protein that enhanced the malignant behaviors of GC cell. It also enhanced the biological behaviors of GC cells by regulating EMT via SMAD2/3 and YAP signaling pathways. Overall, circPGD acts as an oncogene during GC development via the miR-16-5p/ABL2 axis, which encodes the PGD-219aa protein, indicating its role as the novel biomarker for diagnosing GC patients.

## Results

### CircPGD is upregulated within GC tissues and cells

**Fig. 1:**
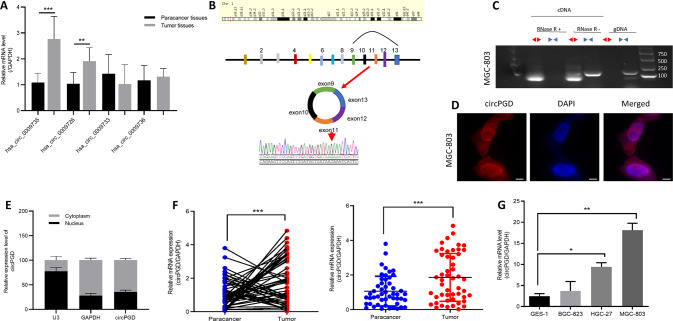
CircPGD expression increases within GC cells and tissues. **A** Expression levels of PGD-derived circRNAs in GC tissues. **B** Sketch map showing circPGD forming by exonal circularization within *PGD* gene. According to PCR product sequencing, circPGD was spliced from the head to the tail. **C** Gel electrophoresis suggested that *PGD* existed in the canonical and back-spliced type with/without RNaseR application within gDNA and cDNA in GC cells. **D** and **E** Nuclear-cytoplasmic fractionation and RNA-FISH assay revealed the cytoplasmic and nuclear location of circPGD in MGC-803 cells, scale bars = 25 μm. **F** Relative circPGD level in 50 paired fresh normal gastric tissues and GC tissues. **G** qRT-PCR detected circPGD levels within GES-1 cells as well as three GC cell lines. *p < 0.05, **p < 0.01, ***p < 0.001.

CircPGD was back-spliced from the *PGD* gene and formed a sense-overlapping circular transcript. The expression levels of four circRNA derived from *PGD* and found hsa_circ_0009735 was upregulated obviously in eight GC patients (Fig. 1A), and the PCR product was sequenced and found it was matched with hsa_circ_0009735 and has the cyclization sites (Fig. 1B). PCR and agarose gel electrophoresis were conducted using customized convergent and divergent primers for examining canonical and back-spliced PGD expression with/without RNAse R supplementation within gDNA and cDNA of GC cells. The results showed that circPGD resisted RNAse R digestion (Fig. 1C), while the linear PGD was not subject to convergent primer amplification, indicating the non-involvement of circPGD in PCR artifacts or genomic rearrangements. In addition, according to cytoplasmic and nuclear RNA fractionation and RNA-FISH assay, circPGD was localized at both the cytoplasm and nucleus of MGC-803 cells (Fig. 1D, E).

CircPGD levels were measured within 50 freshly frozen GC tissues as well as 50 matched paracancer samples, showed that circPGD was upregulated within GC samples compared with paracancer samples (Fig. 1F), which was related to LNM and tumor size (Table 1). Furthermore, the circPGD level was upregulated within GC cells compared with GES-1 cells, and it was highest in MGC-803 cells. By contrast, BGC-823 cells had the lowest expression level (Fig. 1G). Based on the above findings, circPGD level was higher within GC cell line and GC patients.

**Table 1:** Relation between circPGD and miR-16-5p levels and the GC clinical features.

	circPGC expression				miR-16-5p expression			
Characteristics	Case	High (*n* = 28)	Low (*n* = 22)	*p* value	case	High (*n* = 21)	Low (*n* = 29)	*p* value
Gender				ns				0.3557
Male	35	20	15		35	13	22	
Female	15	8	7		15	8	7	
Age (years)				ns				0.5261
>65	38	21	17		38	17	21	
≤65	12	7	5		12	4	8	
Tumor size				**0.042**				**0.0215**
>4	29	20	9		29	8	21	
≤4	21	8	13		21	13	8	
LNM				**0.045**				0.2516
Positive	25	18	7		25	8	17	
Negative	25	10	15		25	13	12	
Clinical stage				0.1536				0.5672
I + II	25	11	14		25	12	13	
III + IV	25	17	8		25	9	16	

Bold values indicates statistical significant *P* values.

### CircPGD enhances GC cell growth and metastasis in vitro

**Fig. 2:**
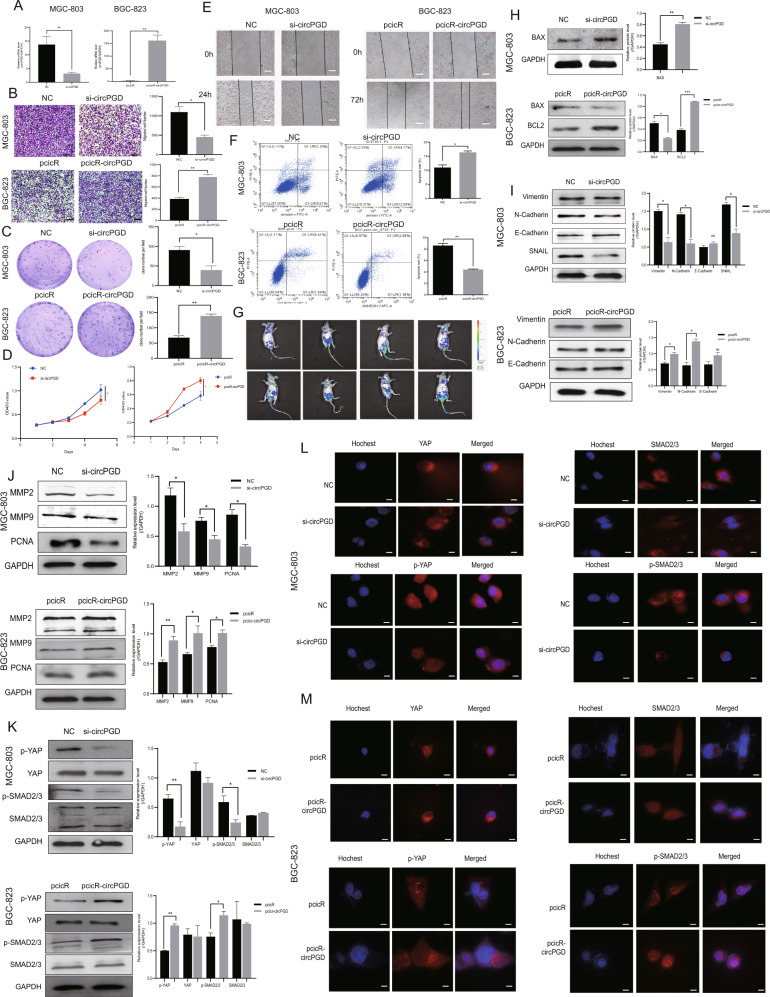
CircPGD enhances GC cell migration and proliferation, and suppresses apoptosis in vitro. **A** qRT-PCR was conducted to detect the efficiency of knockdown and overexpression of circPGD. **B** Transwell migration assay (scale bar = 100 μm), **C** colony-formation assay, **D** CCK-8 assay, **E** wound healing assay (scale bars = 50 μm), and **F** apoptosis assay analyzed the effects of abnormal expression of circPGD on GC cell proliferation, migration and apoptosis. **G** GFP-labeled circPGD was used to analyze tumor metastasis ability with an in vivo imaging system at 4 weeks after tail vein injection of BGC-823 cells (pcicR or pcicR-circPGD). **H–K** Western blotting analyzed protein expression involved in EMT, cell proliferation, migration and apoptosis, and SMAD2/3, p-SMAD2/3, and YAP, p-YAP when circPGD was silenced or overexpressed in GC cells (western blotting strips are cropped, original images can be provided on request). **L** and **M** Immunofluorescence detected expression of YAP, p-YAP, SMAD2/3, and p-SMAD2/3 within the presence of abnormal circPGD expression in GC cells, scale bars = 25 μm. *p < 0.05, **p < 0.01, ***p < 0.001.

The specific siRNA for circPGD and the pircR-circPGD overexpression plasmids were designed, followed by transfection into MGC-803 cells or BGC-823 cells to assess the functions of circPGD. The expression of circPGD was silenced or overexpressed within GC cells (Fig. 2A). According to transwell migration, wound healing, colony-formation, CCK-8 and cell apoptosis assays, circPGD upregulation promoted GC cell growth and metastasis, and decreased apoptosis (Fig. 2B–F). Additionally, in vivo imaging showed that circPGD overexpression in BGC-823 cells could promote lung metastasis compared to control mice (Fig. 2G). Furthermore, circPGD knockdown in MGC-803 cells increased BAX and E-cadherin expression, while N-cadherin, Vimentin and Snail levels were reduced significantly (Fig. 2H, I). Furthermore, the levels of MMP2, MMP9, PCNA, p-YAP, and p-SMAD2/3 decreased, while those of YAP and SMAD2/3 showed no obvious changes in circPGD-silenced MGC-803 cells (Fig. 2J, K). The impact of circPGD in MGC-803 cells was further validated by immunofluorescence experiments (Fig. 2L). The levels of N-cadherin, Vimentin, MMP2, MMP9, PCNA, BCL2, p-YAP, and p-SMAD2/3 were increased in circPGD-overexpressed BGC-823 cells, while E-cadherin and BAX levels were decreased significantly (Fig. 2H–K, M). Overall, high circPGD expression promotes EMT, enhances GC cell growth and migration in vitro, while inhibiting apoptosis.

### CircPGD sponges miR-16-5p

**Fig. 3:**
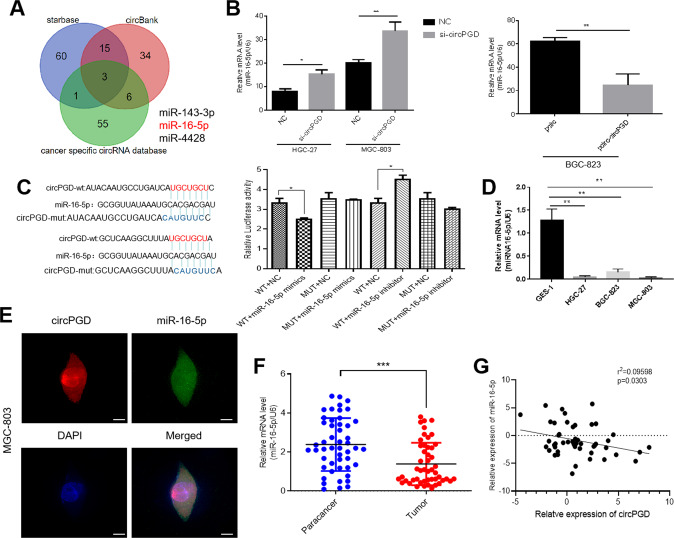
CircPGD sponges miR-16-5p. **A** Three public databases estimated circPGD target miRNAs. **B** qRT-PCR monitored miR-16-5p level after abnormal circPGD level within GC cells. **C** The dual-luciferase reporter assay demonstrated the existence of binding sites in circPGD with miR-16-5p. **D** qRT-PCR detected miR-16-5p level within GES-1 and GC cell lines. **E** Immunofluorescence detected circPGD together with miR-16-5p co-localization within MGC-803 cells, scale bars = 25 μm. **F** The miR-16-5p expression decreased within tissues showing circPGD upregulation compared with circPGD downregulation. **G** Pearson correlation analysis found that circPGD was negatively related to miR-16-5p within 50 paired GC samples. *p < 0.05, **p < 0.01, ***p < 0.001.

Three databases starbase, circbank, and a cancer-specific circRNA database were cross-analyzed to predict circPGD target miRNAs, which obtained miR-16-5p, miR-143-3p, and miR-4428(Fig. 3A). QRT-PCR further detected expression levels of the three miRNAs, demonstrating miR-16-5p upregulation following circPGD knockdown in MGC-803 and HGC-27 cells. By contrast, overexpression of circPGD in BGC-823 resulted in a decrease in miR-16-5p (Fig. 3B). For the remaining miRNAs, they were not closely related to circPGD. A dual-luciferase reporter with wild-type (WT) and mutant (MUT) circPGD in the miR-16-5p binding sites was constructed. 293 T cells were co-transfected with miR-16-5p inhibitor or mimics and luciferase reporter plasmids. Compared with the control, luciferase ratio significantly reduced when cells were subject to miR-16-5p mimics co-transfection. By contrast, the increased luciferase ratio was noted when the cells were subject to miR-16-5p inhibitor co-transfection (Fig. 3C). According to the above findings, the circPGD directly bound to miR-16-5p according to the corresponding complementary sequences. Compared with GES-1 cells, miR-16-5p was downregulated within GC cells (Fig. 3D). According to RNA-FISH assay, miR-16-5p and circPGD showed co-expression within MGC-803 cells (Fig. 3E). According to qRT-PCR assay, miR-16-5p expression decreased within GC tissues, and circPGD negatively related to miR-16-5p expression (Fig. 3F, G). As reported by two studies, miR-16-5p was the tumor suppressor among GC patients [15, 16], but there is no detailed study in GC cells that can illustrate miR-16-5p effect on GC. Furthermore, clinical data were collected from the patients mentioned above, and the miR-16-5p expression showed negative relation to tumor size (Table 1). Overall, circPGD sponged miR-16-5p within the GC cells cytoplasm.

### MiR-16-5p reverses circPGD effect on promoting GC development

**Fig. 4:**
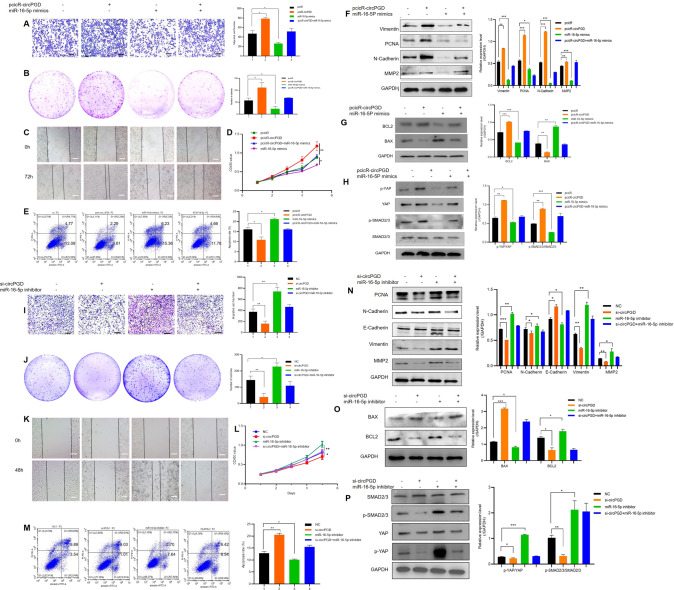
MiR-16-5p reverses circPGD ability to promote GC progression. **A** Transwell migration assay (scale bars = 100 μm), **B** colony-formation, **C** wound healing (scale bars = 50 μm), **D** CCK-8, and **E** apoptosis assays analyzed the impact of circPGD plasmids and miR-16-5p mimics on BGC-823 cell growth, metastasis, and apoptosis. **F** Western blotting assay suggested the role of miR-16-5p mimics in decreasing Vimentin, PCNA, N-Cadherin, and MMP2 levels, which were increased by the transfection of circPGD plasmids. **G** MiR-16-5p mimics abolished circPGD effect on the BCL2 and BAX levels. **H** Impact of circPGD and miR-16-5p mimics on SMAD2/3, p-SMAD2/3, YAP, and p-YAP. **I–M** Roles of si-circPGD and miR-16-5p inhibitor in cell growth, migration as well as apoptosis. **N**, **O**, and **P** Western blotting examined the effects of si-circPGD and miR-16-5p inhibitor role in protein levels (western blot strips are cropped, original images can be provided on request). *p < 0.05, **p < 0.01, ***p < 0.001.

Given circPGD effect on promoting GC progression, the function of miR-16-5p in GC cell growth, migration, and apoptosis was examined. The results showed that miR-16-5p mimics suppressed MGC-803 cells the growth and migration and enhanced apoptosis (additional file 1: Fig. S1), whereas miR-16-5p inhibitor enhanced GC cells growth and migration while reducing apoptosis (additional file 2: Fig. S2). According to the above findings, miR-16-5p has the tumor-suppressor effect on GC cells and patients. Rescue experiments on miR-16-5p according to ectopic circPGD level were performed to determine if circPGD can exert a biological function by sponging miR-16-5p. This work also carried out migration, colony-formation, wound healing, proliferation and apoptosis assays. As a result, miR-16-5p mimics decreased circPGD effect on promoting the growth and migration of GC cells while suppressing apoptosis (Fig. 4A–E). After miR-16-5p inhibitor transfection, circPGD siRNA effect could be eliminated partially (Fig. 4I–M). MiR-16-5p mimics abolished related protein levels that were altered by pcicR-circPGD (Fig. 4F–H). Simultaneously, miR-16-5p inhibitor could restore the changes in the protein levels caused by si-circPGD (Fig. 4N–P). In summary, circPGD promotes GC progression by the sponge of miR-16-5p.

### ABL2 serve as miR-16-5p direct target

**Fig. 5:**
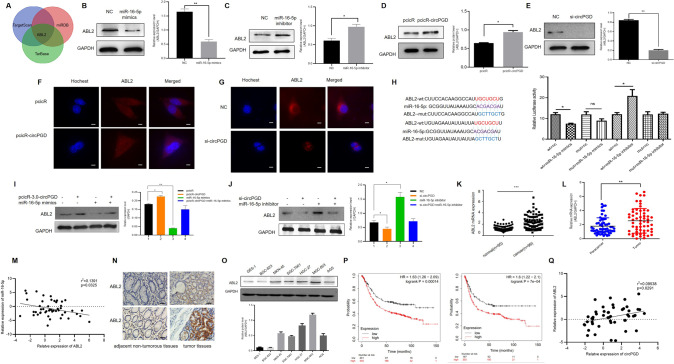
ABL2 directly targets miR-16-5p and its expression increases within GC tissues, which is related to GC development and dismal survival. **A** Public databases predicted that ABL2 may be miR-16-5p downstream protein. **B** and **C** Western blotting revealed that miR-16-5p mimics downregulated the ABL2 expression and miR-16-5p inhibitor upregulated the expression of ABL2. **D** and **F** Western blotting and immunofluorescence revealed that overexpression of circPGD in BGC-823 cells could increased the ABL2 protein level (scale bars = 25 μm). **E** and **G** circPGD silencing within MGC-803 cells decreased the ABL2 protein expression. **H** Luciferase reporter assay showed that there are binding sites in miR-16-5p and ABL2. **I** MiR-16-5p mimics could reduce ABL2 level induced by the circPGD plasmid. **J** MiR-16-5p inhibitor can enhance ABL2 expression, which was inhibited by the circPGD siRNA. **K** ABL2 mRNA expression within healthy samples (*n* = 95) as well as GC samples (*n* = 99) with ONCOMINE. **L** ABL2 mRNA levels within 50 GC tissues as well as 50 corresponding non-tumor samples detected through qRT-PCR revealed that it was upregulated in tumor tissues. **M** ABL2 level showed negative correlation with miR-16-5p level. **N** Immunohistochemistry was carried out for detecting ABL2 levels within GC samples compared with corresponding non-tumorous samples (original magnification, ×200). **O** Detection of the ABL2 expression levels within GES-1 cells as well as six GC cell lines by western blotting. **P** Influence of the ABL2 expression level on OS and PFS in GC cases was examined by the Kaplan–Meier Plotter. **Q** The ABL2 level was positively correlated with circPGD expression. *p < 0.05, **p < 0.01, ***p < 0.001.

According to TargetScan, miRDB, and TarBase databases, ABL2 mRNA possesses miR-16-5p MRE, suggesting the direct targeting relation of miR-16-5p with ABL2 (Fig. 5A). Western blotting showed that miR-16-5p mimics within MGC-803 cells decreased ABL2 level (Fig. 5B). By contrast, miR-16-5p inhibitor increased ABL2 level (Fig. 5C). Western blotting and IF results showed that high circPGD expression could enhance the expression of ABL2 in GC cells (Fig. 5D–G), while miR-16-5p reversed it (Fig. 5I, J). Additionally, this work established luciferase reporter plasmids that contained MUT and WT ABL2 mRNA 3′-UTR. As revealed by luciferase reporter assays miR-16-5p mimics decreased WT luciferase activity significantly in 293T cells, and miR-16-5p inhibitor had opposite effect, but mutant cells showed no such effect (Fig. 5H). Bases on these results, miR-16-5p inhibited ABL2 levels through direct binding to ABL2 mRNA’s 3′-UTR. Furthermore, ABL2 was expressed in both GES-1 cells and GC cells, which was upregulated within most GC cells. ABL2 was most significantly upregulated within MGC-803 cells, whereas downregulated within BGC-823 cells (Fig. 5O).

### ABL2 acts as a tumor-promoting factor in GC

Compared with paracancer tissues, the ABL2 mRNA level increased in GC tissues (Fig. 5L), conforming to ONCOMINE analysis (https://www.oncomine.org) (Fig. 5K). Furthermore, ABL2 mRNA level showed negative relation to miR-16-5p level (Fig. 5M). Immunohistochemistry detected ABL2 expression in 57 GC patients, and found that ABL2 expression increased within cancer samples relative to corresponding non-tumor tissues (Fig. 5N). Additional file 7: Table S1 presents clinicopathological parameters showing the relationship between ABL2 expression and GC cases. A publicly available database, the Kaplan–Meier Plotter (http://www.kmplot.com), suggested the close relation of ABL2 upregulation with dismal progression-free survival (FPS) and overall survival (OS) (Fig. 5P) for GC cases. Additionally, ABL2 and circPGD were positively correlated in GC patients (Fig. 5Q). Overall, ABL2 was upregulated within GC samples, indicating poor progression of GC patients, which was positively correlated with circPGD.

### MiR-16-5p suppresses GC progression by inhibiting ABL2 expression

**Fig. 6:**
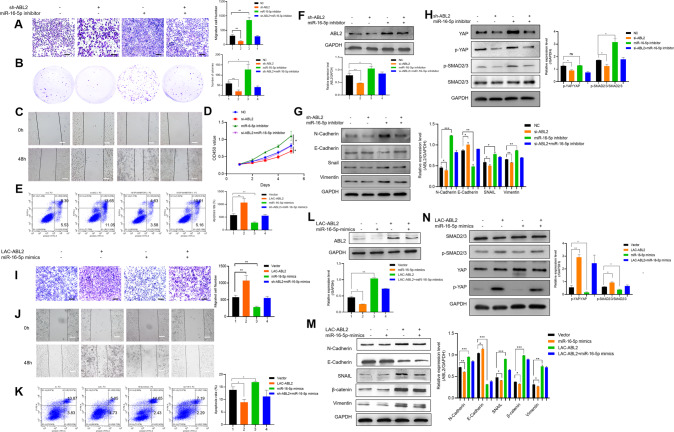
MiR-16-5p suppresses GC progression by inhibiting ABL2 expression. **A** Transwell (scale bars = 100 μm), **B** colony-formation, **C** wound healing (scale bars = 50 μm), **D** CCK-8, and **E** apoptosis assays revealed the effect of miR-16-5p inhibitor on promoting cell migration and growth, while it inhibited apoptosis, and ABL2 siRNA mitigated it. **F** As revealed by Western blotting assay, miR-16-5p inhibitor increased ABL2 expression. **G** and **H** ABL2 silencing within MGC-803 cells decreased N-Cadherin, Vimentin, Snail, YAP, and p-SMAD2/3 levels, while no change was observed for SMAD2/3. **I–K** miR-16-5p upregulation can alleviate GC cells biological behaviors with ABL2 downregulation. **L** MiR-16-5p mimics reduces the role of Arg lentiviral activation particles. **M** and **N** Western blotting detected the effects of miR-16-5p mimics and Arg lentiviral activation particles effects on EMT and SMAD2/3, p-SMAD2/3, YAP, and p-YAP protein levels. *p < 0.05, **p < 0.01, ***p < 0.001.

ABL2 was effectively silenced and overexpressed in GC cells (additional file 3: Fig. S3). ABL2 knockdown reduced the healing ability and the number of migratory MGC-803 cells, and to some degree, decreased proliferation and formed smaller clones than in the control cells (additional file 4: Fig. S4). Consistent with the above results, overexpressed ABL2 promoted GC cell migration and proliferation (additional file 5: Fig. S5). Rescue experiments were conducted to analyze how ABL2 affected miR-16-5p effect on GC development. Functionally, ABL2 abolished miR-16-5p effect on GC cell proliferation, migration, and apoptosis (Fig. 6A–E, I–K). According to Fig. 6F, L, miR-16-5p affected ABL2 expression level. Furthermore, as shown in 6G, H and M, N, the abnormal ABL2 levels within GC cells restored miR-16-5p role in EMT, as well as p-YAP and p-SMAD2/3 proteins. Overall, miR-16-5p suppressed GC progression by inhibiting ABL2 expression.

### Knockdown circPGD and ABL2 decreases GC cell xenograft tumors growth and metastasis in vivo

**Fig. 7:**
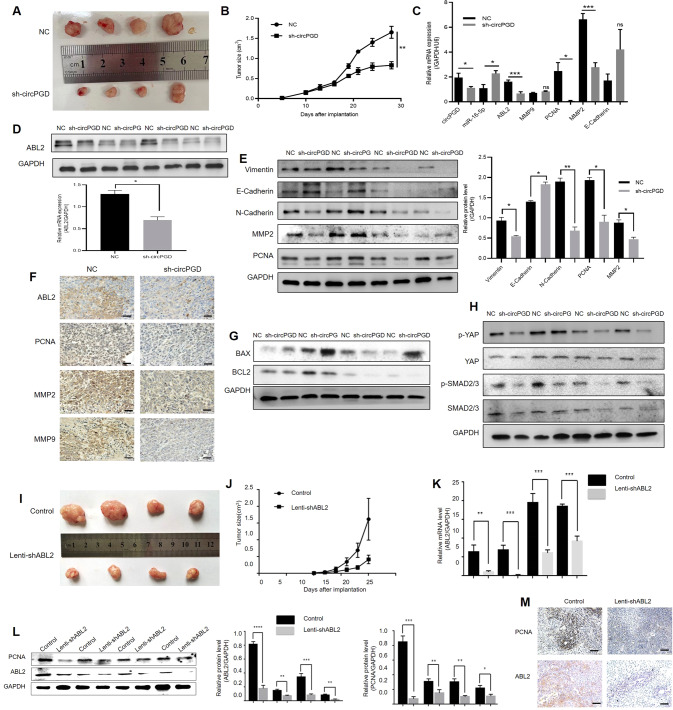
CircPGD and ABL2 promotes GC cell growth in vivo. **A** and **I** Images showing MGC-803 cells induced subcutaneous xenografts. **B** and **J** MGC-803 cells induced tumor volumes measured every 3 days for 6–8 times. **C** qRT-PCR analyzed circPGD, miR-16-5p, ABL2, and PCNA mRNA expression within subcutaneous xenograft tumors. **D** Western blotting analyzed the ABL2 expression level in subcutaneous xenograft tumors. **E** Western blotting detected the levels of Vimentin, E-Cadherin, N-Cadherin, MMP2, and PCNA expression when circPGD was silenced in subcutaneous xenograft tumors. **F** ABL2, PCNA, MMP2, and MMP9 expression was observed within subcutaneous xenograft tumors through immunohistochemistry (scale bars = 100 μm). **G** and **H** Western blotting assay conducted to detect proteins related to apoptosis and signaling pathway. **K** qRT-PCR examined the ABL2 mRNA levels and found that it was low in ABL2-silenced MGC-803 cell subcutaneous xenograft tumors. **L** ABL2 and PCNA levels were decreased in ABL2-silenced subcutaneous xenograft tumors. **M** ABL2 and PCNA expression was analyzed within the subcutaneous xenograft tumors through immunohistochemistry (scale bars = 100 μm). *p < 0.05, **p < 0.01, ***p < 0.001.

CircPGD and ABL2 effects in vivo were examined by injecting MGC-803 cells with sh-circPGD and sh-ABL2 or sh-NC cells into the nude mice. Three-four weeks later, the subcutaneously transplantated tumors were maintained. Compared with sh-NC cells, the sh-circPGD and sh-ABL2 cells showed a reduced tumor weight and volume (Fig. 7A, I). Tumor growth curves with silenced circPGD (Fig. 7B) and ABL2 (Fig. 7J) significantly decreased compared with control of MGC-803 cells. In addition, when circPGD and ABL2 were silenced in MGC-803 cells, ABL2 and PCNA mRNA expression decreased (Fig. 7C, K). Western blotting revealed a decrease in ABL2 and PCNA protein levels (Fig. 7D, E, L). Immunohistochemistry also revealed changes in these proteins (Fig. 7F, M). The knockdown of circPGD decreased EMT process and the levels of apoptotic proteins increased (Fig. 7E, G) via p-SMAD2/3 and p-YAP signaling pathways (Fig. 7H). Overall, the knockdown of circPGD and ABL2 may suppress GC cells growth in vivo.

### CircPGD encodes the 219aa tumor-promoting protein

**Fig. 8:**
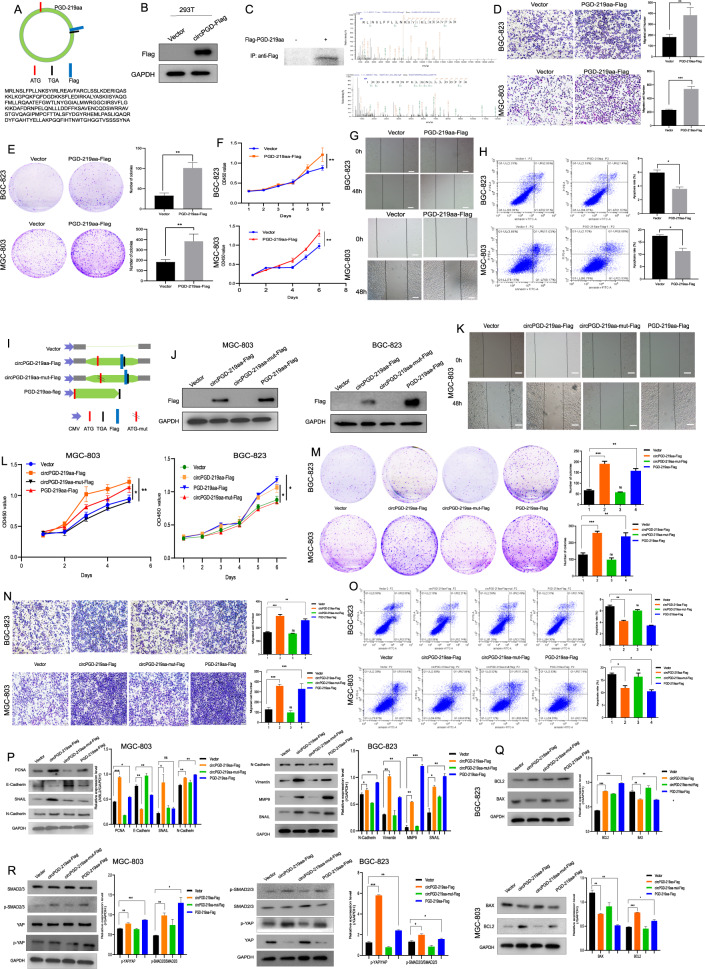
CircPGD encodes a novel PGD-219aa protein and promotes GC cell growth and migration while suppressing their apoptosis. **A** Prediction of open reading frame (ORF) containing the reversed spliced sites in circPGD. **B** Western blotting detected whether circPGD-Flag plasmids expressed Flag proteins in 293T cells. **C** Protein purification in MGC-803 cells subject to transfection with circPGD-219aa-Flag and Co-IP using Flag antibody, followed by validation through western blotting. LC–MS/MS was applied for identifying peptide sequences of collected proteins. The front (upper right) and the end (lower right) amino acid sequences are displayed. **D–H** The function of PGD-219aa-Flag plasmids in GC cells was monitored. **D** Transwell migration, **E** colony-formation, **F** CCK-8, **G** wound healing and **H** apoptosis assays result compared with the control vector in GC cells. **I** Sample maps of circPGD-219aa plasmids. **J** Western blotting detected PGD-219aa expression in GC cell lines transfected with vector, circPGD-219aa-Flag, cricPGD-219aa-mut-Flag, and PGD-219aa plasmids using the Flag antibody. **K** Wound healing, **L** CCK-8, **M** colony-formation, **N** Transwell migration, and **O** apoptosis assays analyzed the effects of empty vector, as well as circPGD-219aa-Flag, cricPGD-219aa-mut-Flag and PGD-219aa plasmids on GC cell migration, proliferation, and apoptosis. **P, Q**, and **R** Western blotting detected whether PGD-219aa affected EMT, as well as apoptosis- and signaling pathway-related proteins.

Bioinformatics analysis indicated that circPGD has an open reading frame (ORF), which may encode a protein, namely PGD-219aa containing a reverse splicing site (Fig. 8A). A circPGD overexpression vector with the Flag tag before the stop codon TGA (circPGD-Flag) was constructed to analyze the protein-encoding ability. Western blotting found that, compared with 293T cells transfected with the empty vector, the Flag antibody showed a visible band in circPGD-Flag cells (Fig. 8B), demonstrating that circPGD can encode proteins. Furthermore, nascent protein was purified by IP by the use of an anti-Flag antibody to validate in MGC-803 cells. According to LC–MS/MS analysis, nascent protein upstream amino acid sequence conformed to that amino acid sequence encoded at the circPGD cyclization site and different from PGD, which confirmed that PGD-219aa was translated from circPGD. The molecular weight was 24.8 kDa (Fig. 8C). The function of circPGD-219aa was analyzed further by constructing the linear form vector of PGD-219aa marked with the Flag tag (PGD-219aa-Flag) while transfecting it to GC cells. According to related analysis, PGD-219aa-Flag plasmid enhanced cell growth and migration, while it suppressed apoptosis (Fig. 8D–H). These results indicate that circPGD encodes a 219aa tumor-promoting protein.

The transfection of the ATG mutant circPGD overexpression vector (circPGD-219aa-mut-Flag) resulted in the lost ability of circPGD-219aa to encode amino acids. Its function was compared with wild-type circPGD-219aa-Flag and the linear form of PGD-219aa-Flag (Fig. 8I). Western blotting detected the Flag tag in PGD-219aa-Flag- and circPGD-219aa-Flag-transfected MGC-803 and BGC-823 cells (Fig. 8J), indicating PGD-219aa protein production. A series of experiments showed that circPGD-219aa-Flag and PGD-219aa-Flag plasmids could promote GC cell growth and migration, and decrease BGC-823 and MGC-803 cell apoptosis. Meanwhile, circPGD-219aa-mut-Flag had no obvious effects in GC cells (Fig. 8K–O). In addition, Western blotting showed that circPGD-219aa promoted EMT and suppressed apoptosis via SMAD2/3 and YAP signaling pathways (Fig. 8P–R). In conclusion, circPGD encodes a novel 219aa protein that promotes the malignant transformation of GC.

## Discussion

CircRNAs represent the new family of RNAs that have the circular loop formed by unique back-splicing biological processes with different molecular mechanisms. Regardless of the significantly decreased back-splicing efficiency compared with linear RNAs [26], circRNAs in cells are abundant because they are stable and have extended half-life period. With continued studies, circRNAs are expected to become increasingly important in preventing, diagnosing, and treating human cancer. The present work examined the new circRNA, circPGD, which promoted tumor development of GC. CircPGD expression increased within GC samples, related to larger tumor size and lymph node metastasis. Functionally, circPGD enhances GC cell growth and metastasis in vitro and in vivo and suppresses apoptosis, suggesting that circPGD is a tumor promoter. The proteins PCNA, MMP2, and MMP9, as well as other proteins associating with EMT and apoptosis, and p-YAP and p-SMAD2/3 showed the corresponding alternation. It suggested that circPGD may mediated the migration, prolifeartion, cell apoptosis and EMT via YAP and SMAD2/3 signialling pathway in GC cells.

Furthermore, circRNAs may regulate biological processes by sponging miRNA sponges, combining with RNA-binding proteins (RBPs), encoding proteins or peptides, and regulating gene transcription. According to previous studies, circRNAs are the miRNA sponges or competing endogenous RNAs (ceRNAs) for regulating gene expression [27, 28]. CiRs-7 can absorb miRNA-7 to regulate the biological behavior of various cells [28–31]. CircNHSL1 enhances GC development via miR-1306-3p/SIX1/Vimentin and miR-149-5p/YWHAZ axis [32, 33]. According to our bioinformatics prediction, circPGD has miR-16-5p MRE, while miR-16-5p expression is negatively associated with circPGD. Subsequently, we found that there are complementary binding in circPGD and miR-16-5p. MiR-16-5p expression decreased within GC samples, which showed negative relation to tumor size, suggesting that miR-16-5p is the possible tumor suppressor. In addition, miR-16-5p reversed circPGD effect on promoting ABL2 level, cell growth and migration, and inhibiting apoptosis, whereas ABL2 abolished miR-16-5p effect on suppressing growth, migration, as well as cell apoptosis. Taken collectively with previous results, circPGD promotes GC progression by sponging miR-16-5p, thereby mitigating the inhibition on ABL2. Although ABL2 has been studied in hepatocellular carcinoma, breast cancer, lung adenocarcinoma, and diabetic nephropathy [19–22], the function and detailed effect of ABL2 on GC remains unclear. The present work revealed ABL2 upregulation within GC samples relative to paracancer samples. The TGF-β signaling pathway has an important effect on modulating cell proliferation, migration, invasion and differentiation within many biological systems [34, 35]. Hippo signaling has been previously suggested to regulate cell growth and apoptosis, as evidenced based on mosaic screens of *Drosophila melanogaster* [36, 37]. According to our results miR-16-5p abolished abnormal circPGD expression influence on EMT, YAP and SMAD2/3 signaling pathway-related proteins, and ABL2 reduces miR-16-5p impact on related proteins, suggesting the role of circPGD, miR-16-5p, and ABL2 in regulating GC development by p-SMAD2/3 and p-YAP signaling pathways.

In addition, some circRNAs such as circDIDO1, circARHGAP35, and circMAPK1 can encode novel contain reverse splicing site proteins, and play a significant role in human cancers [38–40]. We also found that circPGD can encode a novel 219aa protein, which containing the reverse splicing nucleotide. It promotes migration and proliferation, while inhibits BGC-823 and MGC-803 cell apoptosis. Furthermore, PGD-219aa could affected EMT, apoptosis, and signaling pathway-related proteins in GC cells.

Collectively, this work reports one new circRNA that play a tumor-promoting role in GC. CircPGD promotes GC cell proliferation and metastasis and inhibits apoptosis through targeting the miR-16-5p/ABL2 axis while encoding the PGD-219aa protein (additional file 6: Fig. S6). These findings highlight circPGD effect, which serves as the diagnostic biomarker of GC.

## Materials and methods

### Tissue specimens

Informed consent was provided by the participants. Our study protocols gained approval from Ethics Committee of the School of Medicine, Jiangsu University. Fresh tumor tissues as well as paired paracancer tissues from 50 GC patients were collected after surgery for RNA extraction. In addition, tissue samples from 57 patients diagnosed with GC were used for immunohistochemistry. The GC samples were harvested in cases enrolled from the affiliated hospital of Jiangsu University. All participants were selected randomly, and no participant received chemotherapy or radiotherapy before surgery.

### Cell culture

This work obtained healthy gastric mucosal epithelial cells (GES-1) together with six GC cell lines (BGC-823, HGC-27, MKN-45, SGC-7901, MGC-803, and AGS) in Cell Bank of the Chinese Academy of Sciences (Shanghai, China) and American Type Culture Collection (ATCC, Manassas, VA, USA), and stored in liquid nitrogen at the School of Medicine, Jiangsu University. AGS cell line was cultivated within DMEM/F12 (Gibco, Grand Island, NY, USA) containing 1% penicillin–streptomycin as well as 10% fetal bovine serum (FBS, Gibco). This work cultivated the other cells and 293T cells within RPMI1640 (Gibco) in the humid incubators under 37°C and 5% CO_2_ conditions.

### Fluorescence in situ hybridization analysis (FISH) assay

Gene Pharma (Suzhou, China) was responsible for designing and synthesizing Cy3-conjugated circPGD as well as FAM-conjugated miR-16-5p probes. The RNA-FISH kit was purchased from Gene Pharma. After being inoculated onto glass coverslips, cells were cultured overnight, followed by subsequent fixation within 4% paraformaldehyde under ambient temperature for a 30-min period. After 15-min treatment using 0.1% Triton X-100, cells were rinsed by PBS twice, followed by the addition of 200 μL of 1× sealing solution to each well and incubation for 30 min at 37 °C. The solution was decarded and 200 μL of 2× buffer C was added, and incubated under 37 °C for a 30-min period. Probes were diluted to 1 μM, followed by 10-min denaturation in the 75 °C water bath, followed by the addition of 2 μL of 1 μM biotin-probe, 4 μL of 1 μM SA-Cy3/FAM, and 14 μL of PBS, incubation for 30 min at 37 °C, and the addition of 180 μL of buffer E. The cells were incubated with the probe mixture, and hybridization was carried out for 12–16 h at 37 °C. On the following day, the cells were washed with 0.1% buffer F for 10 min at 37 °C, three time with 2× buffer C for 10 min at 60 °C, and three times (10 min each) washed with 2× buffer C under 42 °C. Lastly, 200 μL of diluted DAPI was added to stain cells for 15 min in the dark, washed, sealed, followed by observation with the fluorescence microscope (Leica, Mannheim, Germany).

### Oligonucleotides and plasmid transfection

Gene Pharma (Suzhou, China) was responsible for preparing siRNAs targeting circPGD, miR-16-5p mimics and inhibitor, as well as corresponding negative controls (NCs). The full length of circPGD was cloned into the pcicR-3.0. Asia-Vector Biotechnology (Shanghai, China) was responsible for constructing sh-ABL2. ABL2 was overexpressed by Arg Lentiviral Activation Particles (sc-417779-LAC) (Santa Cruz Biotechnology, Santa Cruz, CA, USA). Cell transfection was conducted following specific protocols.

### RNA isolation and RT-PCR assay

Total tissue and cellular RNA were extracted with TRIzol Reagent (Vazyme, Nanjing, China). PARIS^TM^ Kit (ThermoFisher Scientific, Waltham, MA, USA) was utilized to obtain cytoplasmic and nuclear RNA fractions in line with specific protocols. Total RNA (approximately 10 μg) was subject to incubation using RNase R (40 U, Epicentre Technologies, Madison, USA). HiScript QRT SuperMix from qPCR Kit (Vazyme, Nanjing, China) was thereafter adopted for reverse transcription of RNA. TransStart Top Green qPCR Super Mix (TRAN, China) was utilized for qRT-PCR amplification using the ABI Step One Plus Real-Time PCR System (Applied Biosystems, Foster City, CA, USA). The 2^−ΔΔCT^ method was later adopted for data quantification, with GAPDH being the endogenous reference.

### Transwell migration and wound healing assays

This assay adopted CoStar Transwell chambers (pore size, 8 µm; Corning, Costar, NY, USA). Briefly, upper chamber was added with 2 × 10^4^–1 × 10^5^ cells in serum-free medium (300 μL), while medium containing 10% FBS (600 µL) was added into bottom chamber to induce cell migration. After culture for 12–24 h, cells migrating onto bottom membrane surface were subject to 4% paraformaldehyde fixation, followed by crystal violet staining. For the wound healing assay, the cells were inoculated into the 6-well plate, followed by transfection and culture. Subsequently, a line was created using the 10-µL pipette tip, followed by PBS washing. FBS-free medium was poured for cell culture, followed by cell photographing and counting with the microscope.

### Colony-formation and cell proliferation assay

After transfection, approximately 1 × 10^3^ cells were inoculated to the six-well plate for further culture, with medium exchange at 3-day intervals. Afterward, cells were incubated for 10–14 days, followed by fixation using 4% paraformaldehyde as well as crystal violet staining. Cells (1 × 10^3^) were later inoculated into the 96-well plates for further culture. Cell proliferation was analyzed by Cell Counting Kit-8 (CCK-8) (Tongren, Shanghai, China). The microplate reader was thereby adopted for detecting absorbance value at 450 nm at different times.

### Flow cytometry

An apoptosis kit was purchased from Vazyme. The harvested cells were rinsed by pre-chilled PBS, followed by resuspension with 1× binding buffer (500 µL). Subsequently, the suspension was added with annexin V-fluorescein isothiocyanate solution (FITC, 5 μL) containing PI (5 μL). Meanwhile, BD FACS Calibur flow cytometer (Becton-Dickinson, Franklin Lakes, NJ, USA) was utilized for cell analysis. Each assay was conducted in triplicate.

### Luciferase reporter assay

GenePharma Co. (Suzhou, China) was responsible for preparing luciferase reporter plasmid pmirGLO-luc2 that contained the circPGD WT and MUT sequences, as well as the ABL2 WT and MUT sequences. Thereafter, Lipofectamine™ 3000 reagent was utilized to co-transfect cells with luciferase reporter plasmids and miR-16-5p mimics or inhibitor. After 24–36 h of incubation, this work detected renilla and firefly luciferase activities, and their ratio was determined to examine if there were binding sites in circPGD with miR-16-5p, as well as in miR-16-5p with ABL2. Three independent experiments were performed.

### Western blotting assay

Cells and tissues were subject to lysis within the RIPA buffer (Beyotime Biotechnology, Shanghai, China) that contained phosphatase inhibitors as well as phenylmethanesulfonyl fluoride. Proteins were loaded into 10% SDS-PAGE gels, followed by transfer onto PVDF membranes. Thereafter, 5% defatted milk was added to block the membranes, followed by overnight incubation using primary antibodies (additional file 8: Table S2) under 4 °C as well as 1-h incubation using secondary antibodies under ambient temperature. The enhanced chemiluminescence (ECL) system (Image Quant LAS 4000 mini, Pittsburgh, PA, USA) was adopted for membrane analysis.

### Immunohistochemistry (IHC) assay

This work obtained the IHC kit in Boster Bioengineering (Wuhan, China). Tissue blocks were sectioned at 4μm, and the sections were subject to xylene dewaxing as well as ethanol rehydration. Sections were exposed to 3% hydrogen peroxide for 10 min to suppress endogenous peroxidase activity, and the sections were blocked with 5% BSA, followed by overnight incubation using primary antibodies under 4 °C. On day 2, IgG-biotin and SABC were utilized to incubate sections, followed by visualization using 3,3-diaminobenzidine as well as hematoxylin counter-staining and observation with the microscope.

### Immunofluorescence assay

After being plated onto the glass coverslips, cells were subject to further culture. After fixation with 4% paraformaldehyde, cells were subject to permeabilization using 0.5% Triton X-100 (Sigma-Aldrich) as well as blocking using 5% BSA. After overnight incubation using primary antibodies under 4 °C, the slides were washed by Cy3-labeled Goat Anti-Rabbit IgG (H + L) (Beyotime Biotechnology) on day 2. At last, the slides were incubated using 1 µg/ml Hoechst 33258 (Sigma-Aldrich), followed by mounting using the antifade mounting medium (Beyotime Biotechnology). Later, the confocal laser scanning microscope was employed for cell visualization.

### Mouse xenograft model

This work obtained the four-week-old male nude mice in Model Animal Research Center of Nanjing University. The experimental was carried out in the specific pathogen-free environment in Laboratory Animal Center of Jiangsu University. Each experiment was carried out in line with specific protocols as well as internal biosafety and bioethics guidelines from Jiangsu University and the Jiangsu Municipal Science and Technology Commission (SYXK(su)2018-0053). Approximately 2×10^6^ MGC-803 cells (negative control, sh-circPGD, and sh-ABL2 cells; n = 4/group) per mouse were subject to resuspension within sterile PBS before injection to nude mouse back. After three-four weeks, each mouse was performed cervical dislocation, followed by collection of tumor tissues for further analysis. For metastasis studies, each nude mouse was given injection of approximately 2 × 10^6^ BGC-823 cells with stable GFP-labeled vector transfection via the tail vein (*n* = 4/group). Under anesthesia with 2.5% isofluorane, the mice were viewed with the IVIS Lumina XR III in vivo imaging system (PerkinElmer, Waltham, CA, USA) and the metastatic status was analyzed after 4 weeks.

### LC–MS/MS

MGC-803 cells transfected with empty vector and circPGD-219aa plasmids for 48 h were lysed, and total proteins were incubated with the Flag antibody. The Capturem™ IP & Co-IP Kit (TaKaRa Bio., Tokyo, Japan) was used to obtain the proteins for western blotting and liquid-mass spectrometry (LC–MS/MS) (Sangong Biotech, Wuhan, China) to detected the presence of the PGD-219aa protein.

### Statistical analysis

Results were expressed in a form of the means ± SD. Each experiment was performed in triplicate. GraphPad Prism 7.0 was employed for statistical analysis. Student’s *t*-test or one-way ANOVA was adopted for significant differences among groups. Correlations were examined by Pearson’s correlation analysis. A *p* < 0.05 stood for statistical significance.

## Supplementary information

Additional file 1- Figure S1

Additional file 2- Figure S2

Additional file 3- Figure S3

Additional file 4- Figure S4

Additional file 5- Figure S5

Additional file 6-Figure S6

Additional file 7-Table S1

Additional file 8-Table S2

Additional file legends

Full uncut western blots

## Data Availability

The corresponding author will provide the original data used to support the findings of this study upon reasonable request.
