# Supplementary figures and images for: Circular RNA circPGD contributes to gastric cancer progression via the sponging miR-16-5p/ABL2 axis and encodes a novel PGD-219aa protein
Source: Cell Death Discov. 2022 Sep 14;8:384. doi: 10.1038/s41420-022-01177-0 (PMC9472197; doi:10.1038/s41420-022-01177-0)

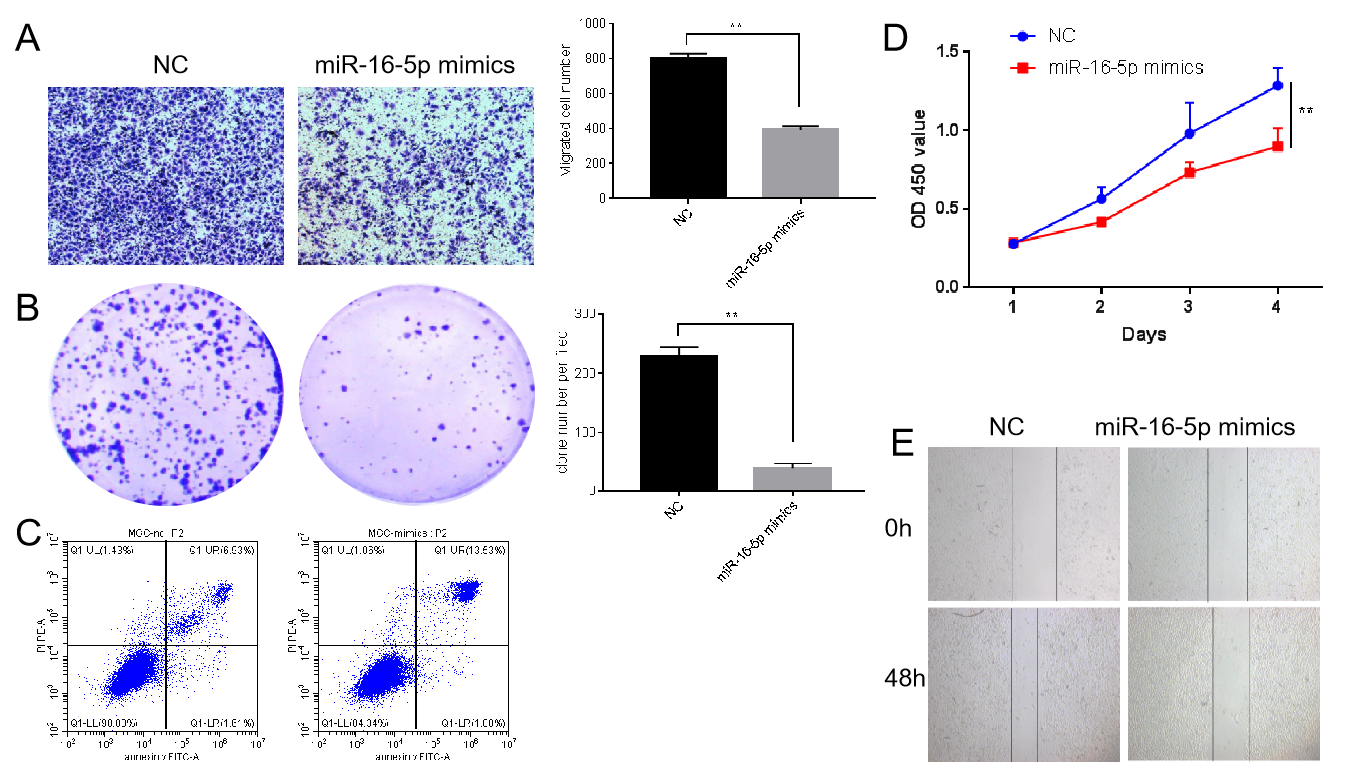

Supplement: Supplementary file 1 — Additional file 1- Figure S1 [file 41420_2022_1177_MOESM1_ESM.tif]

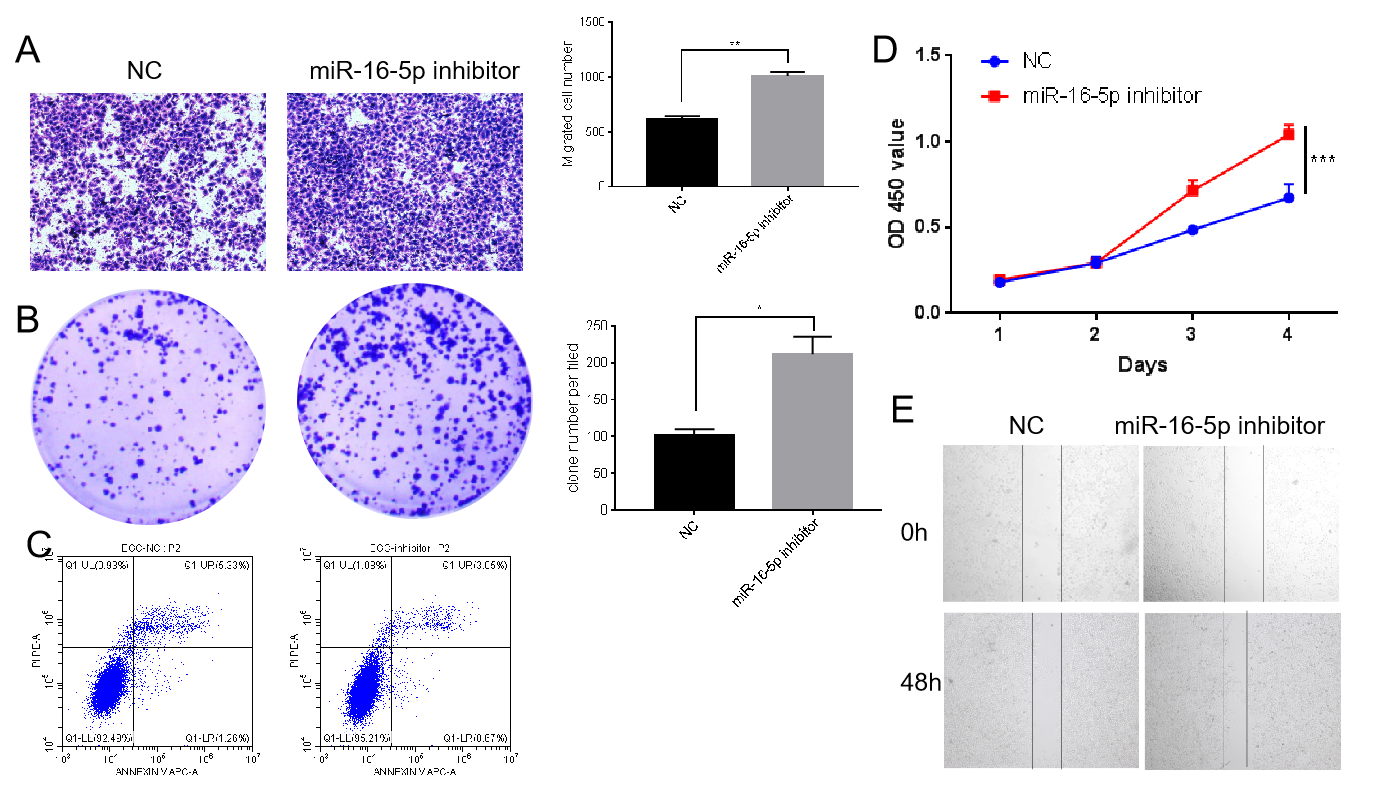

Supplement: Supplementary file 2 — Additional file 2- Figure S2 [file 41420_2022_1177_MOESM2_ESM.tif]

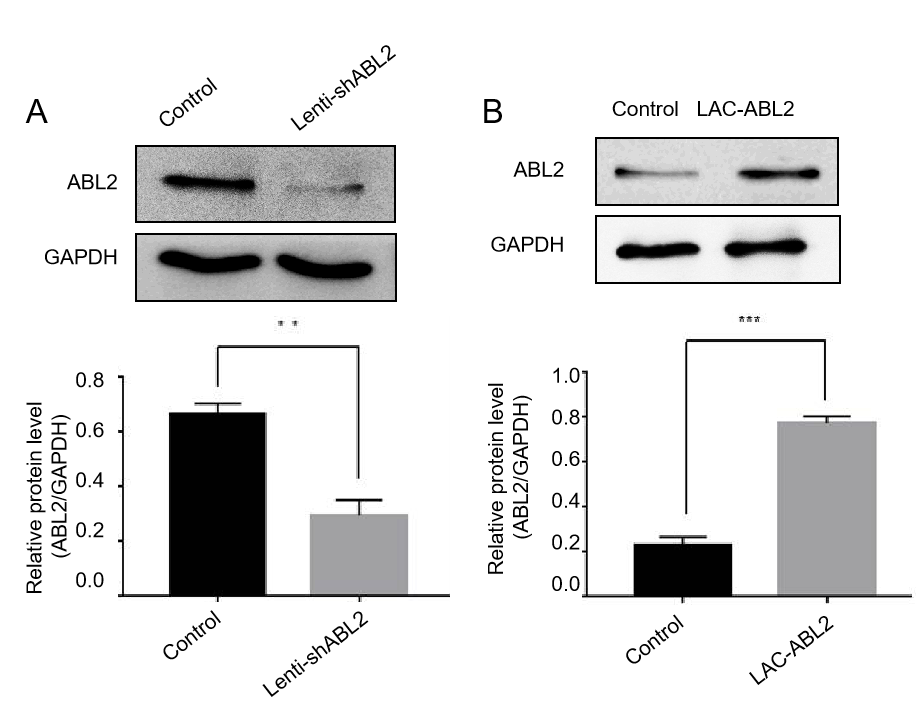

Supplement: Supplementary file 3 — Additional file 3- Figure S3 [file 41420_2022_1177_MOESM3_ESM.tif]

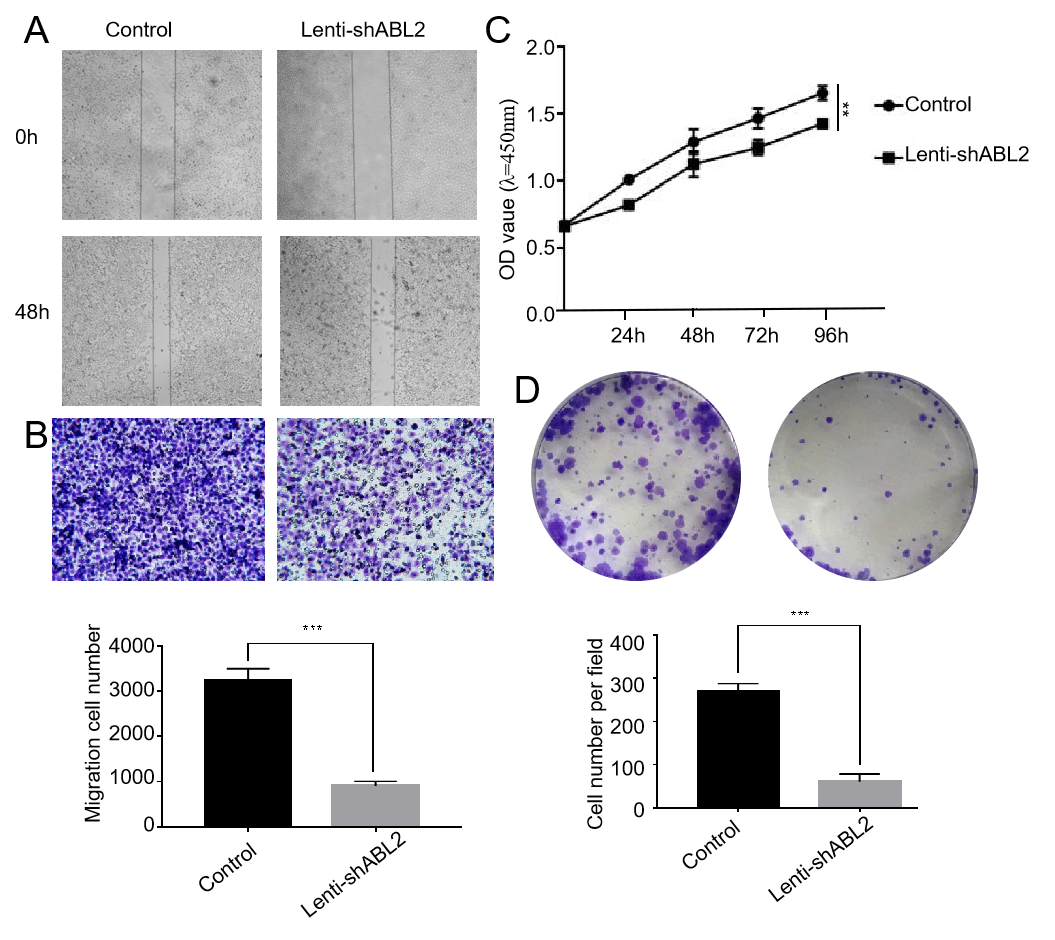

Supplement: Supplementary file 4 — Additional file 4- Figure S4 [file 41420_2022_1177_MOESM4_ESM.tif]

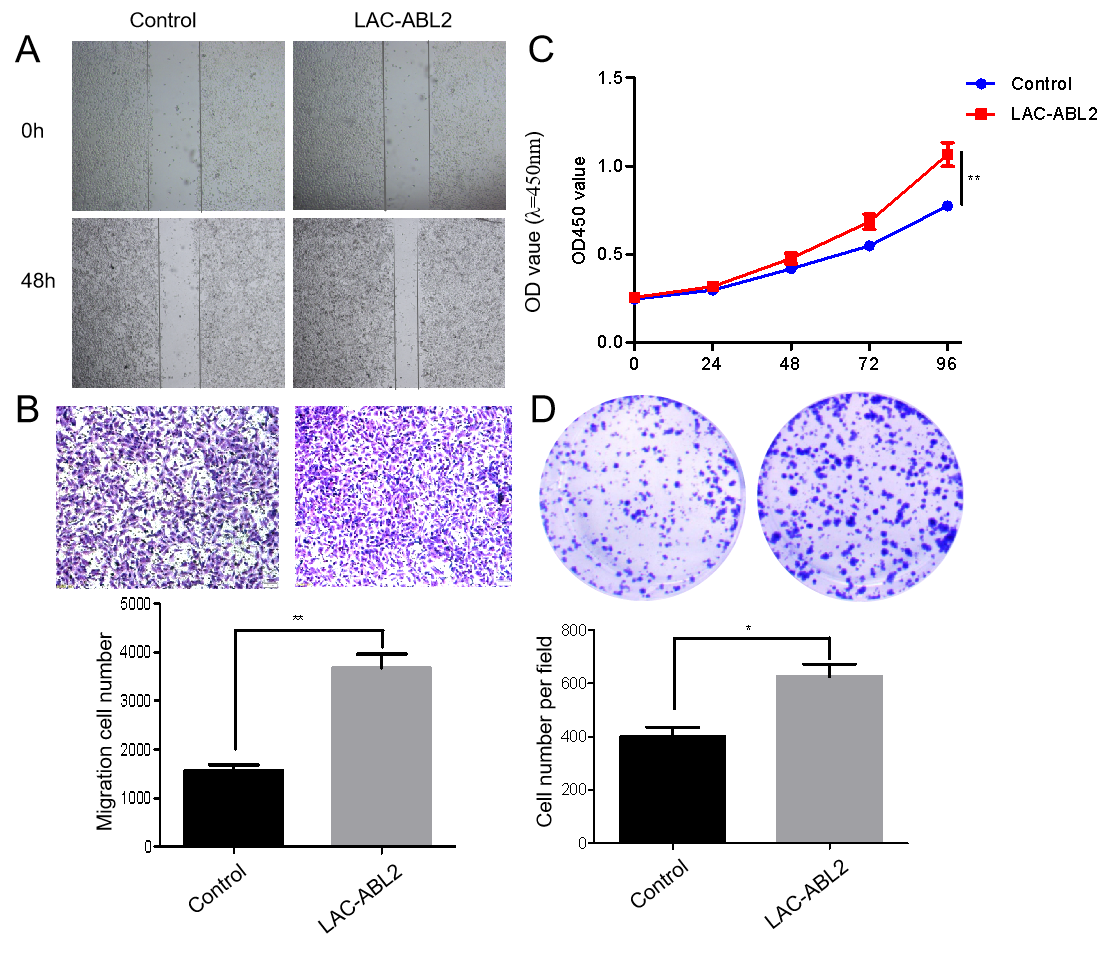

Supplement: Supplementary file 5 — Additional file 5- Figure S5 [file 41420_2022_1177_MOESM5_ESM.tif]

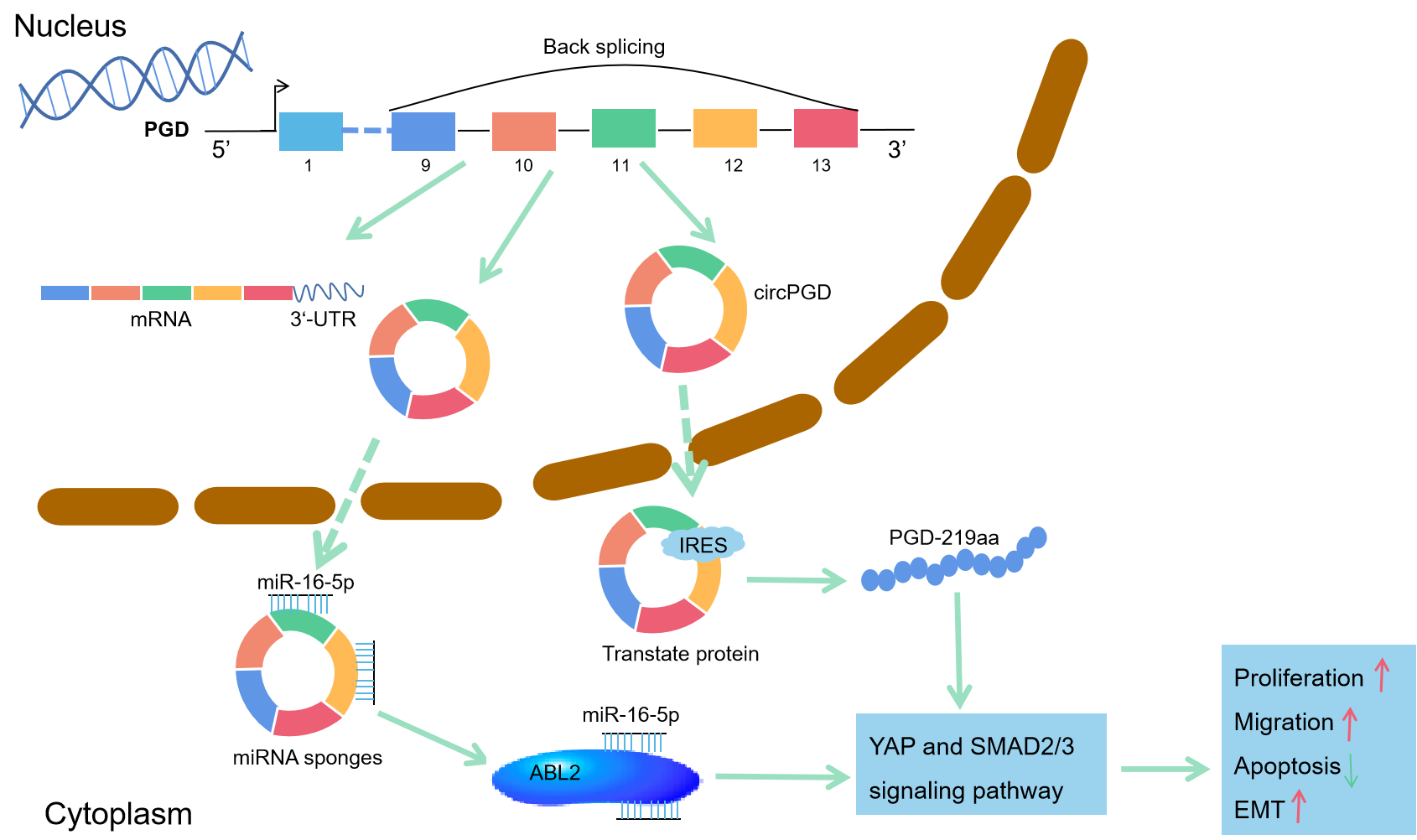

Supplement: Supplementary file 6 — Additional file 6-Figure S6 [file 41420_2022_1177_MOESM6_ESM.tif]
