# Supplementary material for: Circular RNA circPGD contributes to gastric cancer progression via the sponging miR-16-5p/ABL2 axis and encodes a novel PGD-219aa protein
Source: Cell Death Discov. 2022 Sep 14;8:384. doi: 10.1038/s41420-022-01177-0 (PMC9472197; doi:10.1038/s41420-022-01177-0)
Supplement: Supplementary file 9 — Additional file legends [file 41420_2022_1177_MOESM9_ESM.doc]

**Additional file 1: Figure S1**

**Fig. S1** MiR-16-5p mimics inhibited the ability of metastasis and proliferation in MGC-803 cells. **A** Compared with negative control, the number of trans-membrane cells decreased when MGC-803 cell transfected with miR-16-5p mimics (scale bars=100 μm). **B** Colony-formation showed MGC-803 cell transfected with miR-16-5p mimics formed smaller and fewer colonies of cells. **C** miR-16-5p promotes cell apoptosis in MGC-803 cells. **D** CCK-8 assay found that miR-16-5p mimics weakens the ability of cells to grow. **E** wound healing assay showed that miR-16-5p mimics reduced the healing ability of MGC-803 cells (scale bars =100 μm); **p<0.01.

**Additional file 2: Figure S2**

**Fig. S2** MiR-16-5p inhibitor enhanced GC proliferation and metastasis in BGC-823 cells. **A** and **E** BGC-823 cells transfected with miR-16-5p inhibitor enhanced the ability of cell metastasis. **B** and **D** Colony-formation and CCK-8 assay suggested that miR-16-5p inhibitor promoted cell proliferation in BGC-803 cells. **c** miR-16-5p inhibitor suppressed cell apoptosis in BGC-823 cells. Transwell migration (scale bars = 100 μm); wounding healing assay (scale bars =100 μm); *p<0.05, **p<0.01; ***p<0.001.

**Additional file 3: Figure S3**

**Fig. S3** Knockdown and overexpression efficiency of ABL2 in GC cells. **A** The expression of ABL2 in MGC-803 cells was knocked down by transfecting with Lenti-shABL2. **B** Arg Lentiviral Activation Particles were used to overexpress ABL2 in BGC-823 cells. **p<0.01,***p<0.001 versus MGC-803 and BGC-823 cells transfected with Control Particles.

**Additional file 4: Figure S4**

**Fig. S4** Knock-down ABL2 in MGC-803 cells suppressed GC cell proliferation and metastasis. **A** wound healing assay found that knock down ABL2 caused poor healing ability (scale bars =100 μm). **B** transwell migration assay suggested low-expression of ABL2 reduced the migration ability of MGC-803 cells (scale bars = 100 μm). **C** CCK-8 and **D** colony-formation assay found that low expression of ABL2 could inhibit cell proliferation ability in MGC-803 cells. **p<0.01, ***p<0.001.

**Additional file 5: Figure S5**

**Fig. S5** Overexpression of ABL2 in BGC-823 cells promoted the proliferation and metastasis of GC cells. **A** wound healing assay found that high expression of ABL2 in BGC-823 cells promoted cell healing ability (scale bars =100 μm). **B** transwell migration assay suggested high ABL2 level increased the migration ability of MGC-803 cells (scale bars =100 μm). **c** CCK-8 and **d** colony-formation assay found that high expression of ABL2 could enhance cell proliferation ability in BGC-823 cells. *p<0.05, **p<0.01.

**Additional file 6: Figure S6**

**Fig. S6** Molecular mechanism of circPGD in GC progression.

**Additional file 7: Table S1**

Table S1: The correlation of ABL2 expression level to cinicopathological characteristics of gastric cancer

**Additional file 8: Table S2**

Table S2: Antibodies used in the article
