# Supplementary material for: Circular RNA circPGD contributes to gastric cancer progression via the sponging miR-16-5p/ABL2 axis and encodes a novel PGD-219aa protein
Source: Cell Death Discov. 2022 Sep 14;8:384. doi: 10.1038/s41420-022-01177-0 (PMC9472197; doi:10.1038/s41420-022-01177-0)

Dear Editors,
Thank you very much for your information. Below are the original gel of western blots. Due to these western blots were obtained within three years, we cropped the blots at that time to cut cost. But we can guarantee the shown blots are correct, and do not affect the conclusion.

Best Regards
Yours Sincerely
Shihe Shao

Fig. 2H

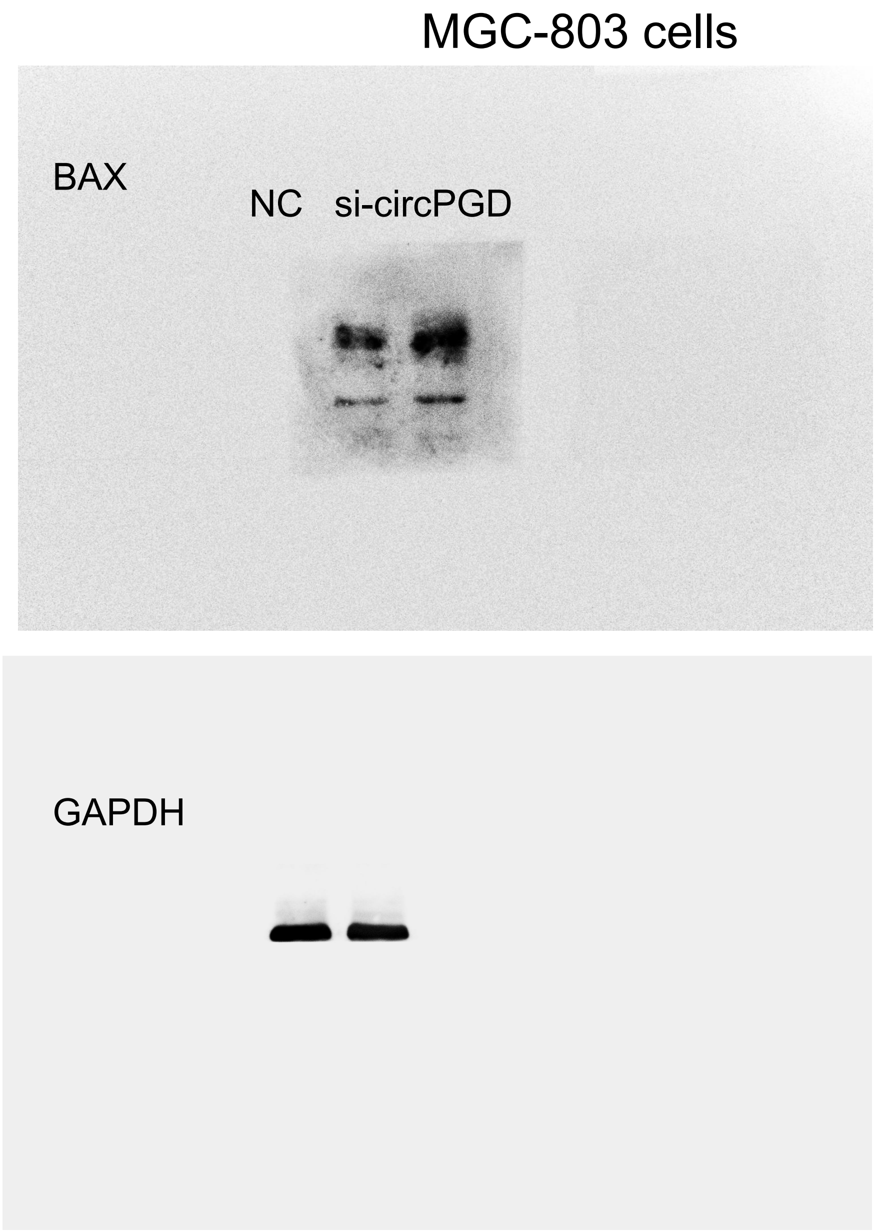

Fig. 2H BGC-823 cells

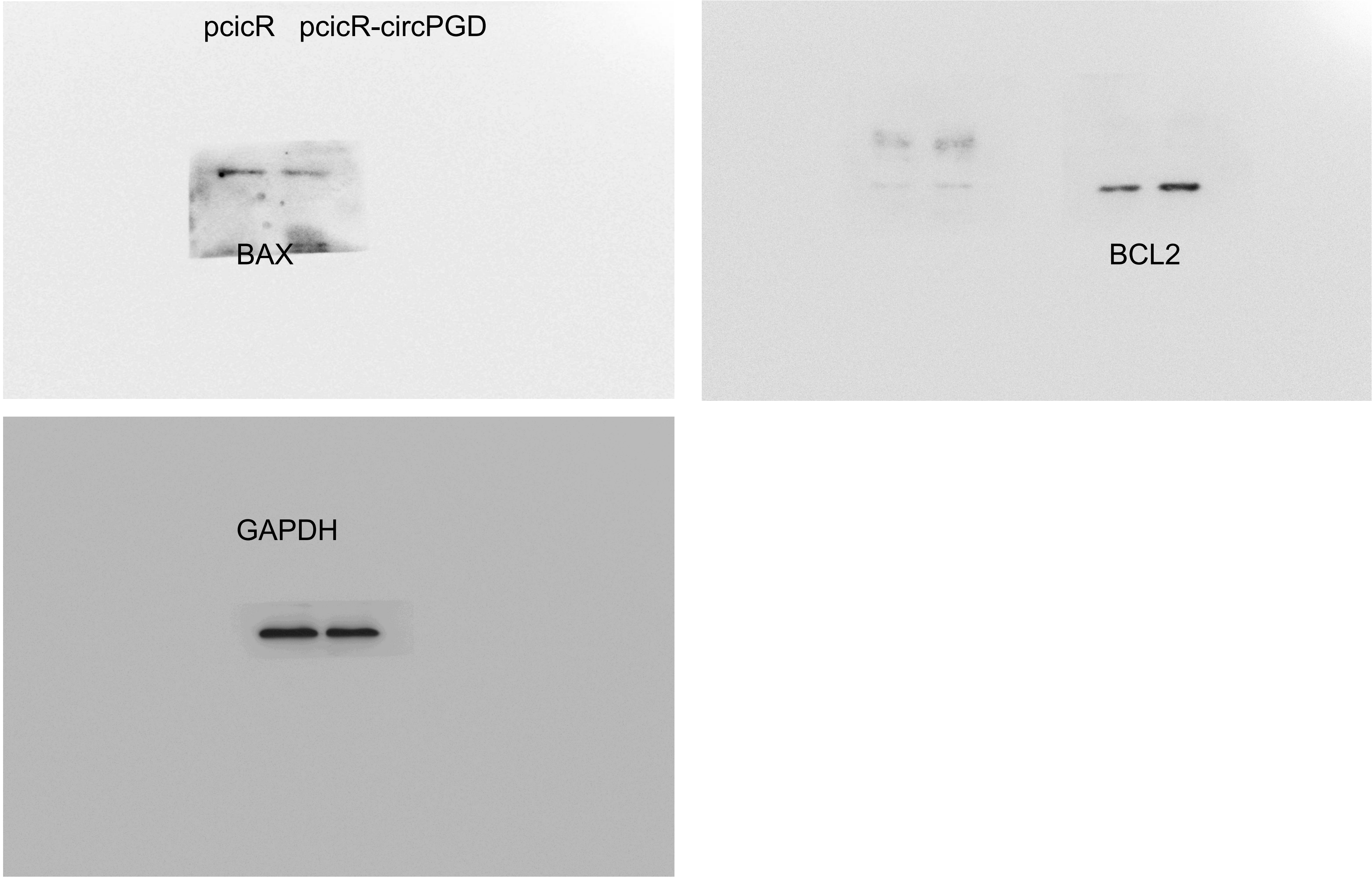

Fig. 2I MGC-803 cells

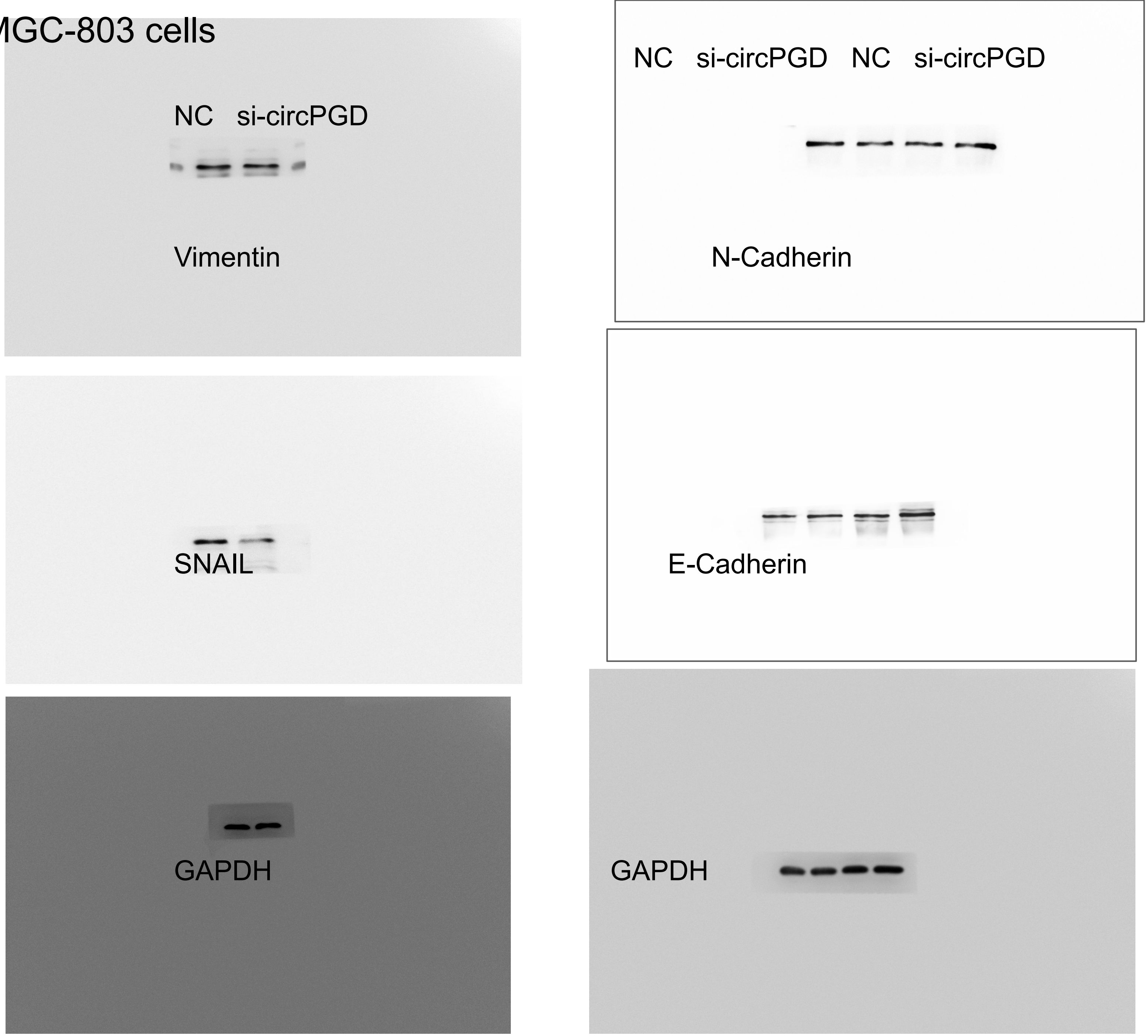

Fig. 2I BGC-823 cells

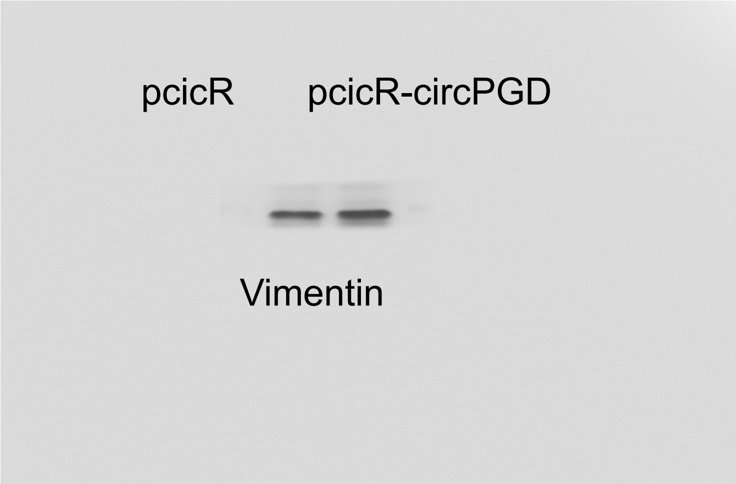

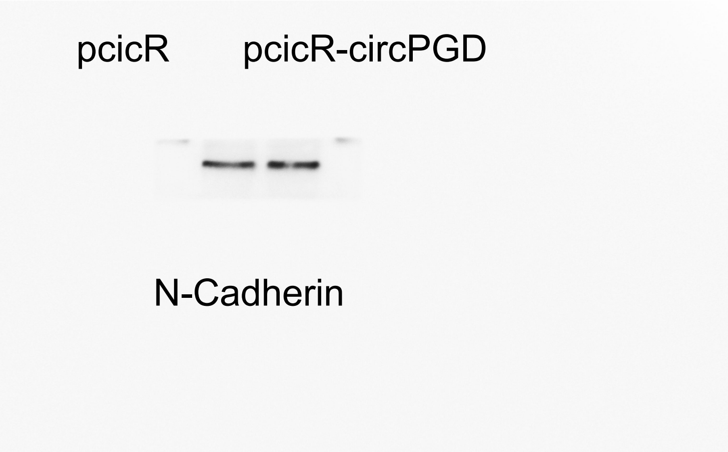

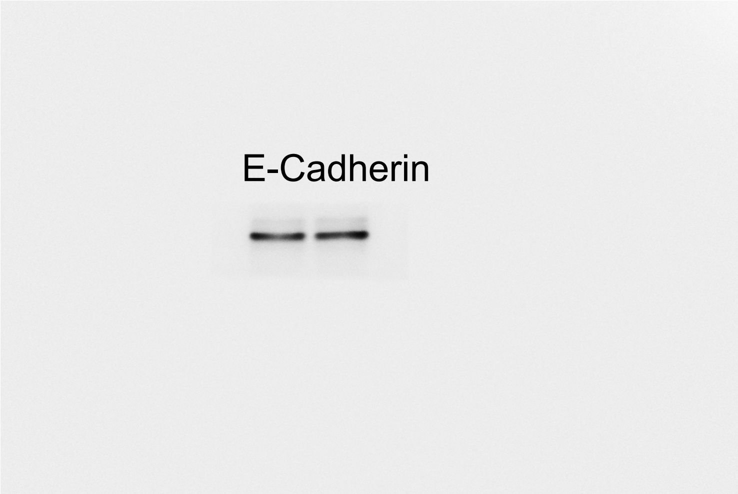

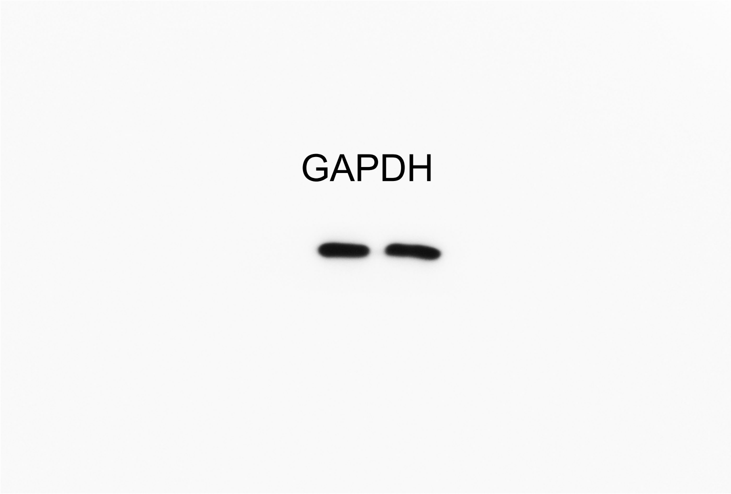

Fig. 2J MGC-803 cells

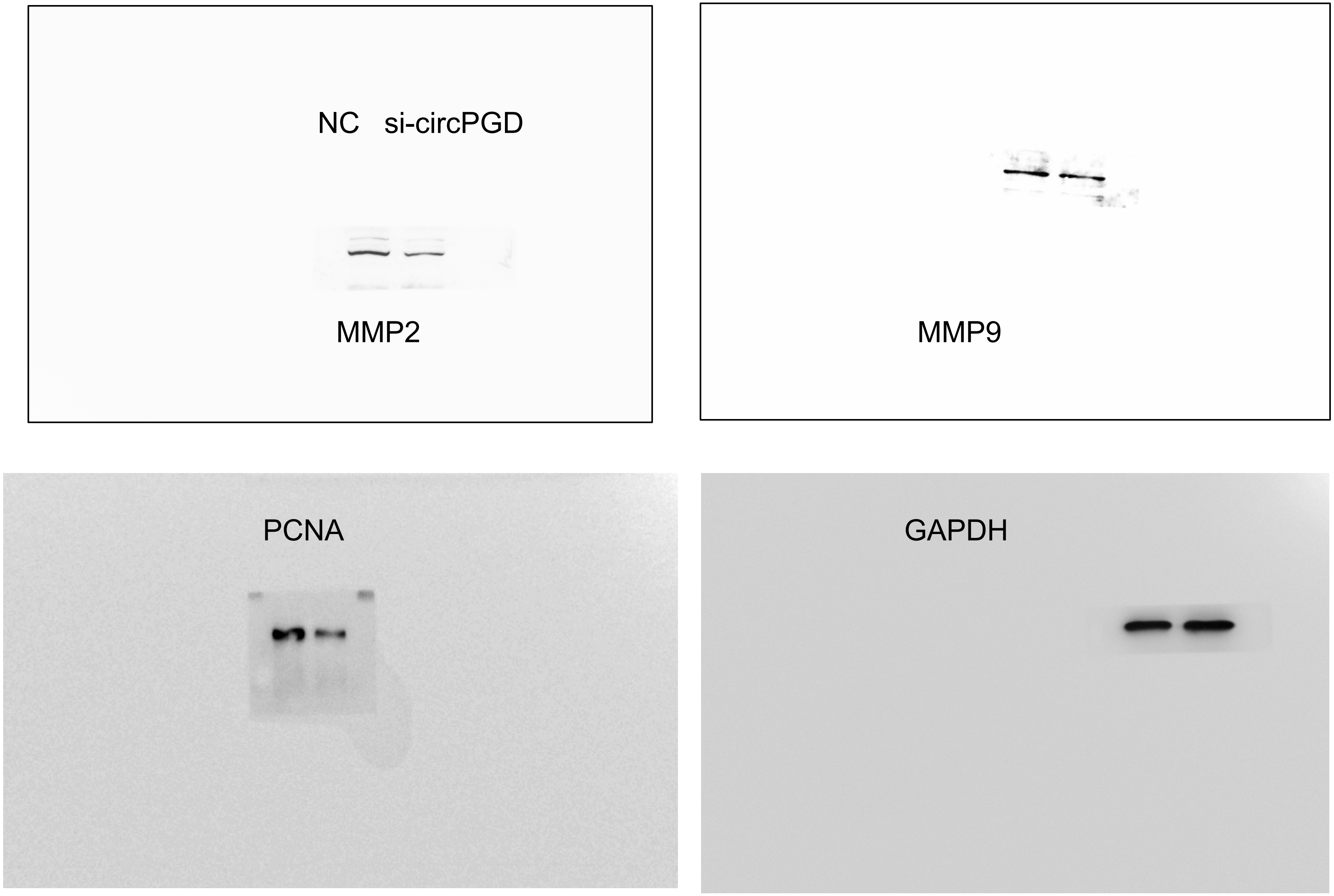

Fig. 2J BGC-823 cells

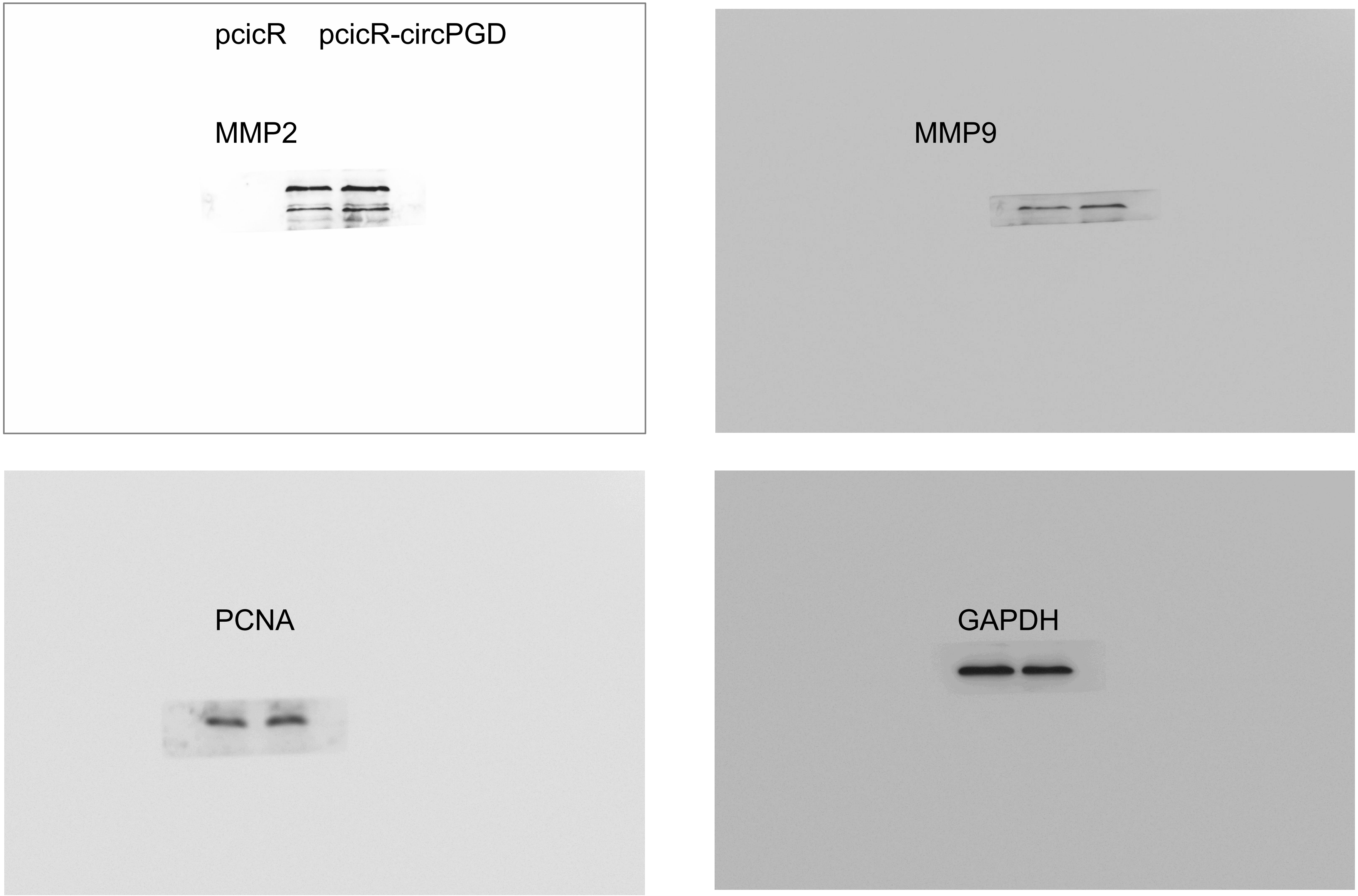

Fig. 2K MGC-803 cells

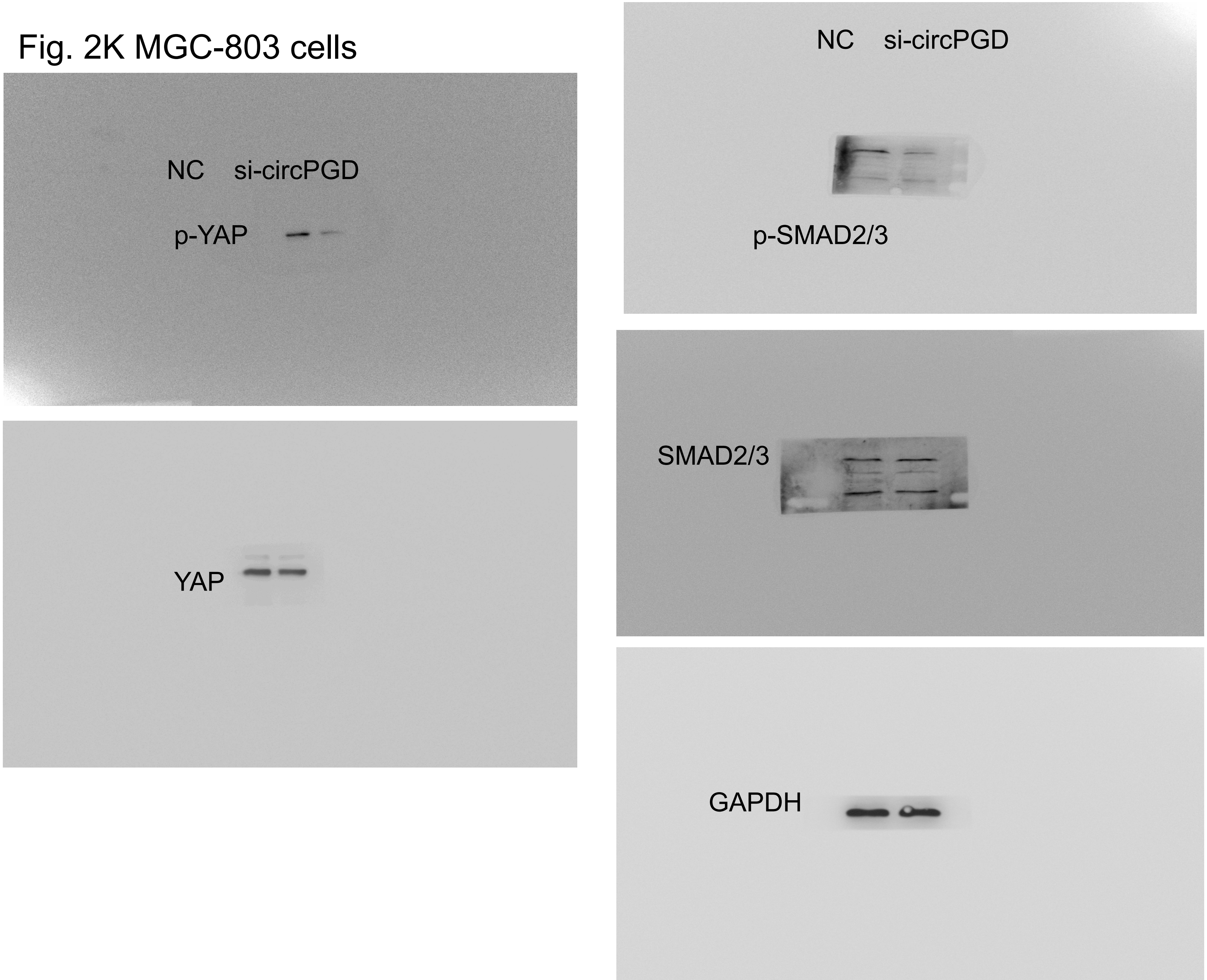

Fig. 2K BGC-823 cells

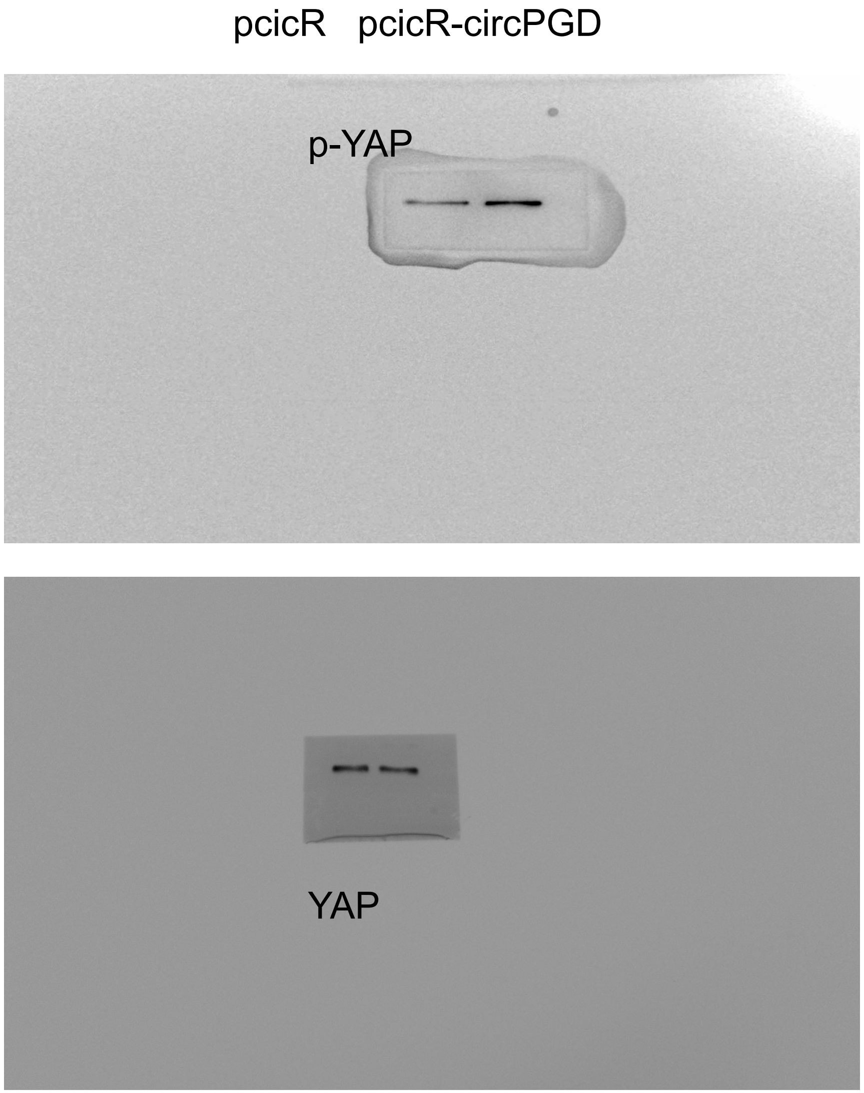

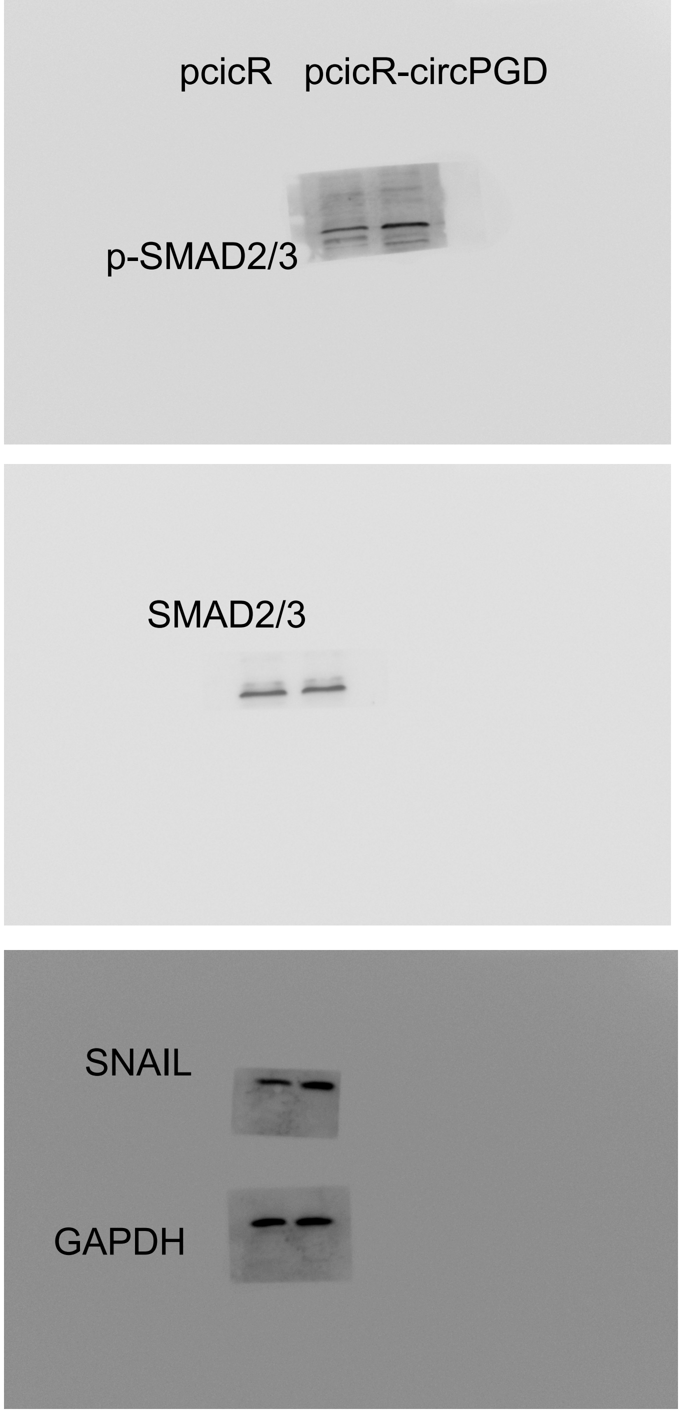

Fig. 4F

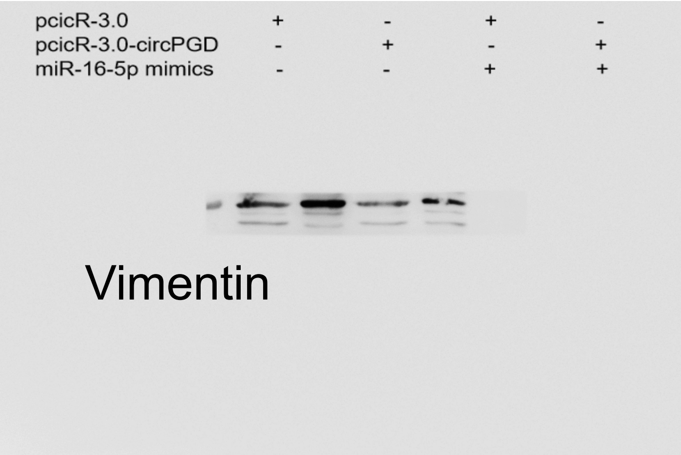
pcicR-3.0 + - + -
pcicR-3.0-circPGD - + - +
miR-16-5p mimics - - + +
Vimentin

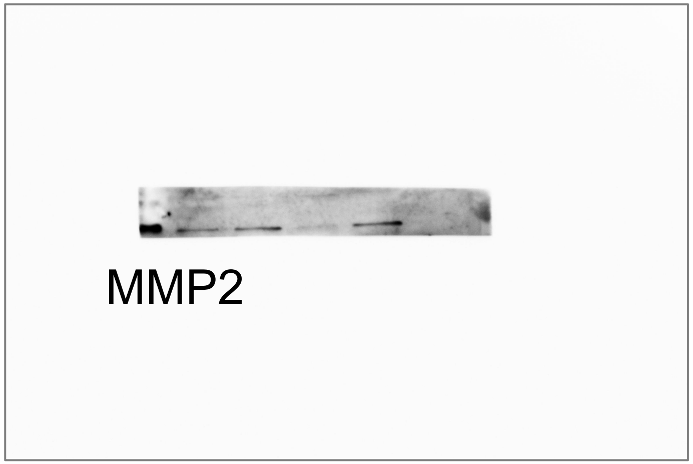
MMP2

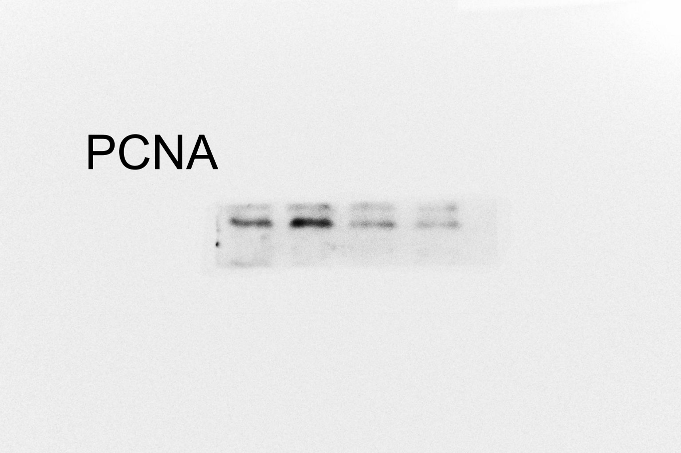
PCNA

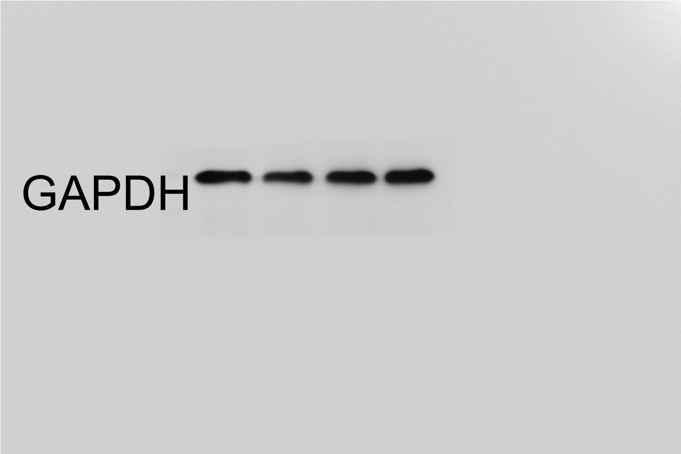
GAPDH

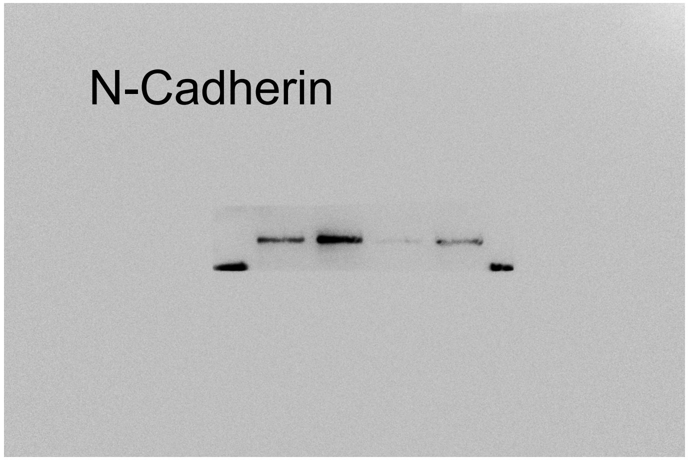
N-Cadherin

Fig. 4G

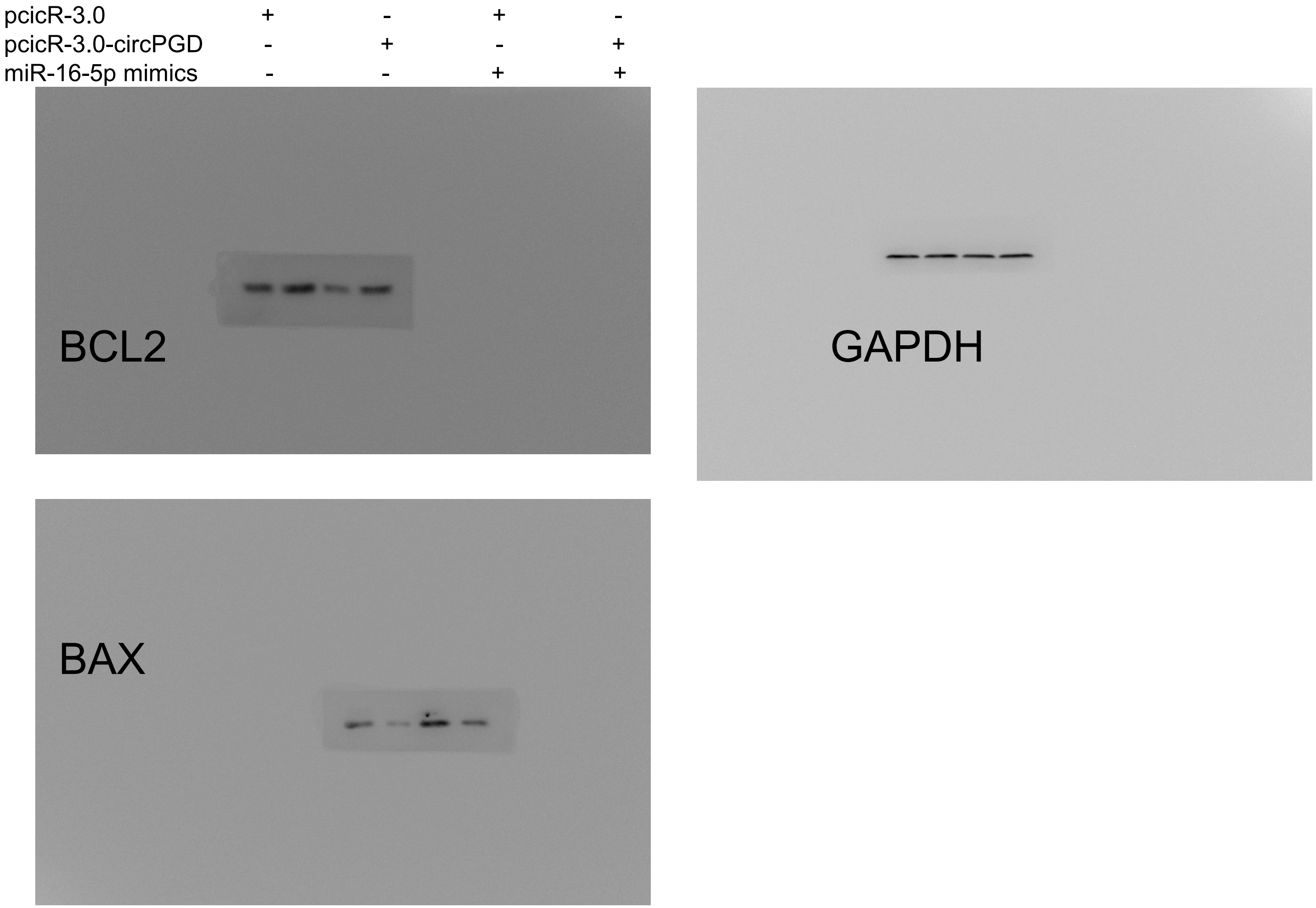

Fig. 4H

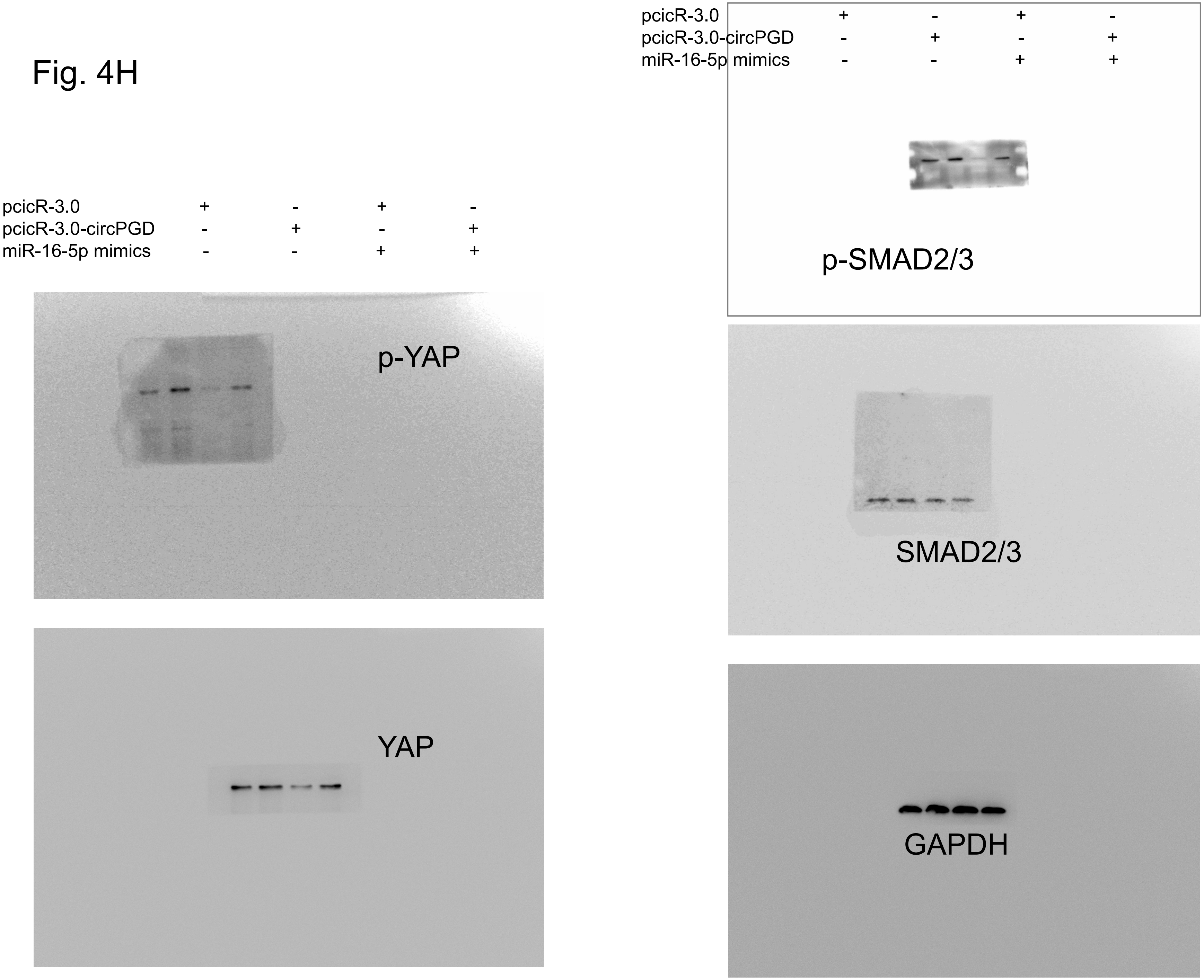
pcicR-3.0 + - + -
pcicR-3.0-circPGD - + - +
miR-16-5p mimics - - + +
p-YAP
YAP
pcicR-3.0 + - + -
pcicR-3.0-circPGD - + - +
miR-16-5p mimics - - + +
p-SMAD2/3
SMAD2/3
GAPDH

Fig. 4N

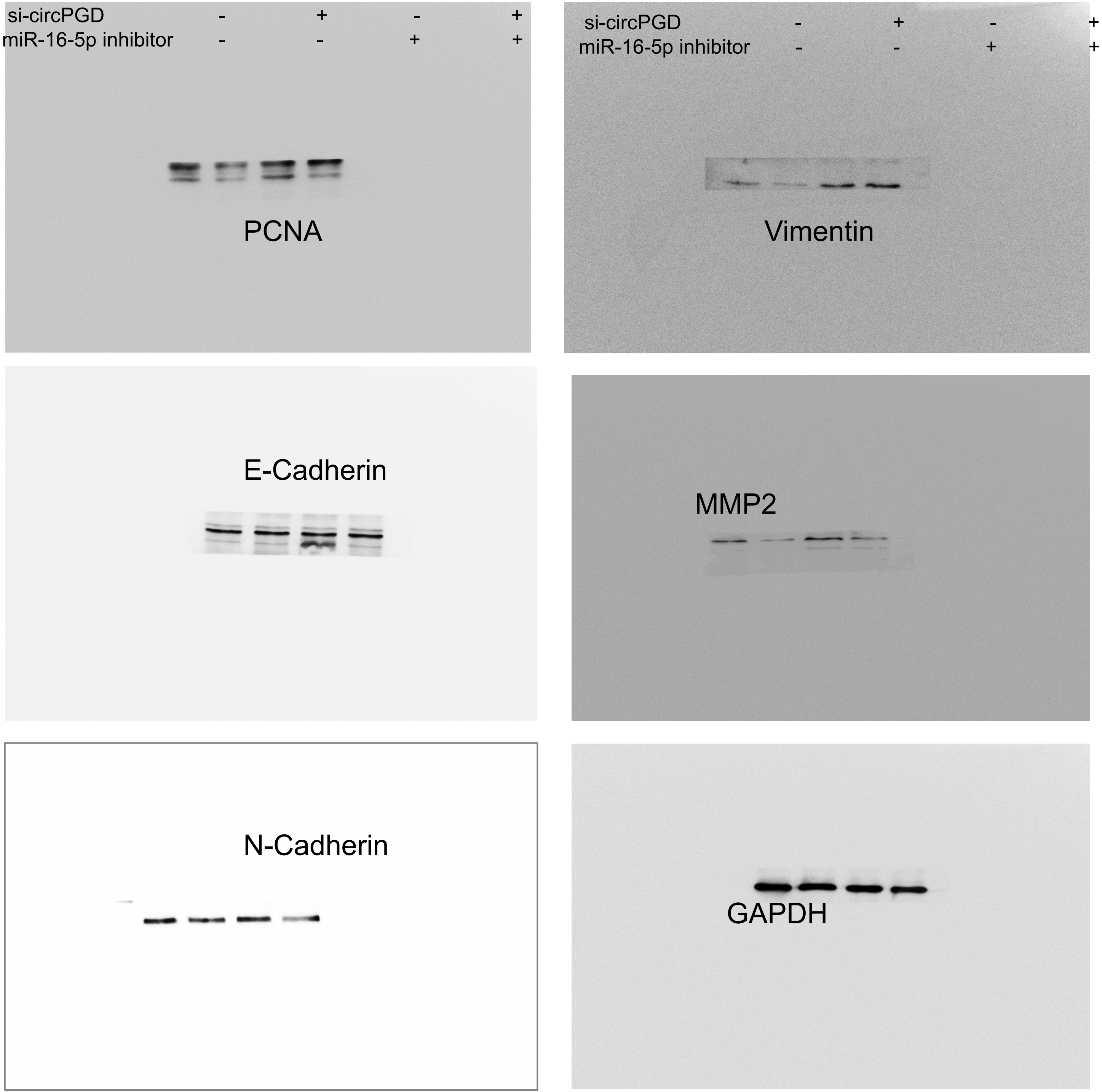
si-circPGD - + - +
miR-16-5p inhibitor - - + +
PCNA
si-circPGD - + - +
miR-16-5p inhibitor - - + +
Vimentin
E-Cadherin
MMP2
N-Cadherin
GAPDH

Fig. 4O

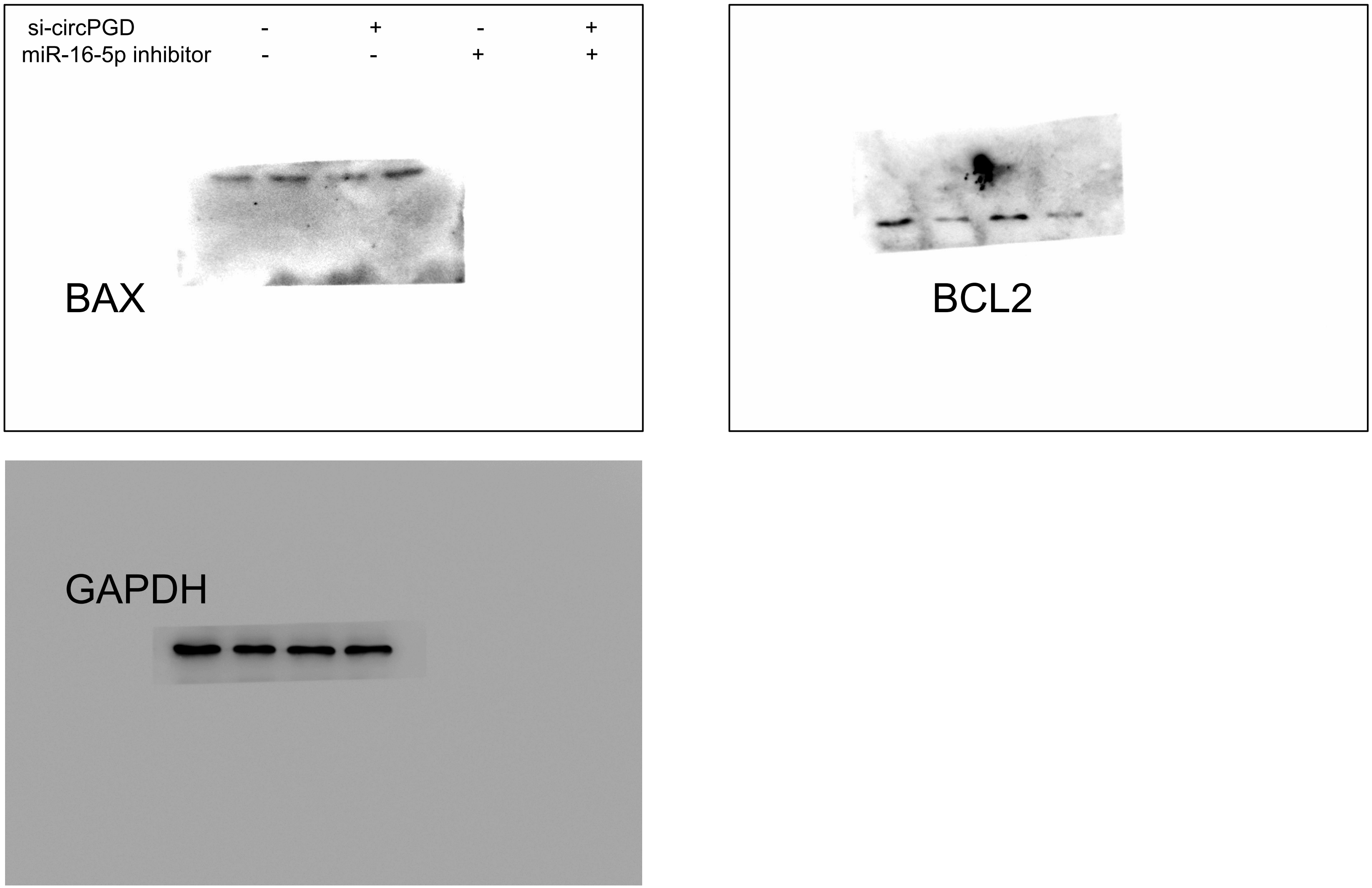

Fig. 4P

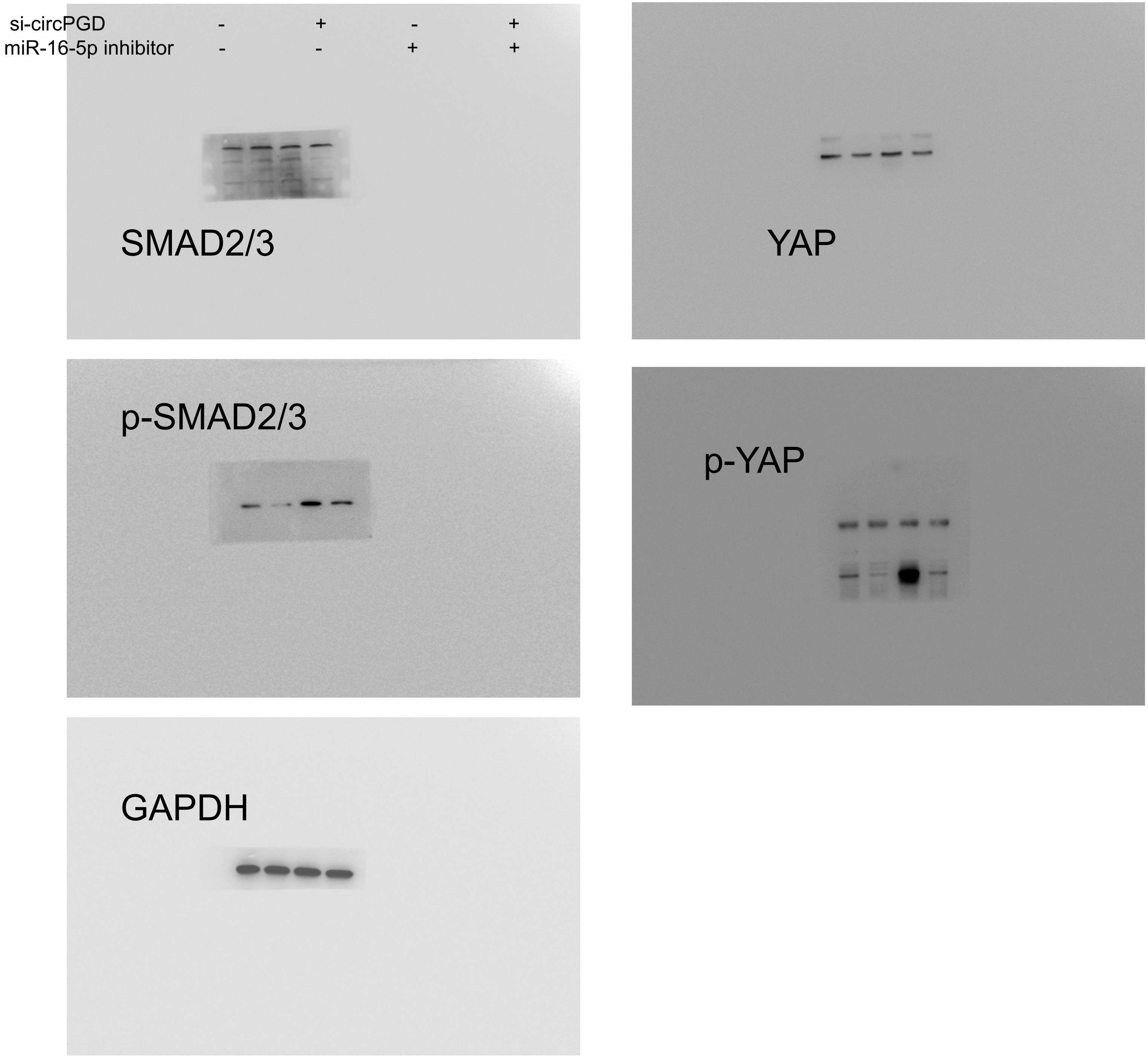
si-circPGD - + - +
miR-16-5p inhibitor - - + +
SMAD2/3
YAP
p-SMAD2/3
p-YAP
GAPDH

Fig 5B

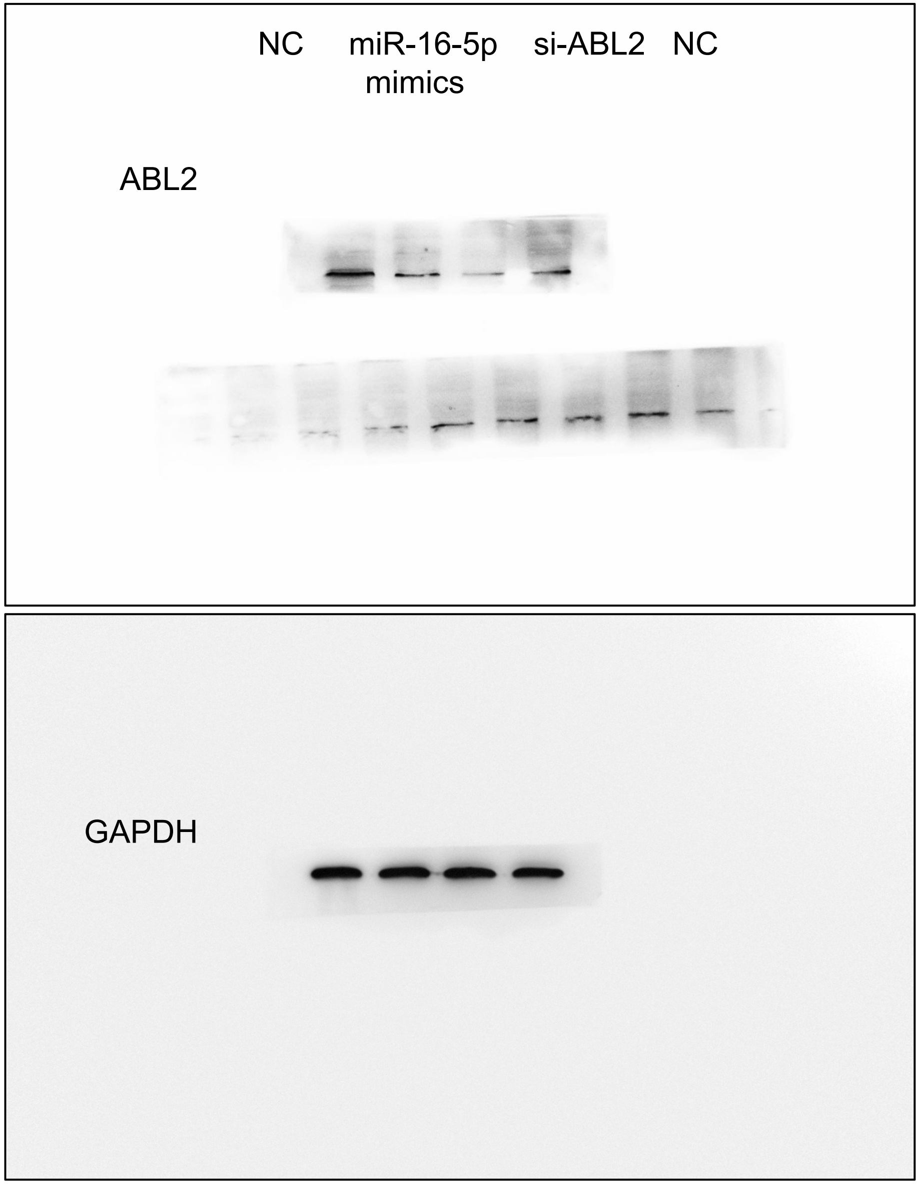
NC
miR-16-5p mimics
si-ABL2
NC
ABL2
GAPDH

Fig 5C

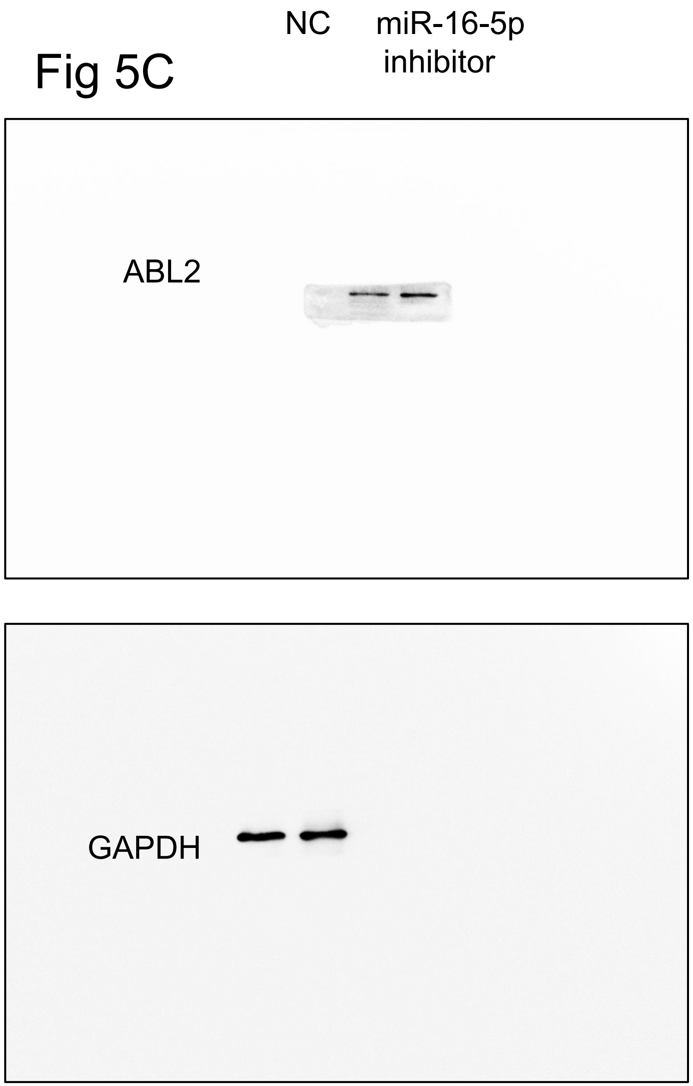
NC
miR-16-5p inhibitor
ABL2
GAPDH

Fig. 5E

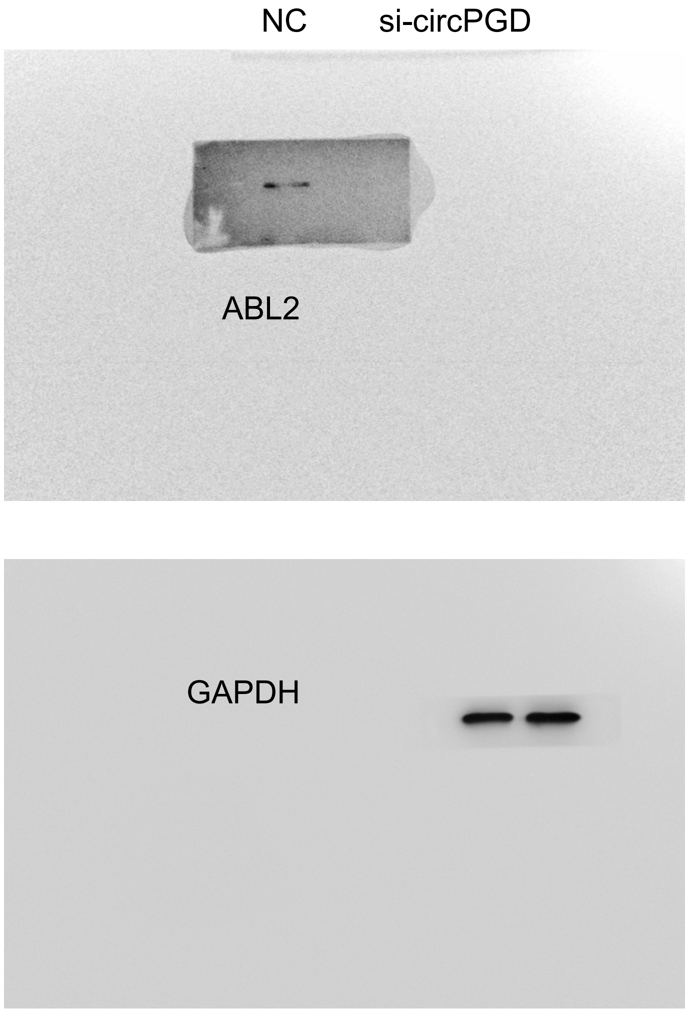

Fig. 5D

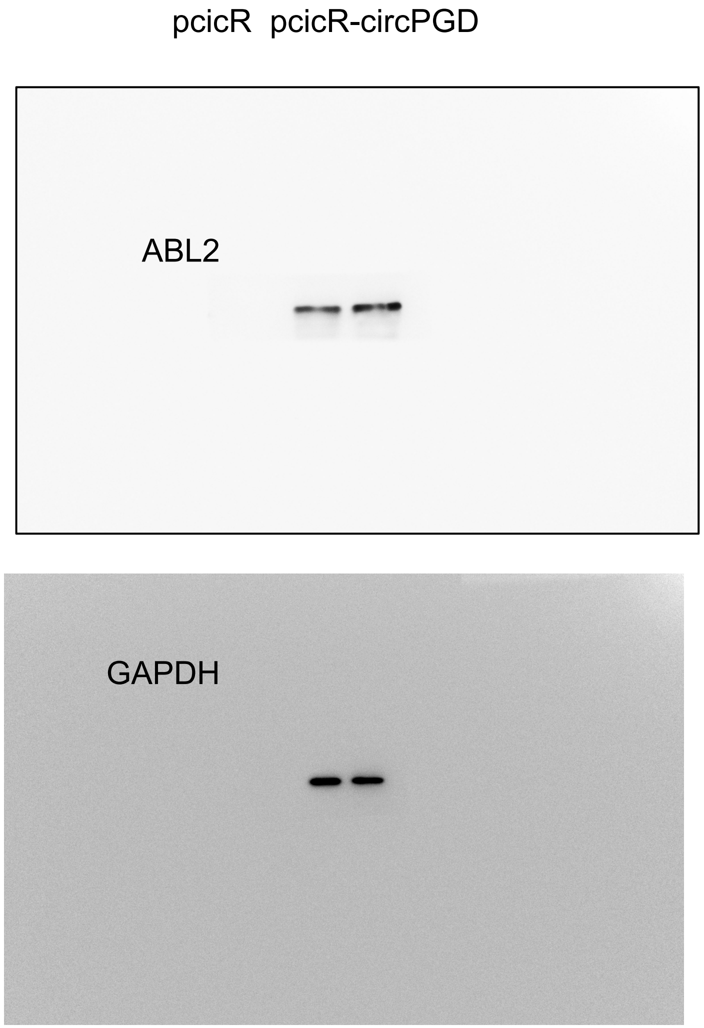

Fig 5I

pcicR-3.0	+	-	+	-
pcicR-3.0-circPGD	-	+	-	+
miR-16-5p mimics	-	-	+	+

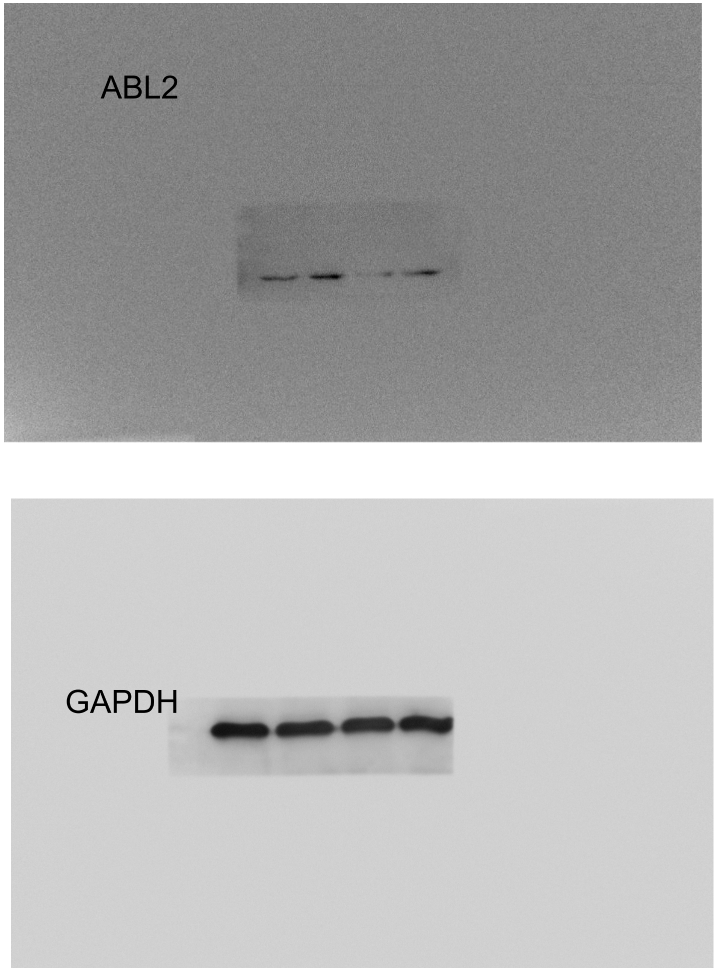

Fig 5J

si-circPGD	-	+	-	+
miR-16-5p inhibitor	-	-	+	+

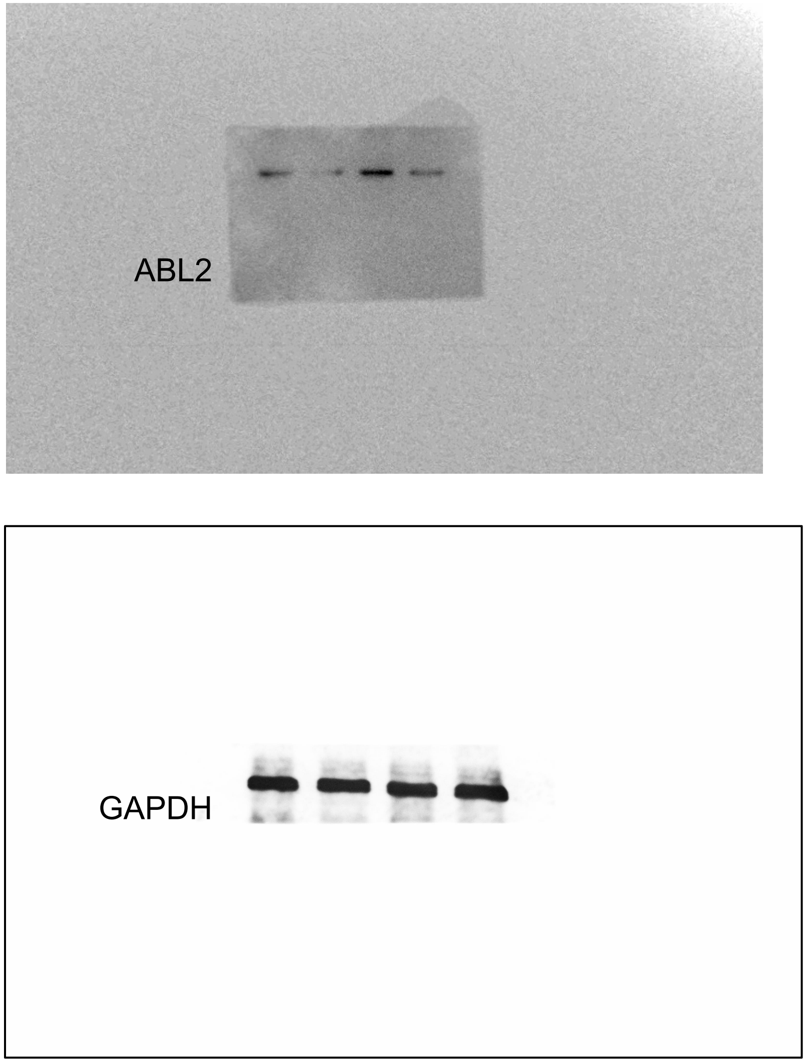

Fig. 5O

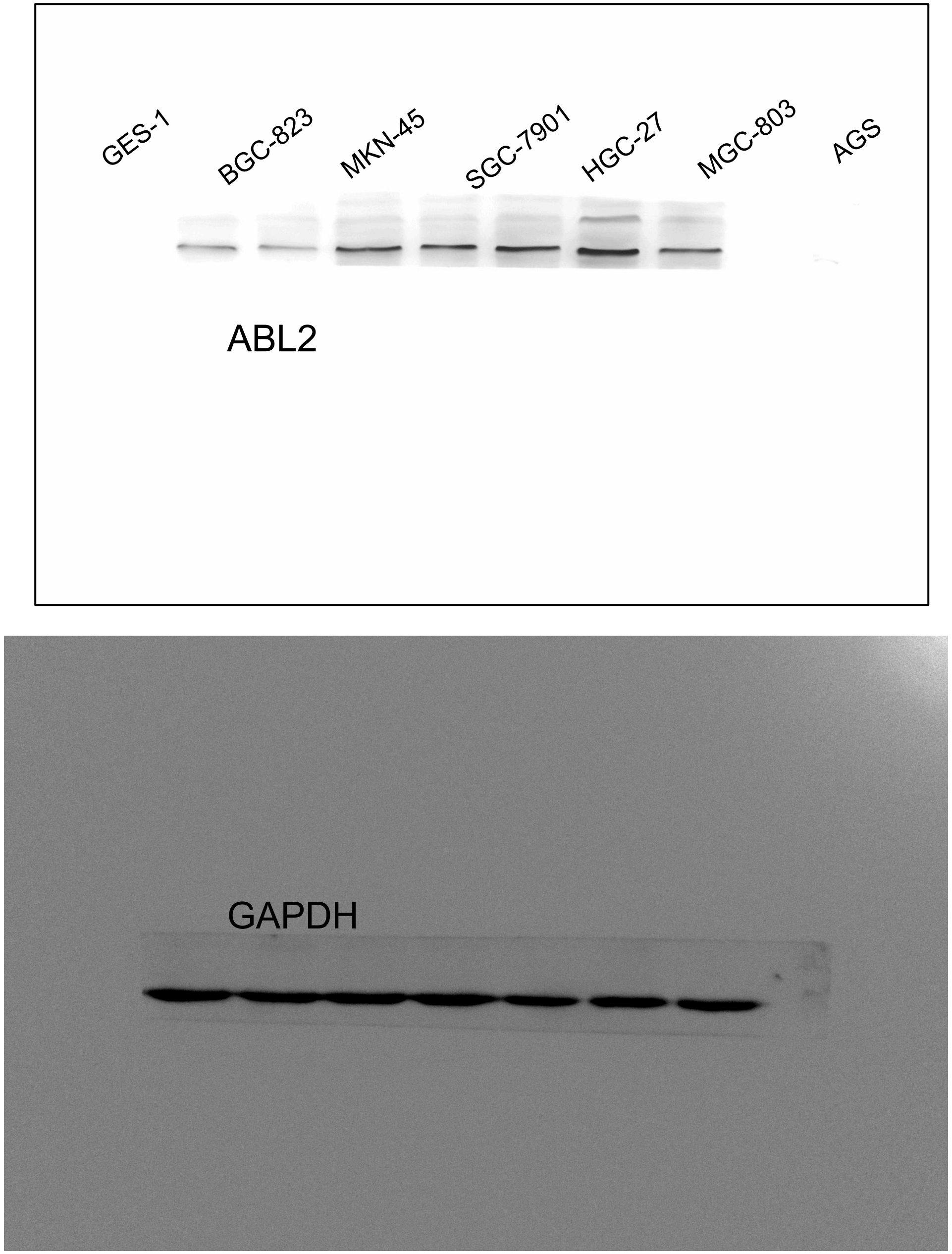

Fig. 6F

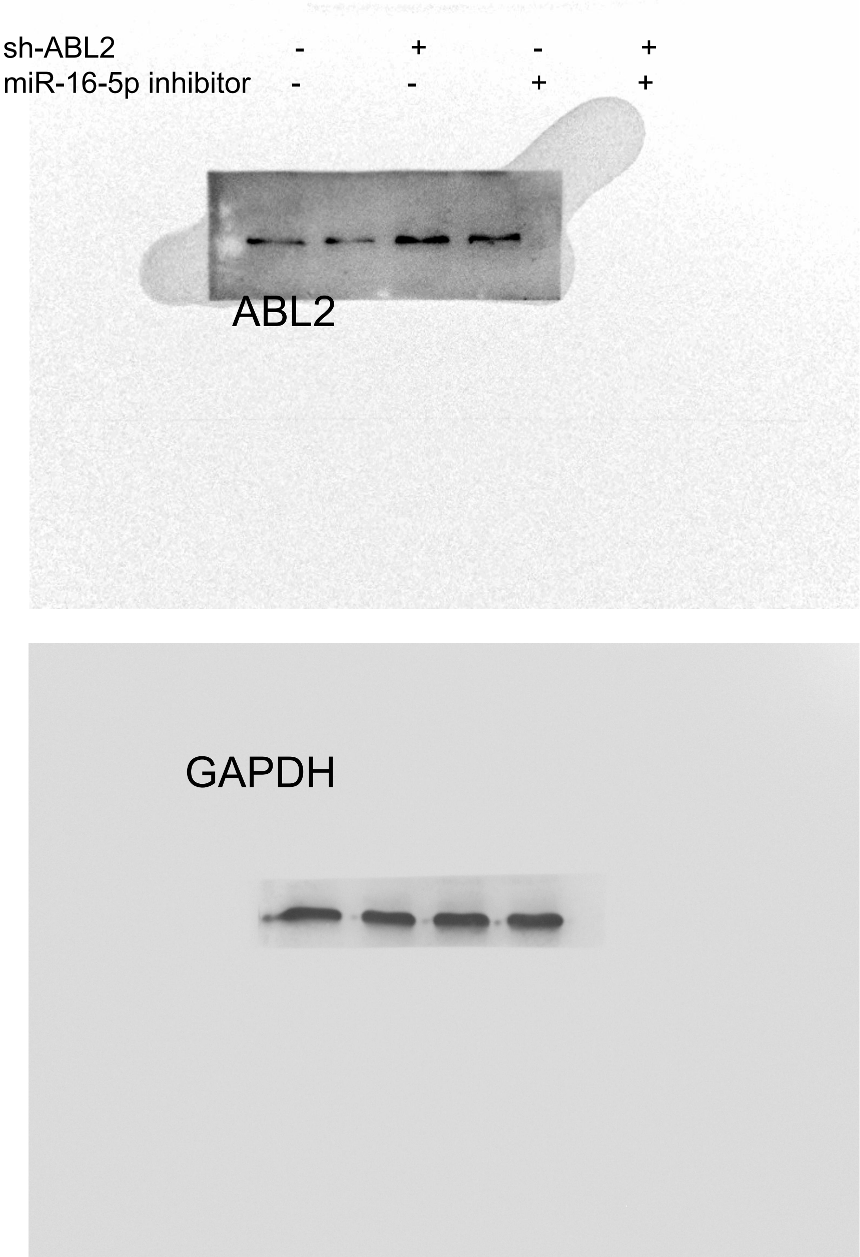

Fig. 6G

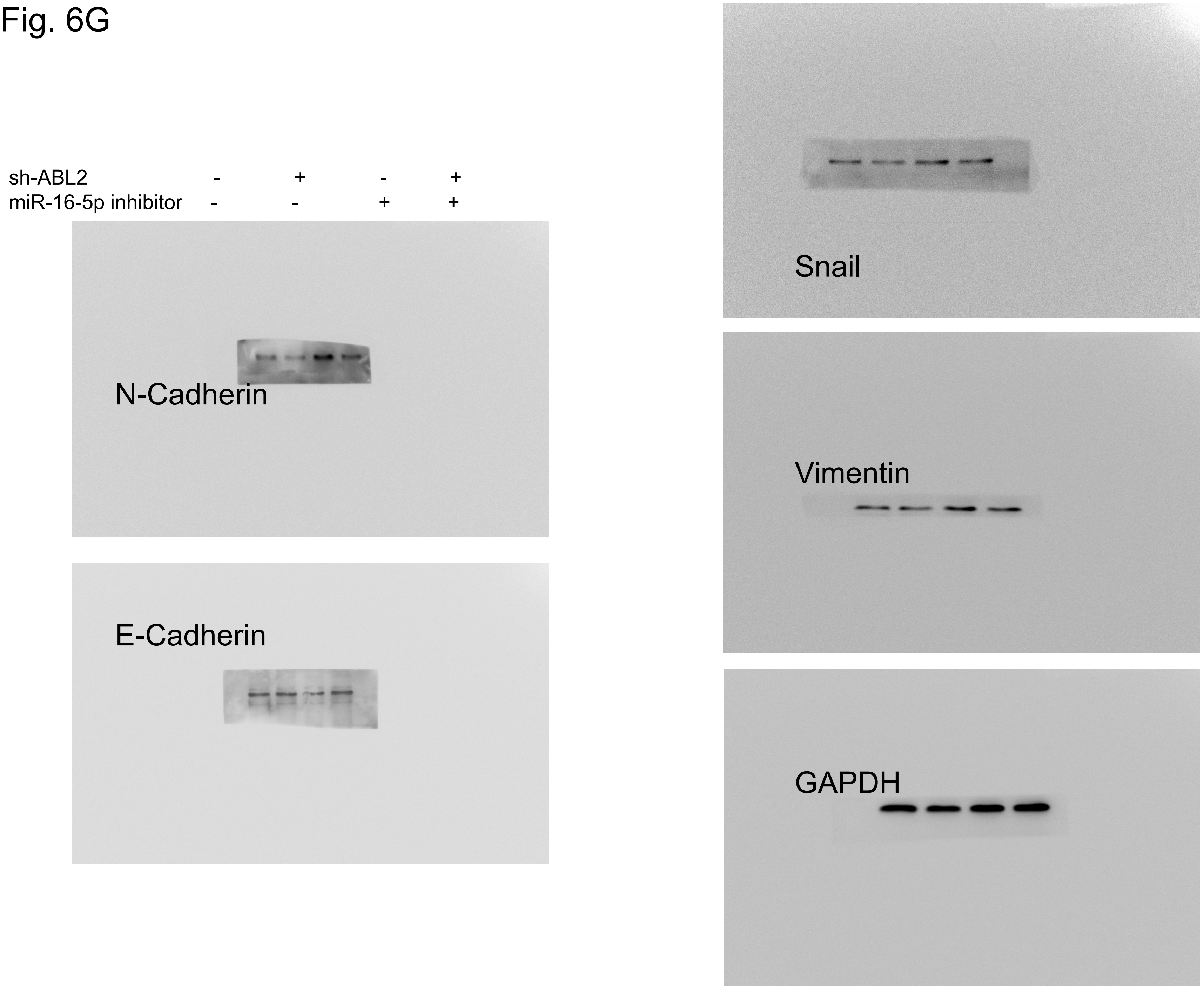
sh-ABL2 - + - +
miR-16-5p inhibitor - - + +
N-Cadherin
E-Cadherin
Snail
Vimentin
GAPDH

Fig. 6H

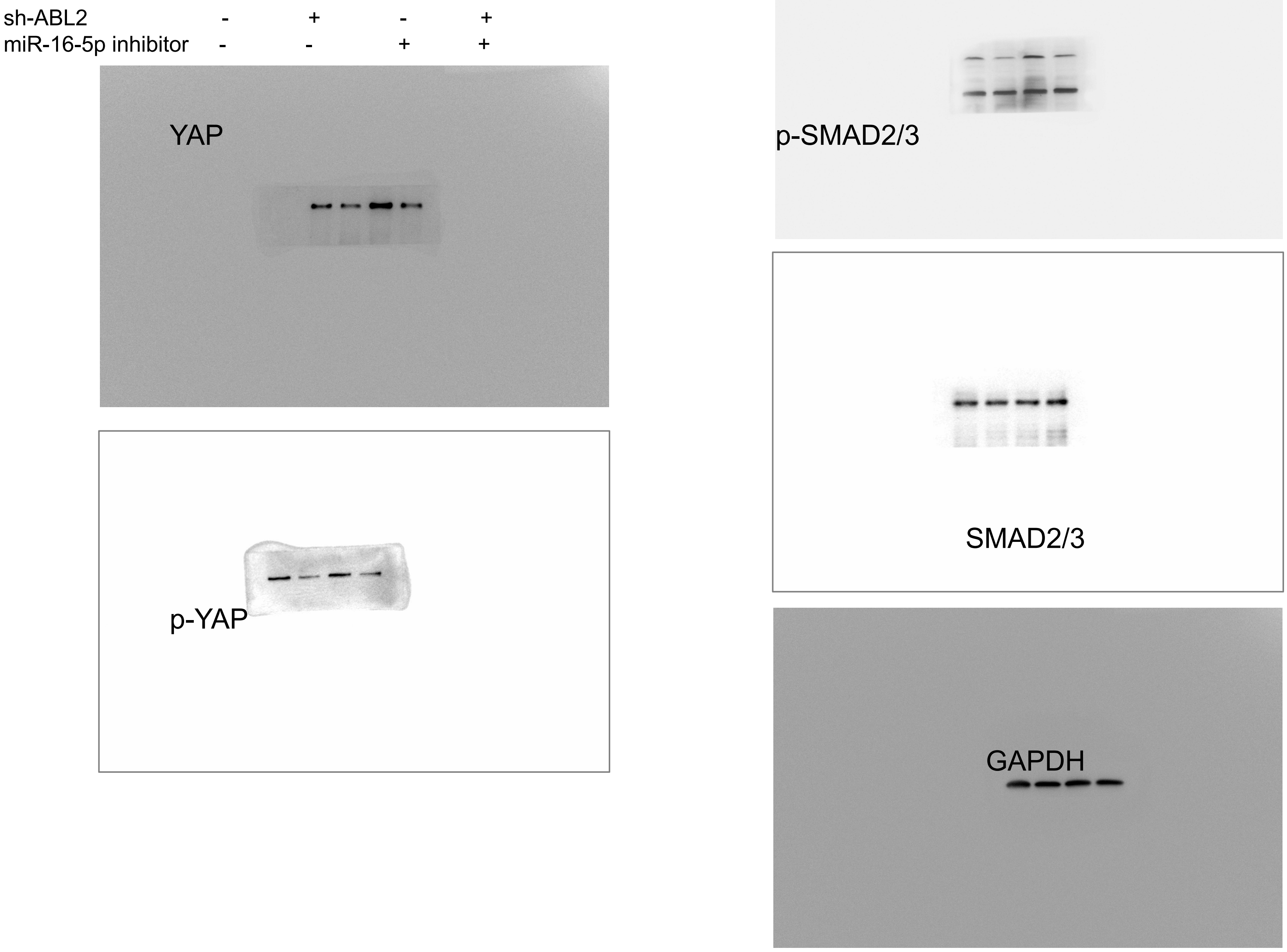

Fig. 6L

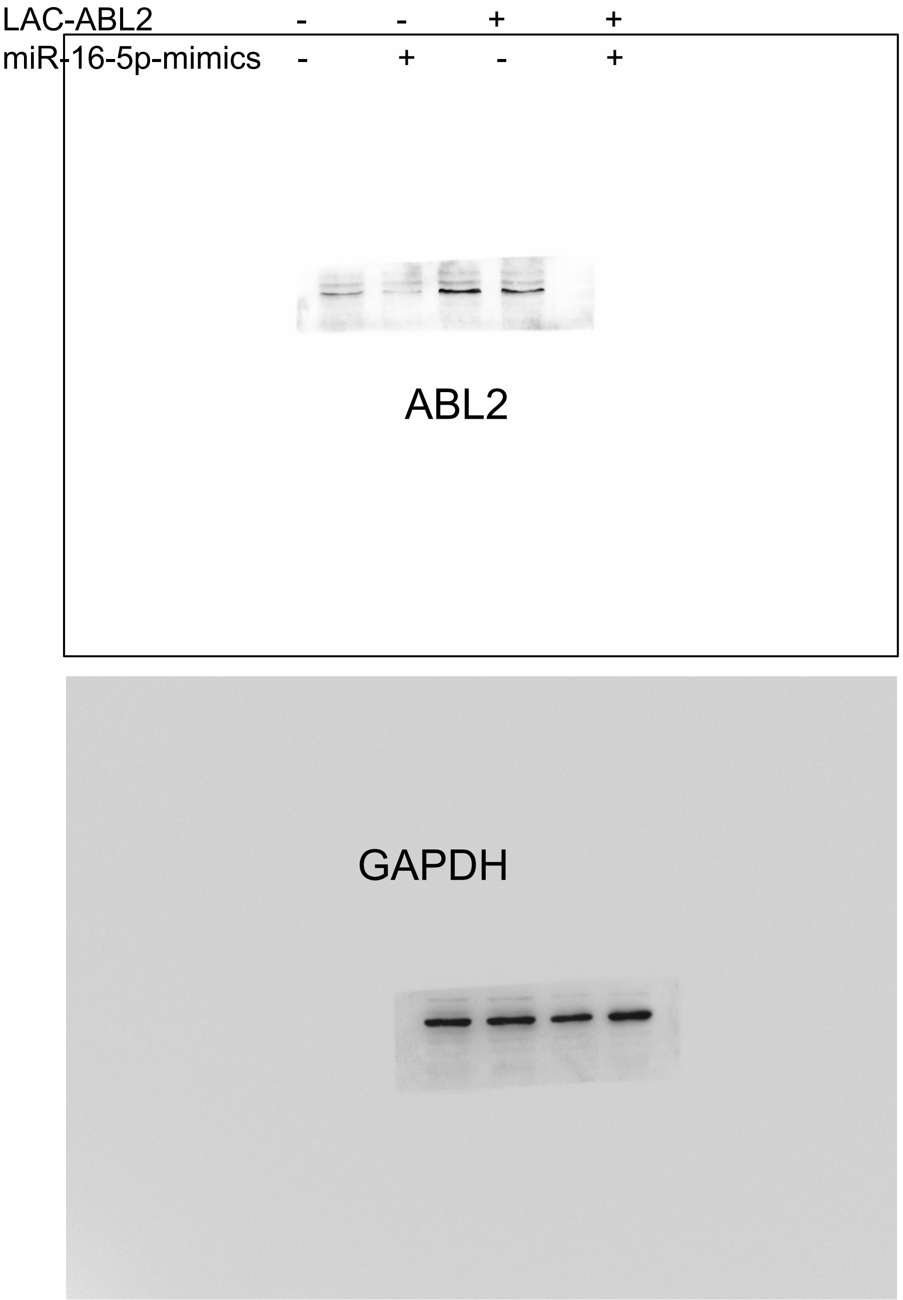

Fig. 6M

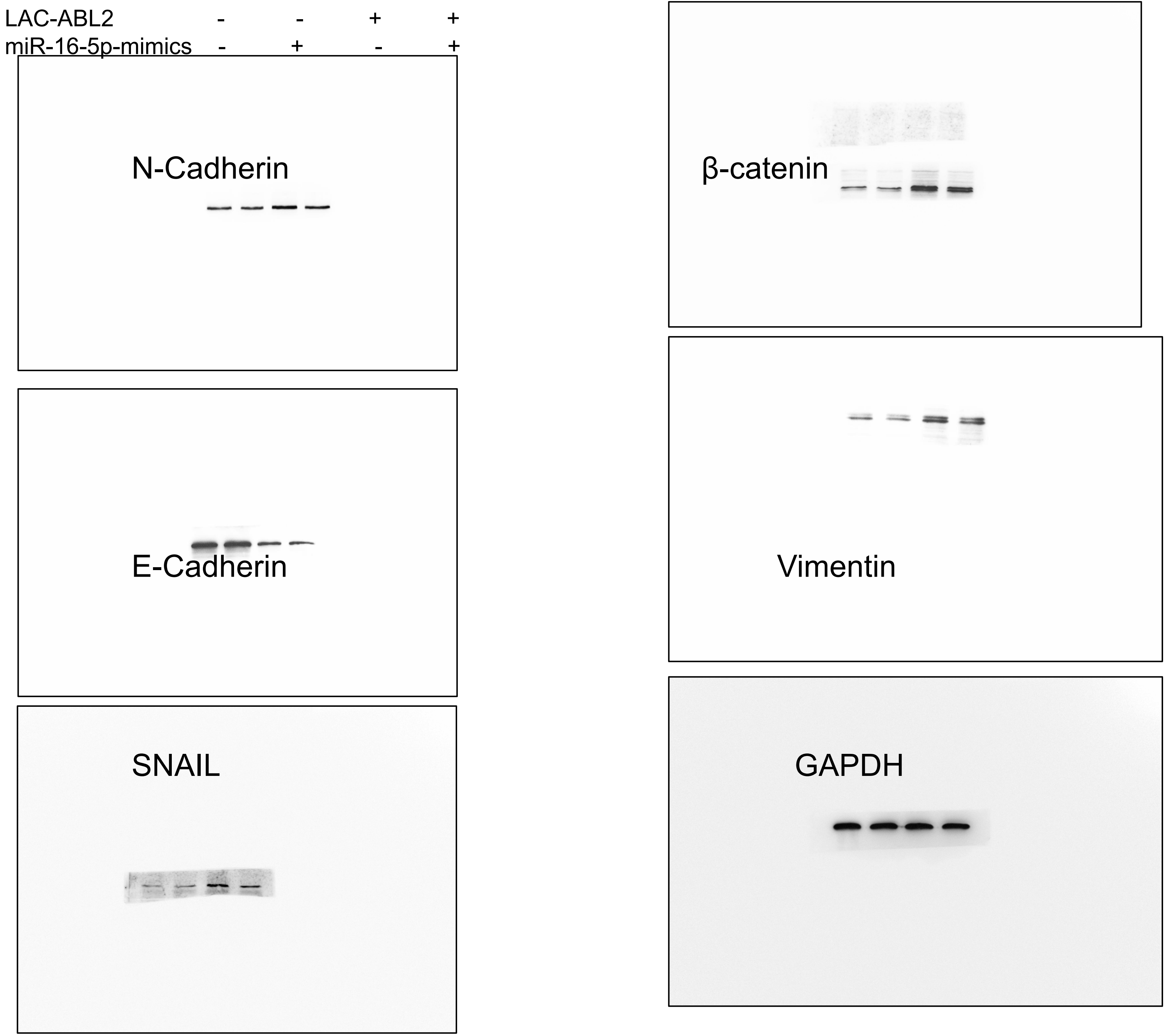
LAC-ABL2 - - + +
miR-16-5p-mimics - + - +
N-Cadherin
E-Cadherin
SNAIL
β-catenin
Vimentin
GAPDH

Fig. 6N

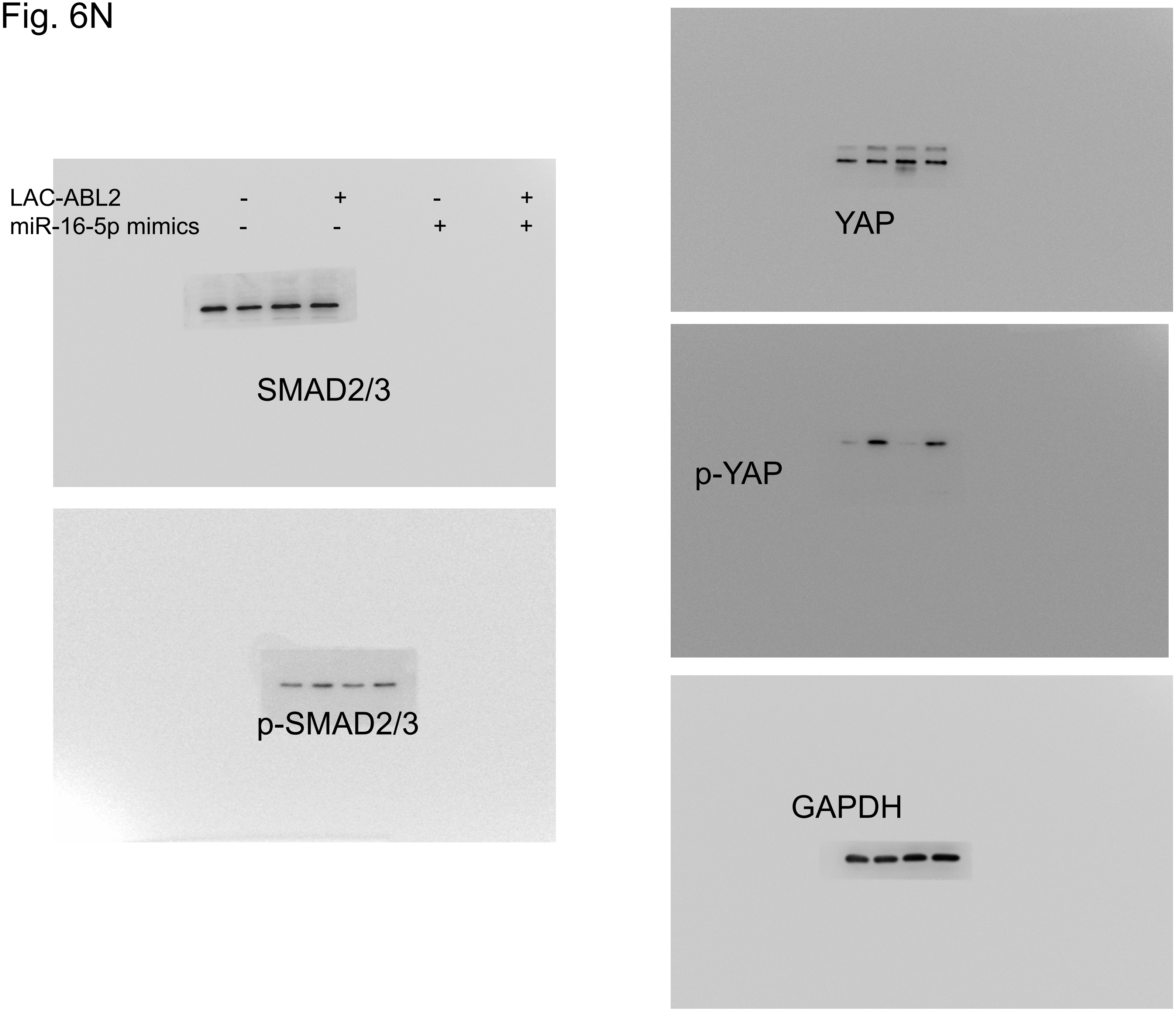

Fig. 7D

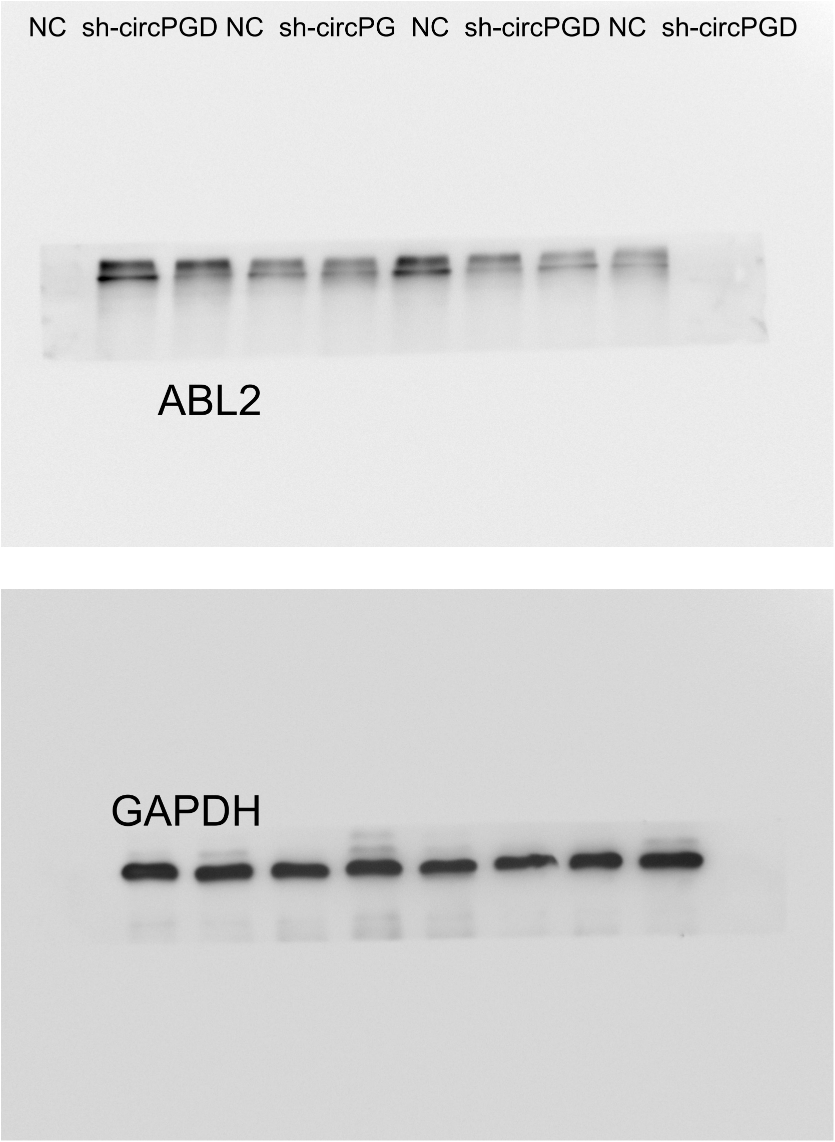

NC sh-circPGD NC sh-circPG NC sh-circPGD NC sh-circPGD
ABL2
GAPDH

Fig. 7E

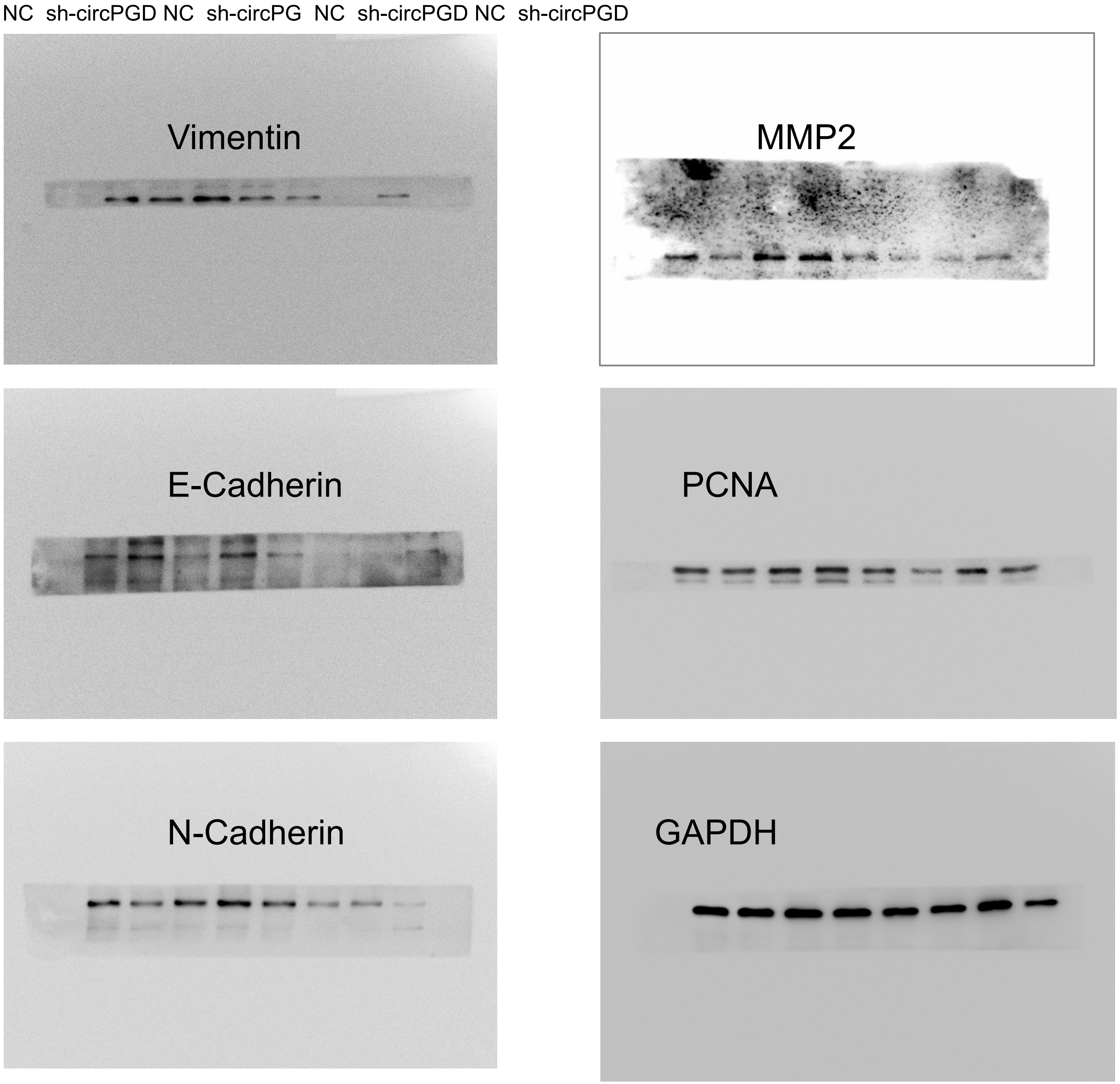
NC sh-circPGD NC sh-circPG NC sh-circPGD NC sh-circPGD
Vimentin
MMP2
E-Cadherin
PCNA
N-Cadherin
GAPDH

Fig. 7G

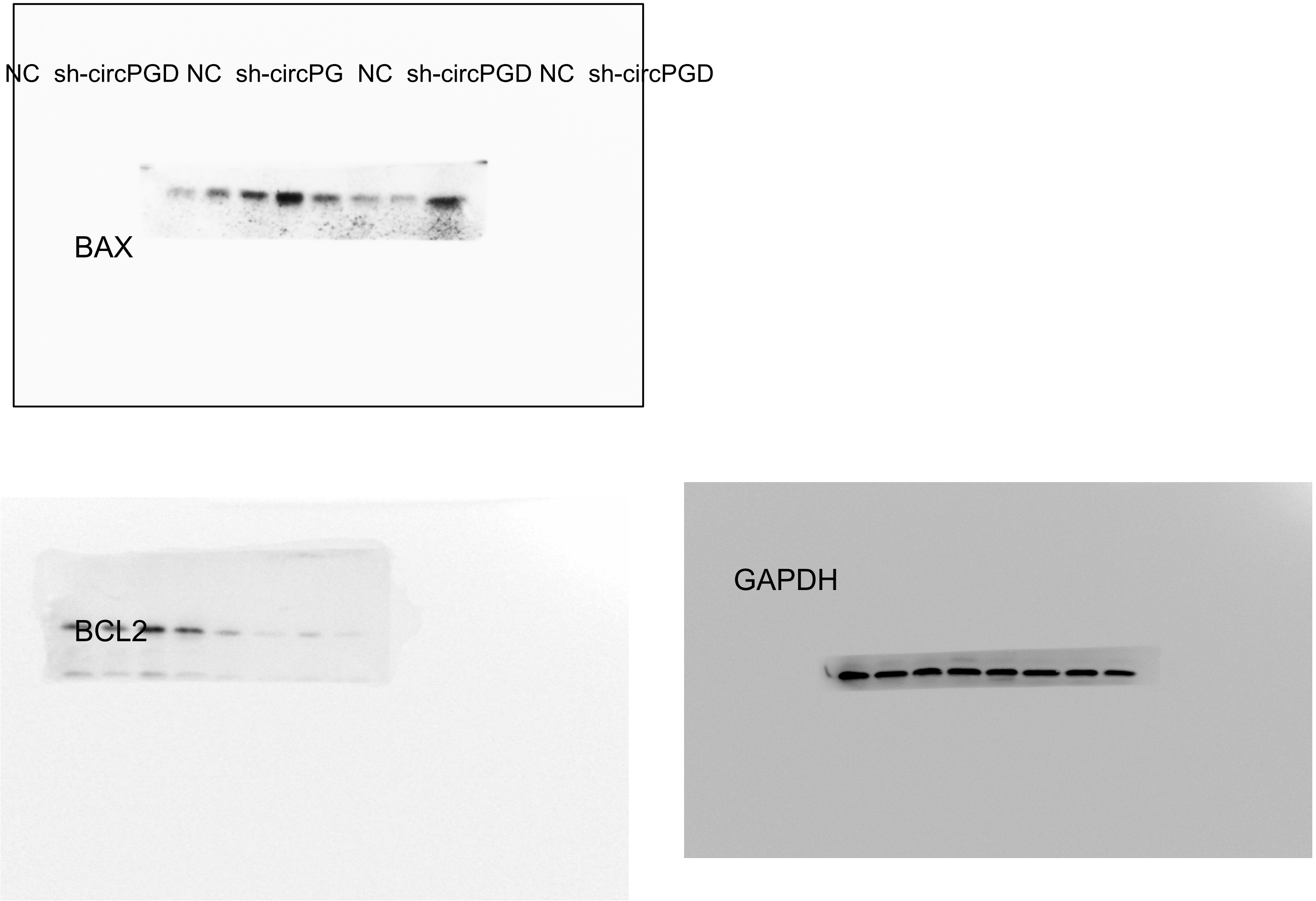

NC sh-circPGD NC sh-circPG NC sh-circPGD NC sh-circPGD
BAX
BCL2
GAPDH

Fig. 7H

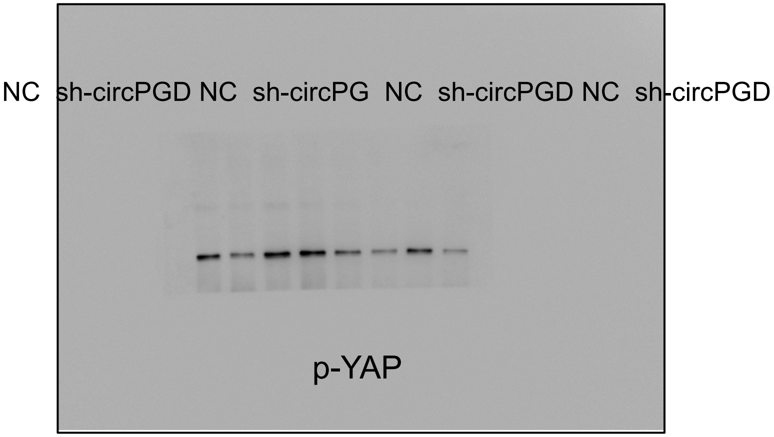
NC sh-circPGD NC sh-circPG NC sh-circPGD NC sh-circPGD
p-YAP

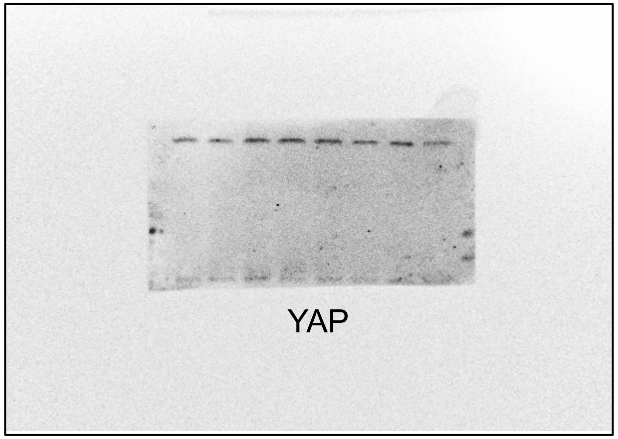
YAP

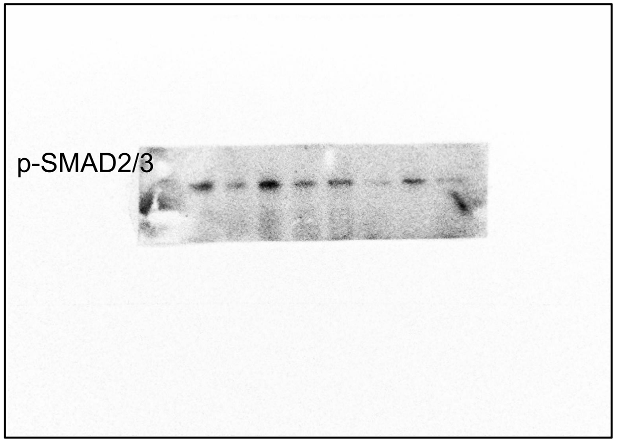
p-SMAD2/3

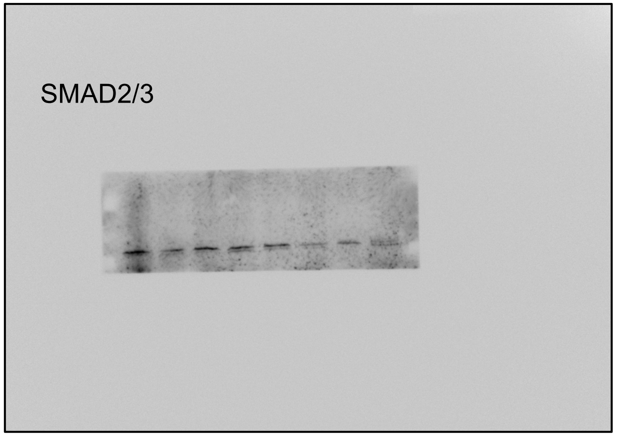
SMAD2/3

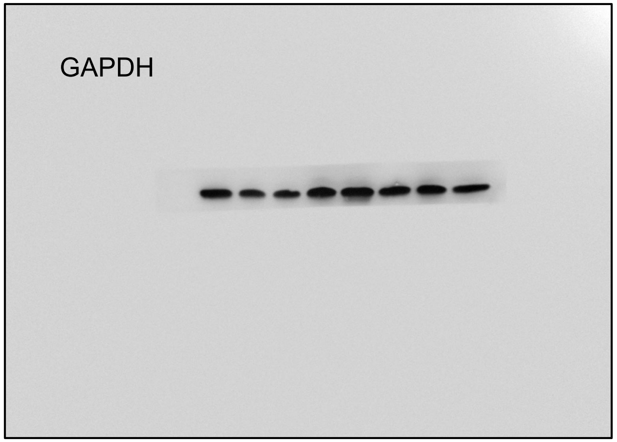
GAPDH

Fig. 7L

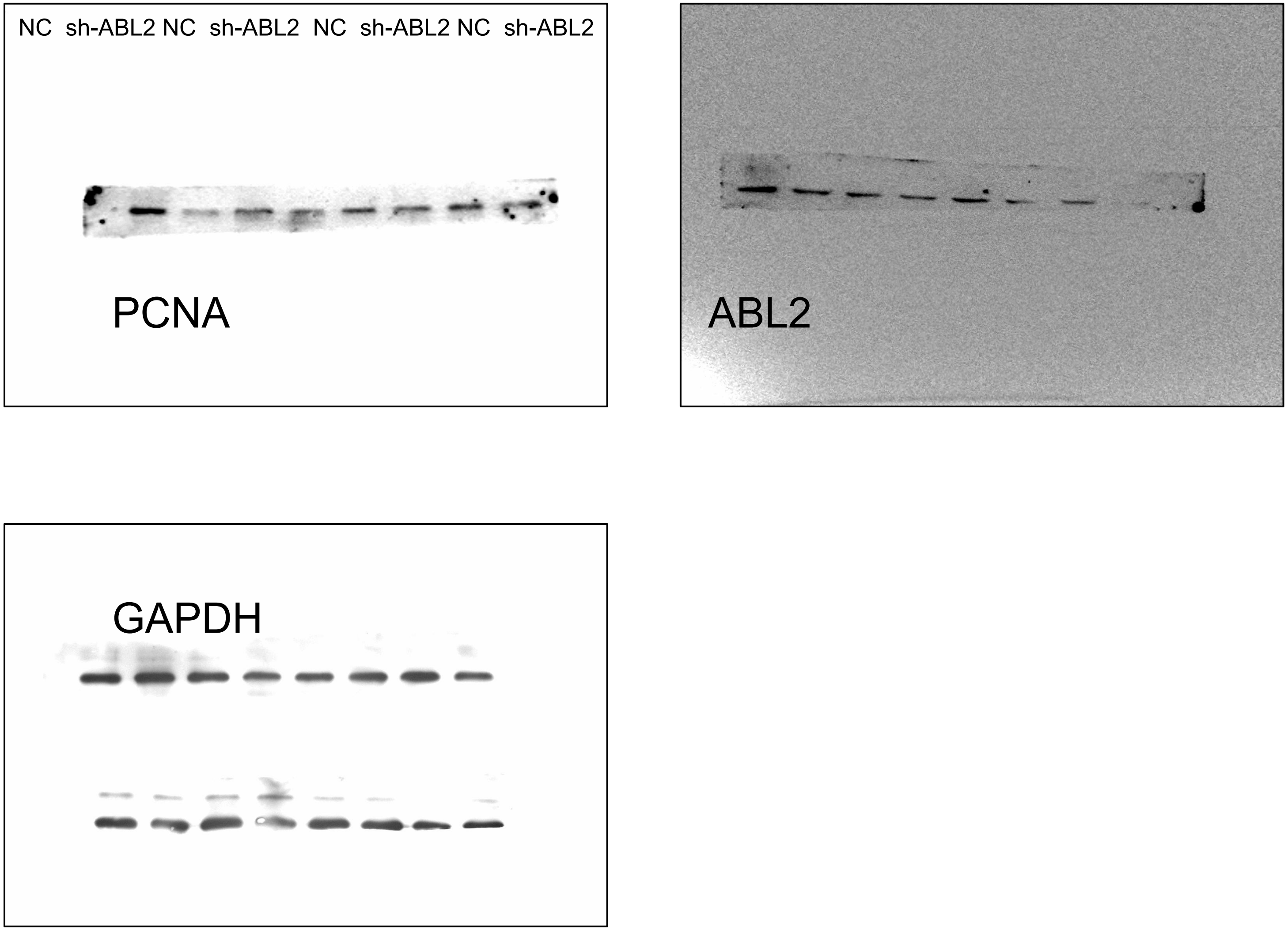

Fig. 8B

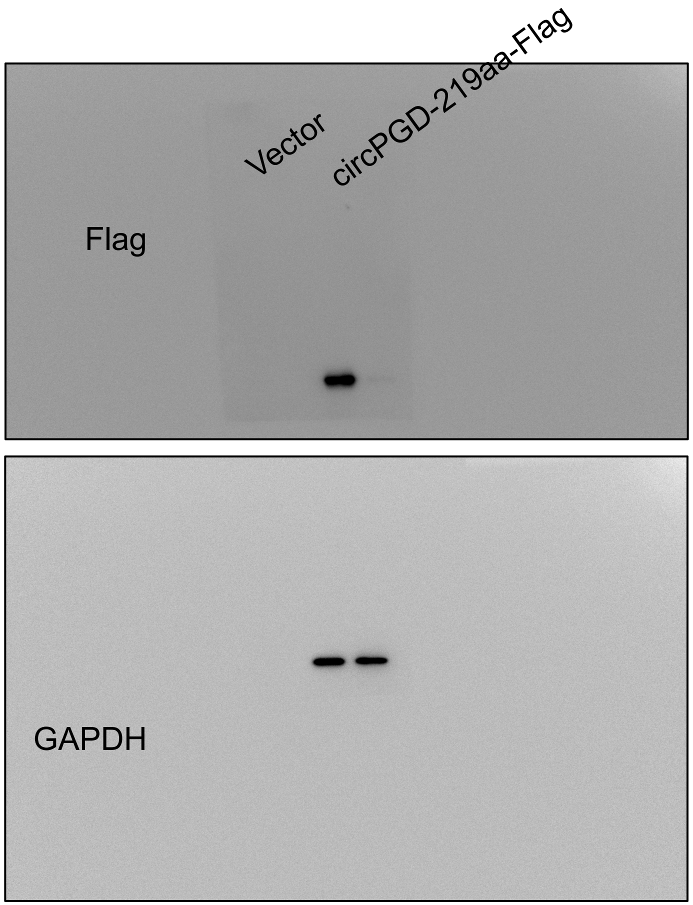

Fig. 8C

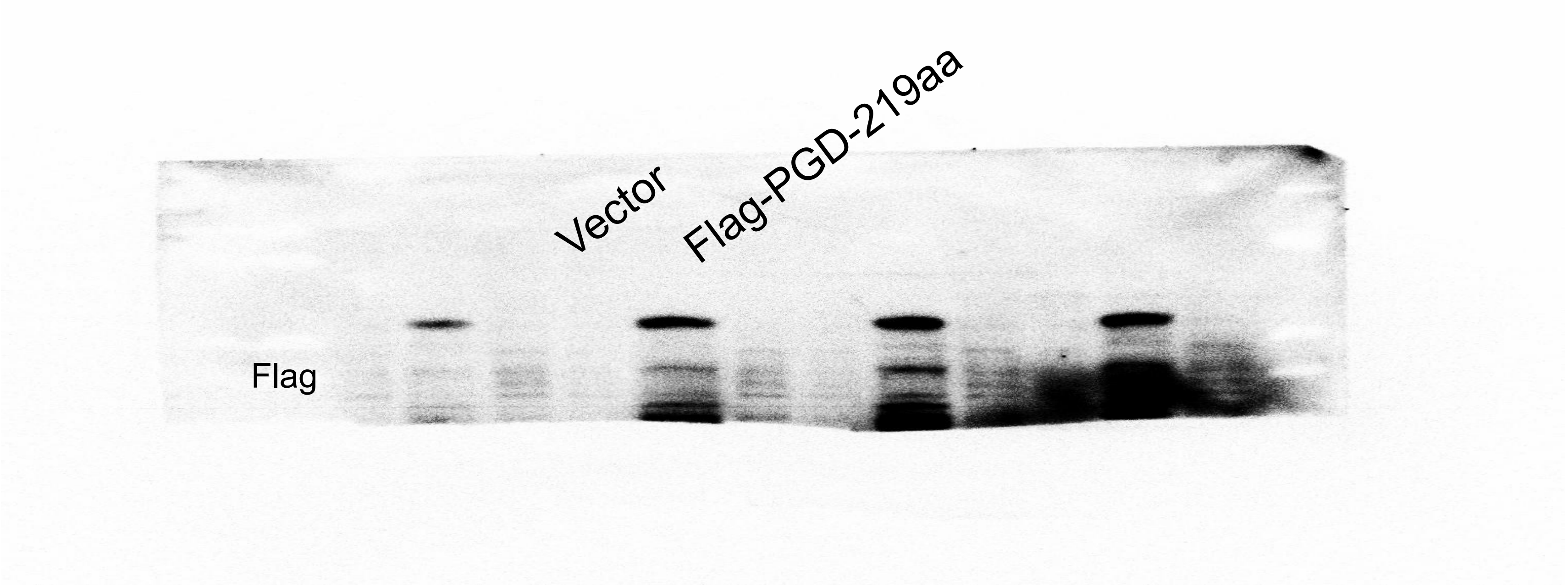

Fig. 8J

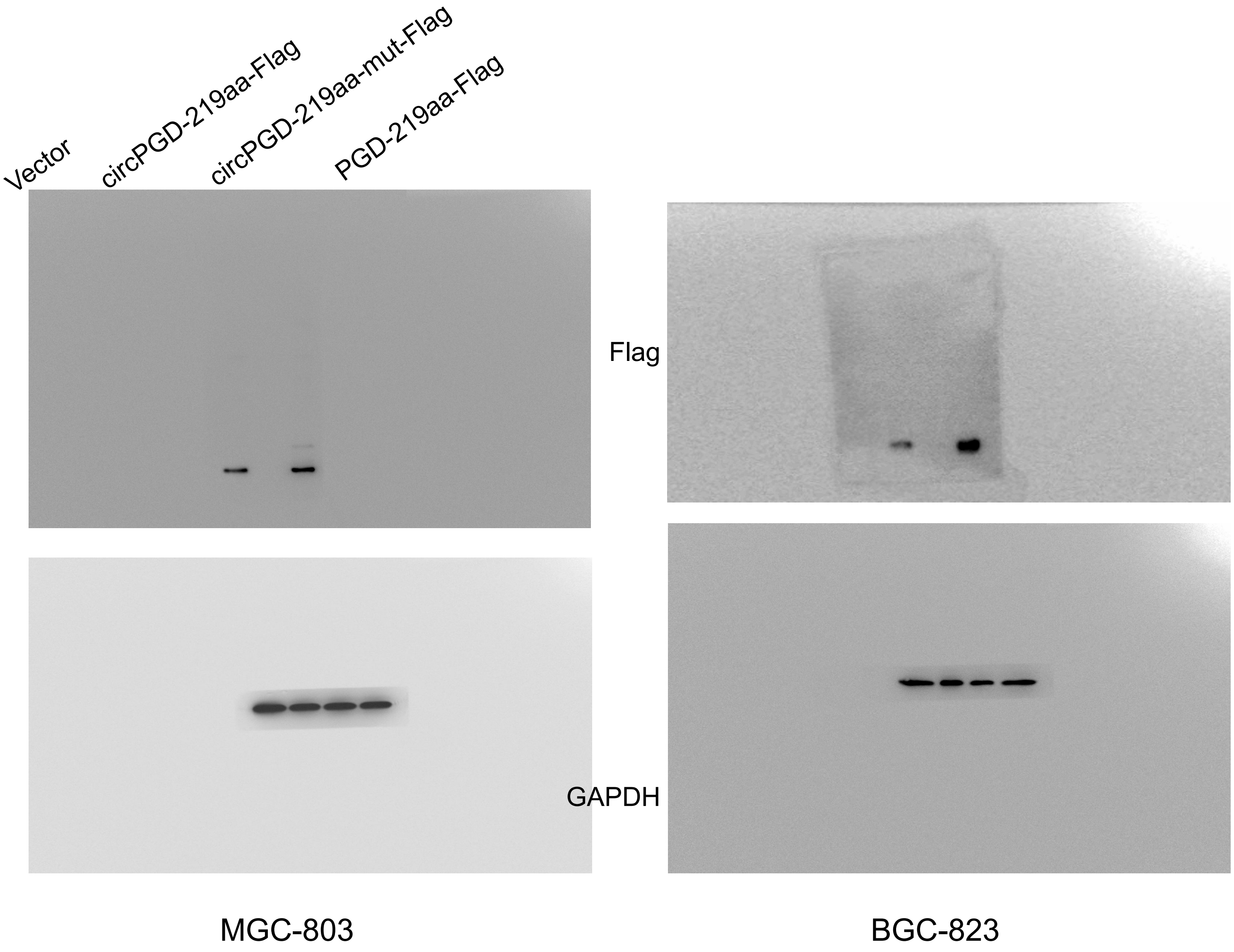
Vector
circPGD-219aa-Flag
circPGD-219aa-mut-Flag
PGD-219aa-Flag
Flag
GAPDH
MGC-803
BGC-823

Fig. 8P MGC-803 cells

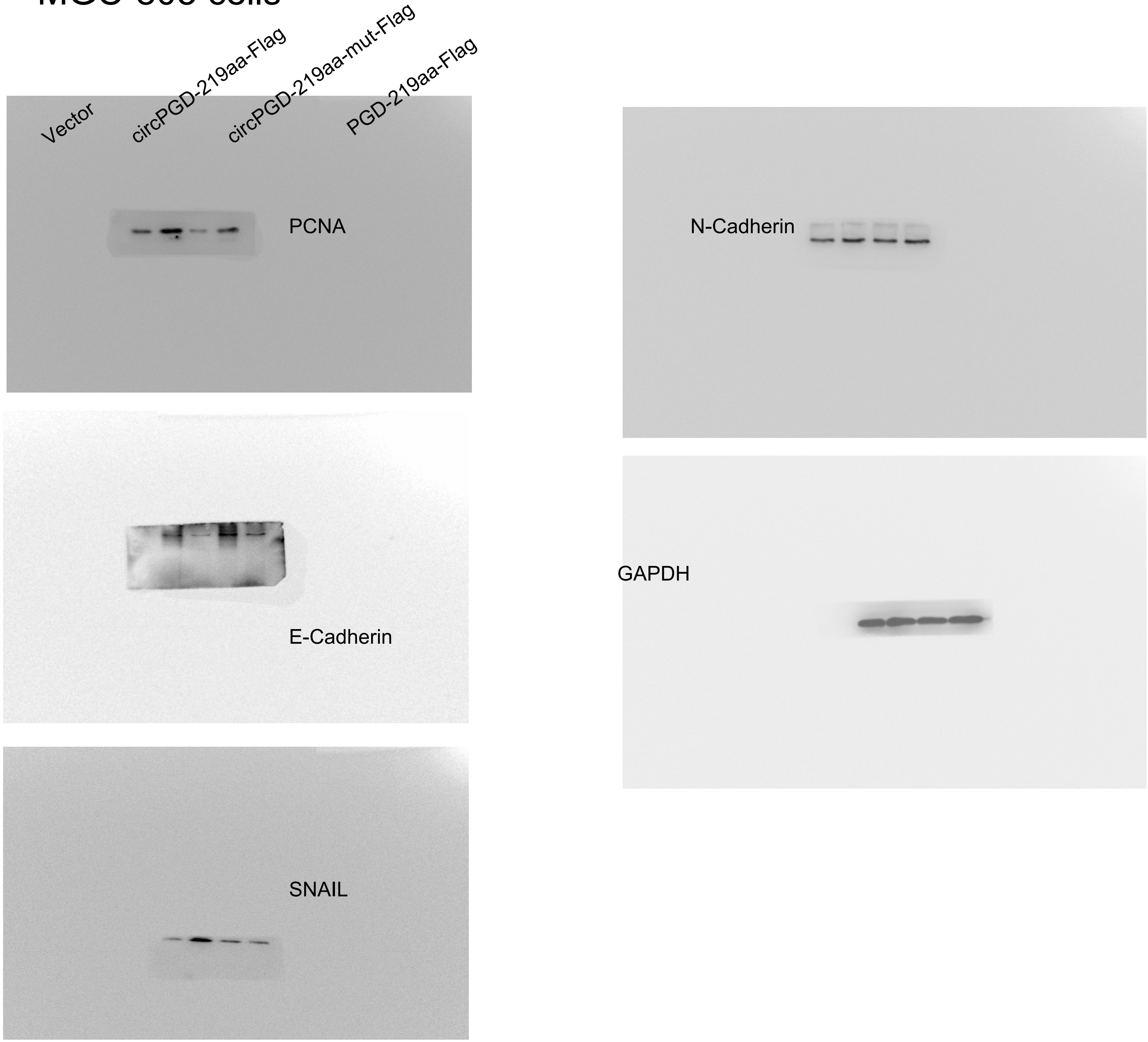

Fig. 8P BGC-823 cells

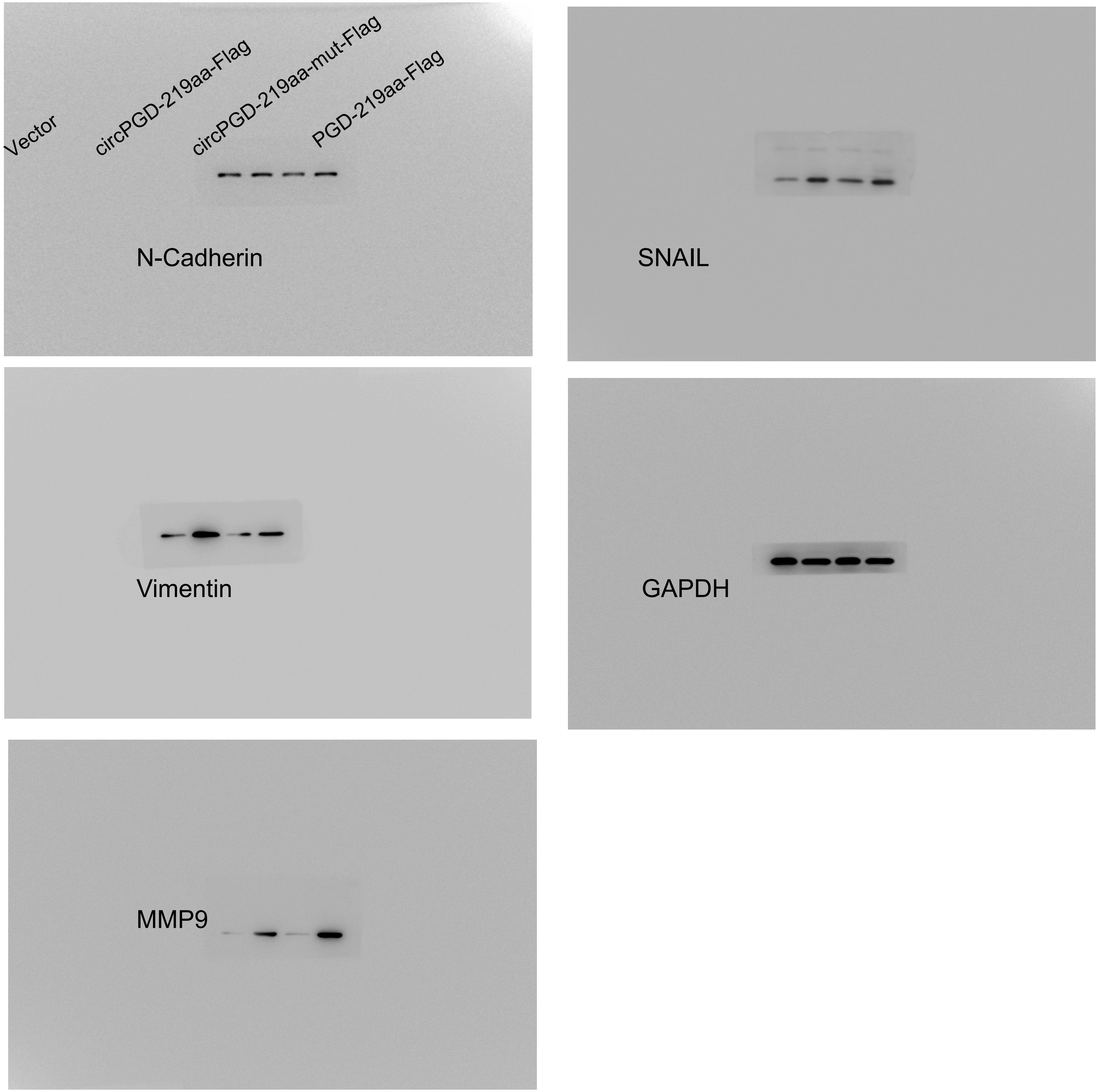

Fig. 8Q MGC-803 cells

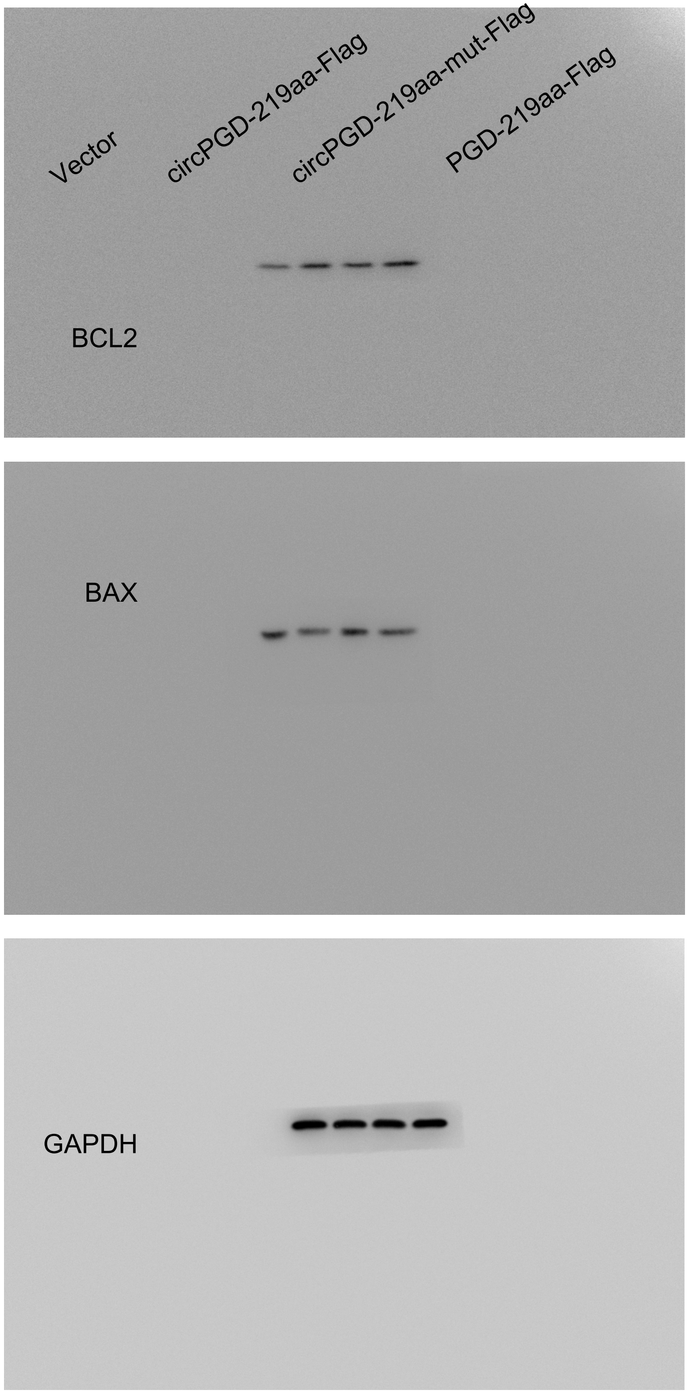

Fig.8Q BGC-823 cells

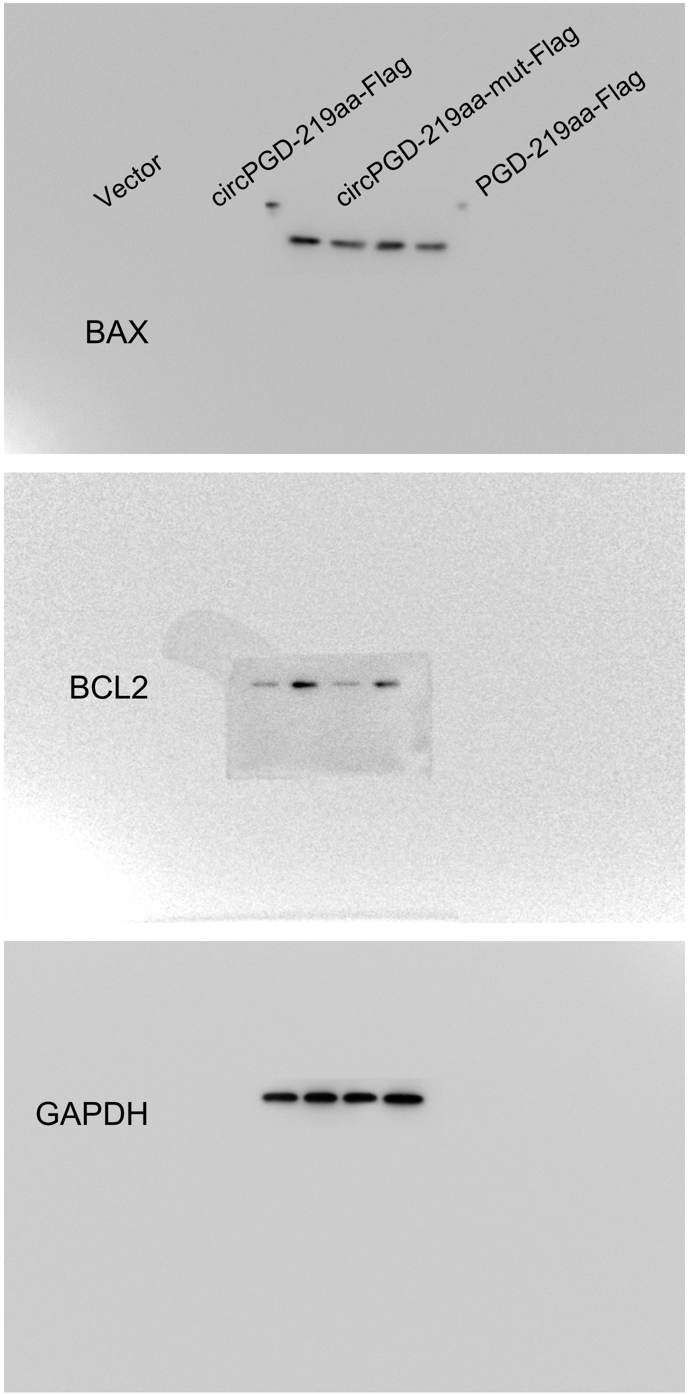

Fig. 8R MGC-803 cells

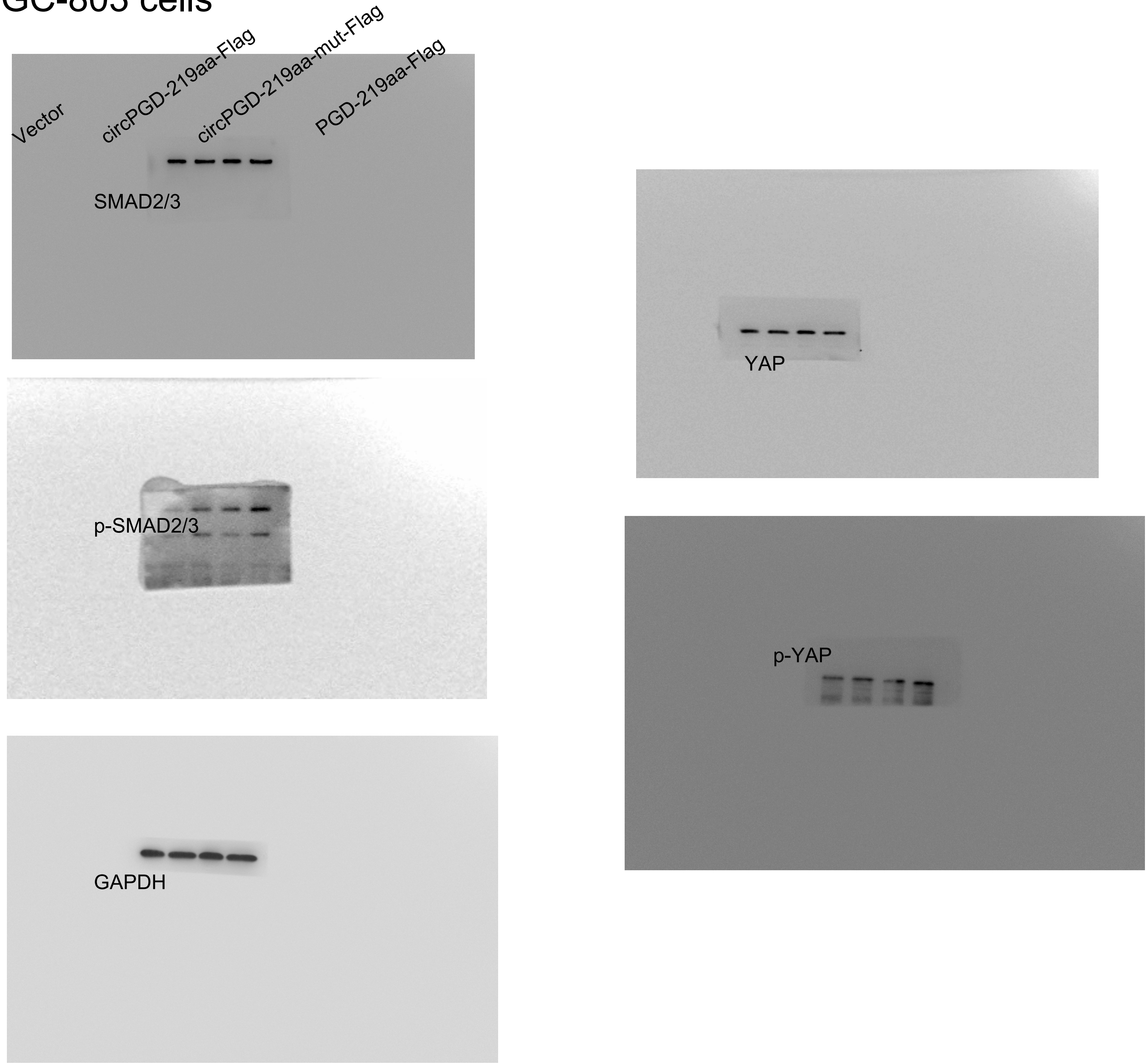

Fig. 8R BGC-823 cells

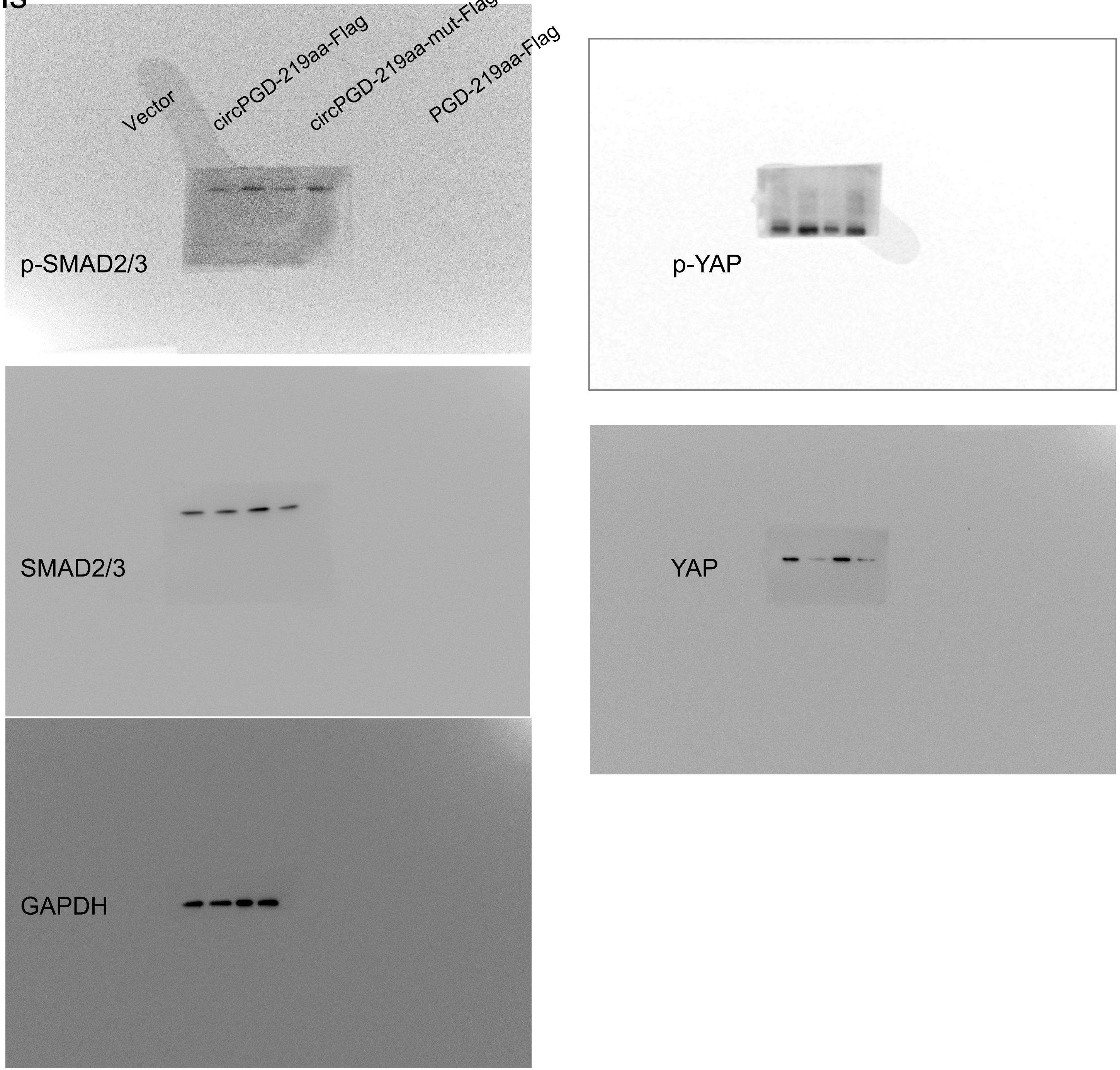

Fig. S4

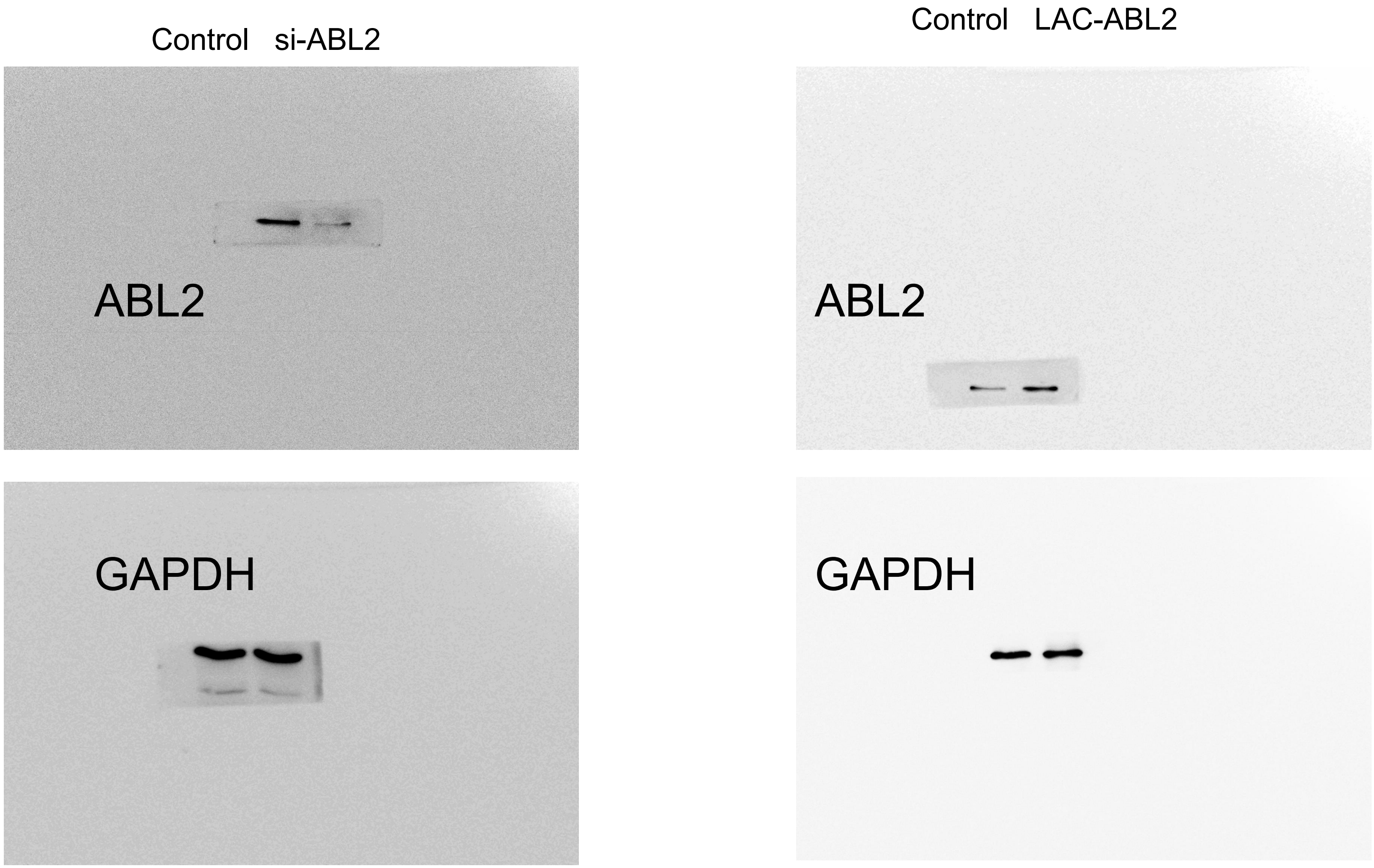

Supplement: Supplementary file 10 — Full uncut western blots [file 41420_2022_1177_MOESM10_ESM.pdf]
